# Resolving Femtosecond Solvent Reorganization Dynamics in an Iron Complex by Nonadiabatic Dynamics Simulations

**DOI:** 10.1021/jacs.2c04505

**Published:** 2022-07-01

**Authors:** Diana Bregenholt Zederkof, Klaus B. Møller, Martin M. Nielsen, Kristoffer Haldrup, Leticia González, Sebastian Mai

**Affiliations:** † Department of Physics, Technical University of Denmark, Fysikvej, bygning 307, 2800 Kongens Lyngby, Denmark; ‡ Scientific Instrument Femtosecond X-ray Experiments, European XFEL GmbH, Holzkoppel 4, 22869 Schenefeld, Germany; ¶ Department of Chemistry, Technical University of Denmark, Kemitorvet, bygning 207, 2800 Kongens Lyngby, Denmark; § Institute of Theoretical Chemistry, Faculty of Chemistry, University of Vienna, Währinger Straße 17, 1090 Vienna, Austria

## Abstract

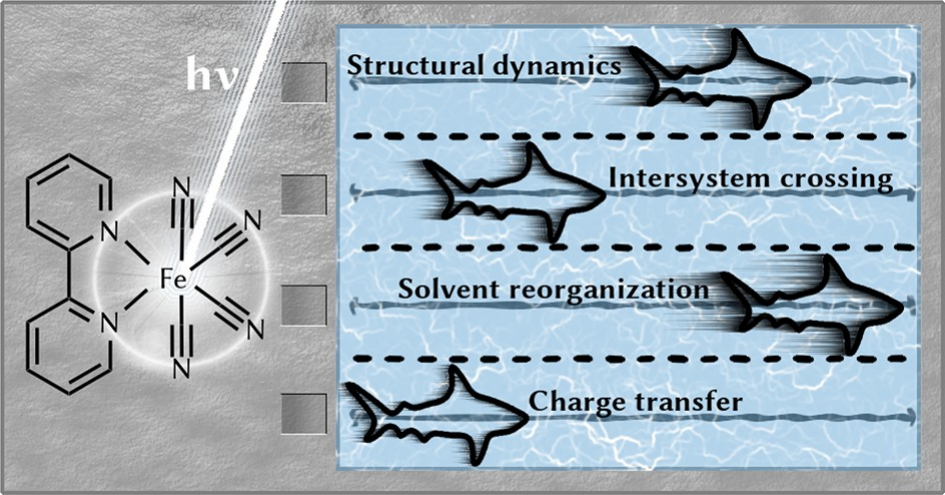

The ultrafast dynamical response of solute–solvent interactions plays a key role in transition metal complexes, where charge transfer states are ubiquitous. Nonetheless, there exist very few excited-state simulations of transition metal complexes in solution. Here, we carry out a nonadiabatic dynamics study of the iron complex [Fe(CN)_4_(bpy)]^2–^ (bpy = 2,2′-bipyridine) in explicit aqueous solution. Implicit solvation models were found inadequate for reproducing the strong solvatochromism in the absorption spectra. Instead, direct solute–solvent interactions, in the form of hydrogen bonds, are responsible for the large observed solvatochromic shift and the general dynamical behavior of the complex in water. The simulations reveal an overall intersystem crossing time scale of 0.21 ± 0.01 ps and a strong reliance of this process on nuclear motion. A charge transfer character analysis shows a branched decay mechanism from the initially excited singlet metal-to-ligand charge transfer (^1^MLCT) states to triplet states of ^3^MLCT and metal-centered (^3^MC) character. We also find that solvent reorganization after excitation is ultrafast, on the order of 50 fs around the cyanides and slower around the bpy ligand. In contrast, the nuclear vibrational dynamics, in the form of Fe–ligand bond changes, takes place on slightly longer time scales. We demonstrate that the surprisingly fast solvent reorganizing should be observable in time-resolved X-ray solution scattering experiments, as simulated signals show strong contributions from the solute–solvent scattering cross term. Altogether, the simulations paint a comprehensive picture of the coupled and concurrent electronic, nuclear, and solvent dynamics and interactions in the first hundreds of femtoseconds after excitation.

## Introduction

Understanding how solvent molecules modulate the photophysical properties of transition metal complexes^1^ has important implications for advancing photocatalysis^2^ and solar energy harvesting,^3^ where such complexes act as catalysts or photosensitizers. Here, a fundamental objective is being able to disentangle how the electronic wave function of the solute, its nuclear positions, and the solvent distribution influence each other as they coevolve on ultrafast time scales after a photon absorption. In this way, processes relevant for catalysts and photosensitizers—such as intersystem crossing (ISC) or charge transfer (CT)—can be better understood, for example in chromophores based on inexpensive and abundant 3d metals, such as iron, which have received considerable attention as sustainable photosensitizers.^4−9^ Differently from 4d or 5d metal complexes, it is challenging to find Fe complexes with sufficiently long-lived metal-to-ligand charge transfer (MLCT) states to enable efficient subsequent charge injection.^10^ For example, the archetypal Ru-based polypyridyl compound, [Ru(bpy)_3_]^2+^ (bpy = 2,2′-bipyridine), shows MLCT lifetimes up to microseconds depending on the solvent.^11^ In contrast, the initially populated MLCT states of the [Fe(bpy)_3_]^2+^ analogue are quenched to lower-lying, metal-centered (MC) excited states within less than 100 fs.^12,13^ Strategies to increase the ligand field splitting—thus destabilizing the MC states relative to the MLCT ones—utilize ligands of large σ-donor strength in combination with strong π-acceptance, such as cyanide, carbonyl, or carbene ligands.^8^ Experiments^6,14,15^ and calculations^16,17^ on iron–carbene systems evidence picosecond and even nanosecond^18^ long-lived MLCT states.

It is well known that the solvent also has a strong effect on the energies of MLCT and MC states and strongly affects the visible absorption spectrum of Fe-cyano-polypyridyl complexes^19−22^ (and Ru analogues^23,24^). This effect is ascribed to donor–acceptor interactions between the cyanide ligands and the solvent molecules,^20^ with a strength that depends on the solvent acceptor number:^25^ a combined measure of the solvent polarity or polarizability and hydrogen bond donor ability.^26^ Recent X-ray absorption spectroscopy and simulations of the L_3_-edge absorption spectra^27^ found that the loss of iron 3d electronic charge during the MLCT transition is compensated by the σ-donation ability of the cyanides, allowing the metal center to preserve the initial metal charge density. Computational insight into how the solvent influences the electronic states of such complexes is often limited to studies employing stationary electronic structure calculations,^16,28,29^ although excited state dynamics simulations are needed to obtain a full temporal picture. Unfortunately, due to their very high cost, such simulations on transition metal complexes are scarce^30^ and the few existing nonadiabatic studies that include explicit solution^31−33^ have focused only on the dynamics of the solute.

In this work, we investigate the excited-state dynamics of [Fe(CN)_4_(bpy)]^2–^ in aqueous solution. With its strong ligand field^34^ and high charge, this complex is a good model to scrutinize specific solute–solvent interactions. Photoexcitation at the lowest-energy band populates MLCT states that decay on ultrafast time scales (≤200 fs) in water^35,36^ but significantly slower (19 ± 2 ps,^37^ 17 ± 2 ps,^27^ or 16.5 ps^36^) in dimethyl sulfoxide (DMSO), according to optical and X-ray spectroscopy experiments. Recent soft X-ray absorption experiments^38^ reported a linear increase of the total L_2,3_ absorption cross section as a function of solvent acceptor number, an effect hypothesized to arise from the solvent affecting metal–ligand bond covalency. That study was supplemented by simulated X-ray and UV–vis steady-state spectra in water, ethanol, and DMSO obtained via ground-state molecular dynamics (MD) computations. However, excited-state dynamics have never been reported for this or similar complexes. Our study employs trajectory surface hopping simulations within a hybrid quantum mechanics/molecular mechanics (QM/MM) framework to reveal the interplay between the evolution of the electronic degrees of freedom, structural changes within the metal complex, and the dynamics of the surrounding solvent. Besides obtaining time-dependent electronic populations for singlet and triplet states, the evolution of charge transfer, and time-resolved structural changes of the solute, we analyze the time-dependent radial distribution functions (RDFs) of the solvent, thus resolving direct solute–solvent interactions and how the solvent reorganizes. We find that there is an immediate solvent response, within 50 fs and thus—counterintuitively—faster than the electronic and nuclear relaxation of the solute. Furthermore, computed time-dependent difference X-ray solution scattering (XSS) signals^39^ predict coherent oscillations in the solute as well as a strong solvent response that should be observable experimentally.

## Experimental Section

### Electronic Structure Calculations

Equilibrium geometries of the ground state and the lowest ^3^MLCT and ^3^MC states of [Fe(CN)_4_(bpy)]^2–^ were optimized in implicit water, acetonitrile (ACN), and DMSO solution using Gaussian 16^40^ and the IEFPCM formalism.^41,42^ We employed a slightly modified B3LYP* functional^43^ (B3LYP with VWN5 local correlation, 15% Hartree–Fock exchange, and 85% GGA exchange; see Section S1.1 of the Supporting Information (SI) for details), the basis sets def2-TZVP^44^ for Fe and def2-SVP^44^ for other atoms, and the D3 dispersion correction.^45^ The triplet states were described with the Tamm–Dancoff approximation (TDA).^46^

Absorption spectra of [Fe(CN)_4_(bpy)]^2–^ in water, ACN, and DMSO were computed from the lowest-lying 30 singlet (*S*_1_–*S*_30_) and 30 triplet (*T*_1_–*T*_30_) states of the corresponding optimized ground-state geometry. These calculations employed the TDA time-dependent version of the same B3LYP* functional in combination with the ZORA scalar relativistic Hamiltonian, the ZORA-def2-TZVP^44,47^ (for Fe) and ZORA-def2-SVP^44,47^ basis sets, and the C-PCM implicit solvation model, as implemented in ORCA 4.1.^48,49^

### Ground- and Excited-State Dynamics Simulations

Initial conditions for the excited-state dynamics simulations of [Fe(CN)_4_(bpy)]^2–^ in water were obtained from MD and QM/MM simulations, described in detail in Section S1.2. First, the complex was solvated in a 25 Å truncated octahedron water box containing two Na^+^ ions. The simulation box was minimized, then heated to 300 K over 20 ps (NVT ensemble), equilibrated to 1 bar over 500 ps (NPT ensemble), and further propagated for 40 ns using a classical force field in AMBER 16.^50^ From the production trajectory, we sampled 500 snapshots, which were locally reheated to 600 K and propagated for a randomized time between 150 and 200 fs in the ground state using electrostatic QM/MM, as explained in ref (51), with the Fe complex in the QM region and water and Na^+^ in the MM region (see Figure S2).

The end points of these short ground-state QM/MM trajectories provide the initial coordinates and velocities for the calculation of an absorption spectrum in explicit water as well as for the QM/MM excited-state trajectories. For the spectrum, the lowest 20 singlet and 20 triplet excited state energies and oscillator strengths were convolved with a Gaussian with a 0.1 eV full width at half-maximum. For the trajectories, the initially excited electronic states were selected through a stochastic algorithm.^52^ As the first absorption band of the simulated spectrum is red-shifted by about 0.2 eV compared to experiment (∼500 nm/2.5 eV),^35,36^ our excitation is centered at 2.35 eV. To increase the number of excited trajectories, we use an excitation window of 2.35 ± 0.10 eV (506–551 nm), resulting in 116 of the 500 geometries being instantaneously excited into the *S*_3_ state, which is the only bright state within the first absorption band, as discussed below.

Ninety-nine initial conditions were then propagated using the surface hopping including arbitrary couplings (SHARC) approach^53,54^ for 700 fs using a nuclear time step of 0.5 fs. The electronic wave functions were propagated with the local diabatization method^55^ and the three-step propagator of SHARC^53^ using a time step of 0.02 fs. The QM energies, gradients, and spin–orbit couplings (SOCs) at each nuclear time step are obtained at the TD-B3LYP* level in ORCA 4.1, as described above, including six singlets and seven triplets, i.e., 27 (6 + 3 × 7) states. Because for [Fe(CN)_4_(bpy)]^2–^—unlike for other Fe complexes^56^—no quintet ^5^MC states have been observed,^35^ no quintet states are considered. Nonadiabatic couplings are calculated through wave function overlaps.^57^ During surface hops, total energy conservation was achieved by rescaling the velocity vector of the solute atoms. An energy-based decoherence correction^58^ was applied to the spin-mixed states. Unlike the classical MD simulations, all the QM/MM simulations (150–200 fs ground state plus 700 fs excited state) were run without periodic boundary conditions or a thermostat, which is reasonable given the rather short simulation time and the large solvent box. Further computational details are in Sections S1.2 to S1.5. Methods employed to analyze the trajectories, e.g., to obtain electronic populations, CT character, RDFs, or XSS signals, are described in Section S2.

## Results

### Absorption Spectrum

**Figure 1:**
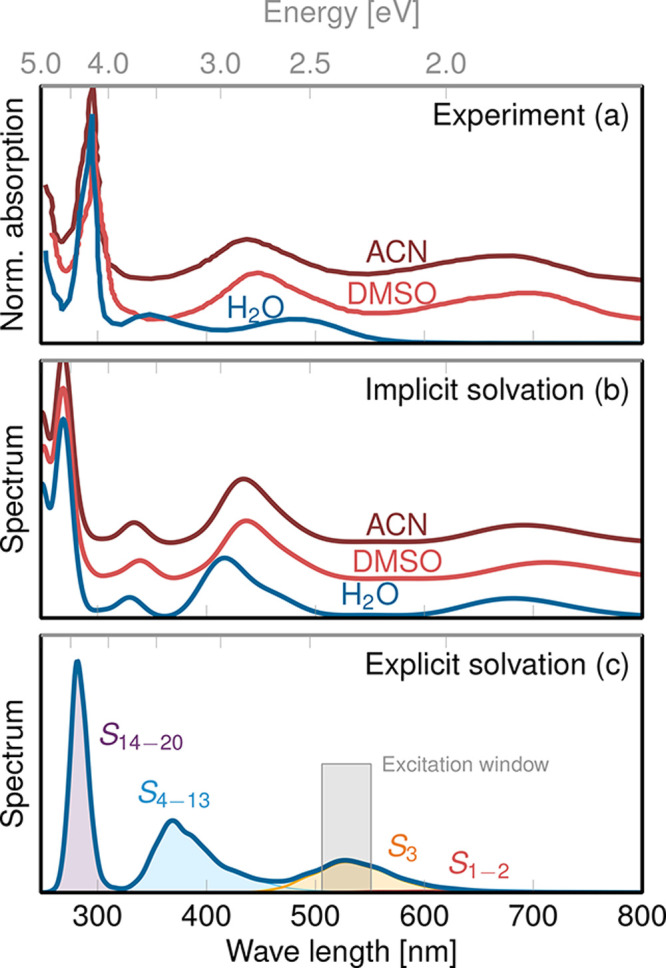
UV/vis absorption spectra of [Fe(CN)_4_(bpy)]^2–^ in solution. (a) Measured spectra recorded in water (H_2_O), dimethyl sulfoxide (DMSO), and acetonitrile (ACN), extracted from ref (35) (vertically offset for clarity). (b) Simulated spectra using implicit H_2_O, DMSO, and ACN solvation (C-PCM), computed at the TDA-B3LYP* level of theory (vertically offset for clarity). (c) Simulated spectrum using explicit water, computed at the TDA-B3LYP*/MM level of theory with a box of 5412 water molecules. The spectrum is decomposed in terms of the contributing electronic states as indicated. The excitation window used to initiate the excited-state dynamics is marked with a gray box.

Figure 1 presents the UV–vis absorption spectra of [Fe(CN)_4_(bpy)]^2–^ in water, DMSO, and ACN. The reported measured spectra^35,36^ (Figure 1a) evidence a very strong solvent-dependent shift of the first absorption band, from around 700 nm in ACN and DMSO to 490 nm in water. The spectra calculated using implicit solvent (Figure 1b) are almost identical for the three solvents and so are the state characters of the excitations (Section S3.1). Yet, while a very good agreement between experiment and implicit solvation simulation is obtained for DMSO and ACN, the strong solvatochromic shift in water is not reproduced. An appreciable shift of the first absorption band is obtained only upon including explicit water molecules (Figure 1c) via QM/MM. Now this band is shifted only by 45 nm (0.2 eV) relative to the measured spectrum, in contrast to the 200 nm (0.6 eV) shift obtained with implicit water. Furthermore, we obtain a good overall match of the shape of the spectrum, with three separated and approximately equispaced absorption bands with increasing intensity at increasing energy, in line with the experimental profile. We thus conclude that the strong solvatochromism observed for [Fe(CN)_4_(bpy)]^2–^ in water cannot be explained solely by solvent polarity, but other effects that require an explicit water treatment, such as hydrogen bonding, are relevant.

The decomposition of the spectrum in terms of electronic states indicates that the first absorption band is solely due to the bright *S*_3_ state (orange area in Figure 1c), which is thus chosen as a target of the excitation. From optical spectroscopy measurements, this band has been attributed to states of MLCT character.^34^ Here, we use a fragment-based charge transfer analysis^59^ to classify the electronic states (see Section S3.2). We choose to divide the molecule into two fragments, Fe(CN)_4_ and bpy, because the metal and the cyanides act as a chromophoric unit, analogous to other metal complexes with tightly bound π-acceptor ligands^32^ and confirmed by a correlation analysis.^59^ With the labels M = Fe(CN)_4_ and L = bpy, MC, MLCT, LMCT (ligand-to-metal CT), and LC (ligand centered) states are possible. The CT analysis of the absorption spectrum (see Figure S5) shows that the first band indeed arises from transitions with more than 80% MLCT character and less than 10% MC character. The second band around 380 nm is due to states with at least 50% MLCT and less than 20% MC character. Finally, the third band below 300 nm has less than 50% MLCT character, virtually no MC character, but instead states with LC character (i.e., bipyridine ππ*).

### Excited-State Electronic Relaxation Dynamics

**Figure 2:**
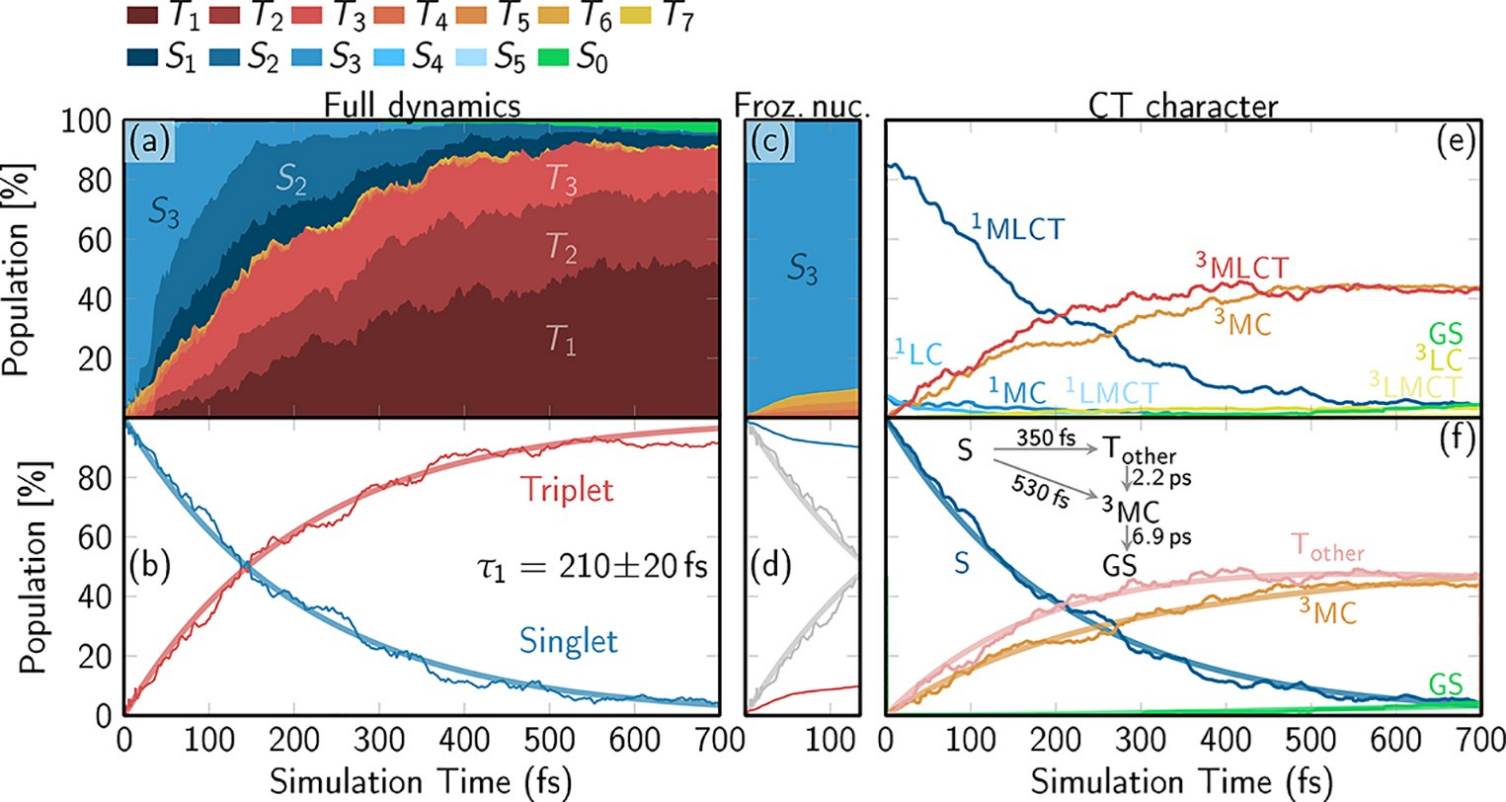
QM/MM-SHARC time-dependent electronic populations. (a, c) Contributions from each excited state as a stacked area plot from the fully relaxed dynamics (a) or with frozen nuclei (c). (b, d) Total singlet and triplet populations (thin lines) with monoexponential fits (thick lines) and extracted time constants with errors estimated from bootstrapping from the fully relaxed dynamics (b) or with frozen nuclei (d). Thin lines in (d) are the actual singlet (blue) and triplet (red) populations of the frozen-nuclei simulations, and gray lines are the populations from (b). (e) Time-dependent populations classified in terms of individual charge transfer and multiplicity contributions. (f) Time-dependent populations (thin lines) of the ground state (GS), all singlet states (S), the ^3^MC state, and remaining triplet states (T_other_). Thick lines depict the results from a global fit that employs the kinetic model shown in the inset.

Figure 2a shows the time evolution of spin-free electronic populations (see Section S2.2 for details) colored according to their multiplicity. The vertical width of each band represents the fraction of the population at a particular time. Initially, the population is only in the *S*_3_ excited state (light blue), but within the first 100 fs it undergoes internal conversion to lower-lying singlet states (*S*_2_, *S*_1_), concomitant with intersystem crossing to the triplet manifold (mainly to *T*_3_, *T*_2_, *T*_1_). By the end of the simulated 700 fs, more than 90% of the population is in the triplet states, with about 50% in the *T*_1_ state. Only a few trajectories relax to the ground state (*S*_0_) within the simulated time frame. In Figure 2b, all singlet and all triplet populations are summed, and a monoexponential kinetic fit to the singlet decay gives an overall ISC time of 0.21 ± 0.02 ps. Inspired by some of our previous studies,^32,60^ we also carried out frozen-nuclei dynamics, i.e., where the nuclear motion is turned off. As Figure 2c,d illustrate, ISC is significantly slower (>0.7 ps) and incomplete (saturating at only about 10% triplet). This suggests that the change of spin in [Fe(CN)_4_(bpy)]^2–^ is strongly coupled to its nuclear dynamics, as observed for other Fe-based complexes.^9^

To observe how the CT character changes along the dynamics, we classify^59^ the singlet and triplet states according to the four contributions mentioned above (Figure 2e). The main protagonists are the ^1^MLCT, ^3^MLCT, and ^3^MC states, which together encompass more than 90% of the population at any given time step. Immediately after excitation, the population shows predominant (85%) MLCT character, as assigned in previous transient absorption experiments.^35,36^ From the ^1^MLCT state, the system evolves into a mixture of ^3^MLCT and ^3^MC of roughly equal contributions by the end of the 700 fs. A more detailed analysis (Section S3.3) of the charge transfer between Fe, (CN)_4_, and bpy (i.e., in terms of three fragments) confirms that Fe and (CN)_4_ effectively act as a single unit.

To perform a kinetic analysis, we differentiate four states or a set of those: (1) the ground state (GS), (2) all the excited singlet states (the dominant ^1^MLCT plus ^1^MC and other negligible contributions) denoted as “S”, (3) the ^3^MC state, and (4) the remaining triplet states (the dominant ^3^MLCT plus other negligible contributions) denoted as “T_other_”. The populations of these four sets are fitted using the kinetic model shown in the inset of Figure 2f. Accordingly, we find a branched decay pathway from the singlet states into the triplet states of mainly ^3^MLCT or ^3^MC character, with time constants of 0.35 ± 0.04 ps and 0.53 ± 0.09 ps, respectively. The 2.2 ± 1.5 ps time constant in the kinetic model shows that the ^3^MC population will continue growing, indicating that the ^3^MC minimum is slightly lower in energy than the ^3^MLCT minima. Adding up all states of MLCT character and performing a monoexponential fit gives an MLCT time scale of 0.89 ± 0.10 ps, which is somewhat slower than the 0.1–0.2 ps time scale reported experimentally.^35,36^ We note, however, that time constants from computed populations and from experimental observables do not necessarily need to agree, as they do not represent the same observable.^32,61^ We also observe a slow decay to the ground state with a 6.9_–5_^+13^ ps time constant (with very large uncertainty due to the very small number of trajectories that decayed), roughly in line with the experimental 13 ps lifetime reported for this state in water.^35^

### Structural Dynamics of the Solute

**Figure 3:**
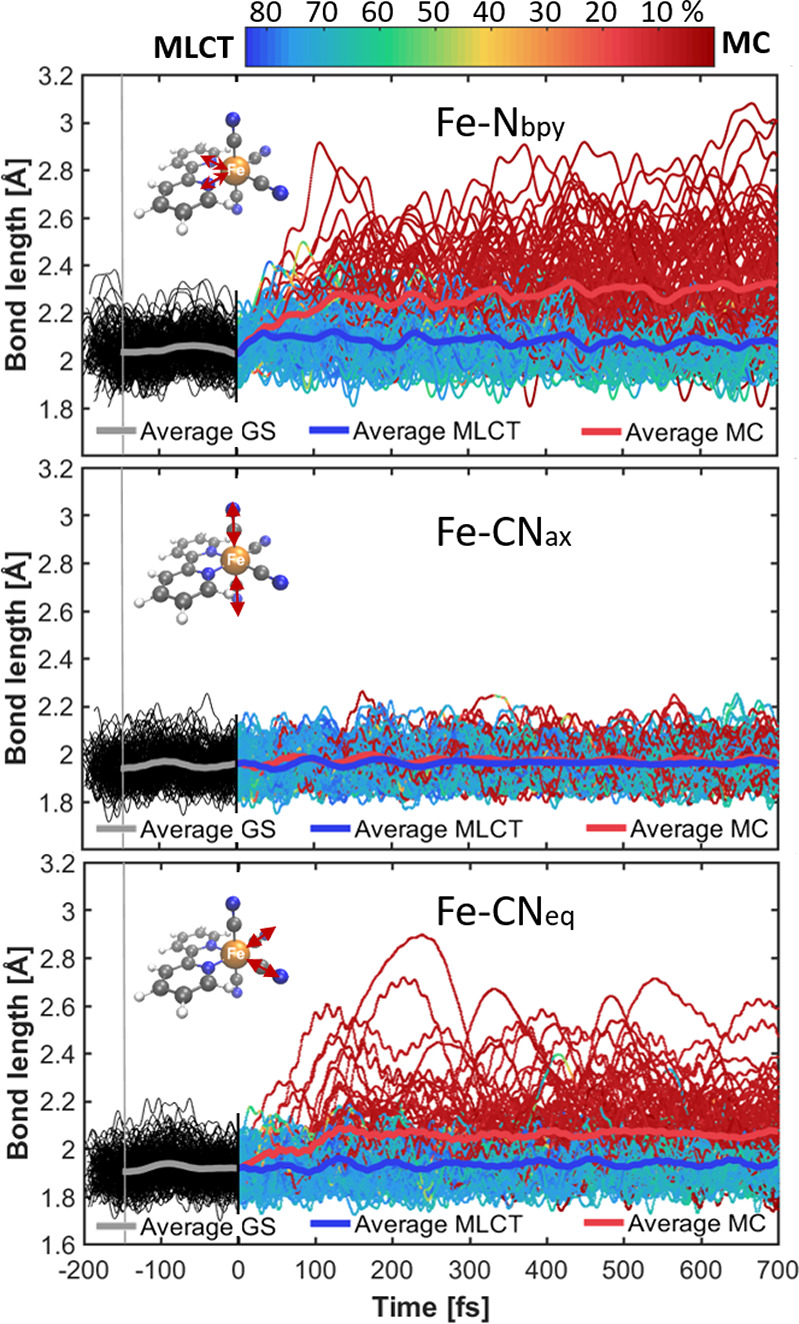
Time-dependent Fe–ligand bond lengths (indicated by arrows in the molecular structures) from each of the QM/MM-SHARC trajectories in the ground (black) and excited states (MLCT in blue or MC in red). Thick lines are weighted averaged bond lengths for the swarm of trajectories. The vertical thin gray line at −150 fs indicates the earliest time where the full statistics of all ground-state QM/MM trajectories is available (due to the randomized 150–200 fs simulation time in *S*_0_).

Previous X-ray-based experimental studies on Fe-based complexes reported that the dynamics of MLCT and MC states heavily involves the Fe–ligand bonds as main reaction coordinates.^62^ Thus, Figure 3 displays the evolution of the Fe–N bonds to the bipyridine ligand (Fe–N_bpy_), the Fe–C bonds to the axial cyanides (Fe–CN_ax_), and those to the equatorial cyanides (Fe–CN_eq_). Blue or red indicates that the current active state is of predominant MLCT or MC character, respectively. The data at negative times (black) show the bond lengths propagated in the electronic ground state.

Upon excitation, some bond lengths are significantly perturbed, particularly those from trajectories switching to MC character (average Fe–N bond lengths change by about 0.2 Å, equatorial Fe–C bond lengths by about 0.1 Å). In contrast, trajectories in the MLCT states undergo minor changes (<0.04 Å). The axial Fe–C bonds do not show any average elongation in the trajectories, regardless of their character. Also noticeable is that while the widths of the bond length distributions for the MLCT states are similar to those of the ground state, trajectories in the MC states exhibit significantly wider distributions, a sign for the population of a vibrationally hot MC state, which would comply with the interpretations given in ref (35) and ref (63). Averages of additional bond lengths depending on state character from the dynamics are found in Section S3.4, showing that the C–N bond lengths of the cyanide ligands marginally shorten for MLCT (<0.01 Å) and MC (0.01–0.03 Å) states and that the MLCT states induce notable changes in the bpy bond lengths. In passing, we note that the averages obtained from the QM/MM-SHARC dynamics are significantly different from bond lengths obtained from optimizations in implicit water, which in turn are incorrectly predicted to be the same for different solvents (Table S5).

### Structural Dynamics of the Solvent

**Figure 4:**
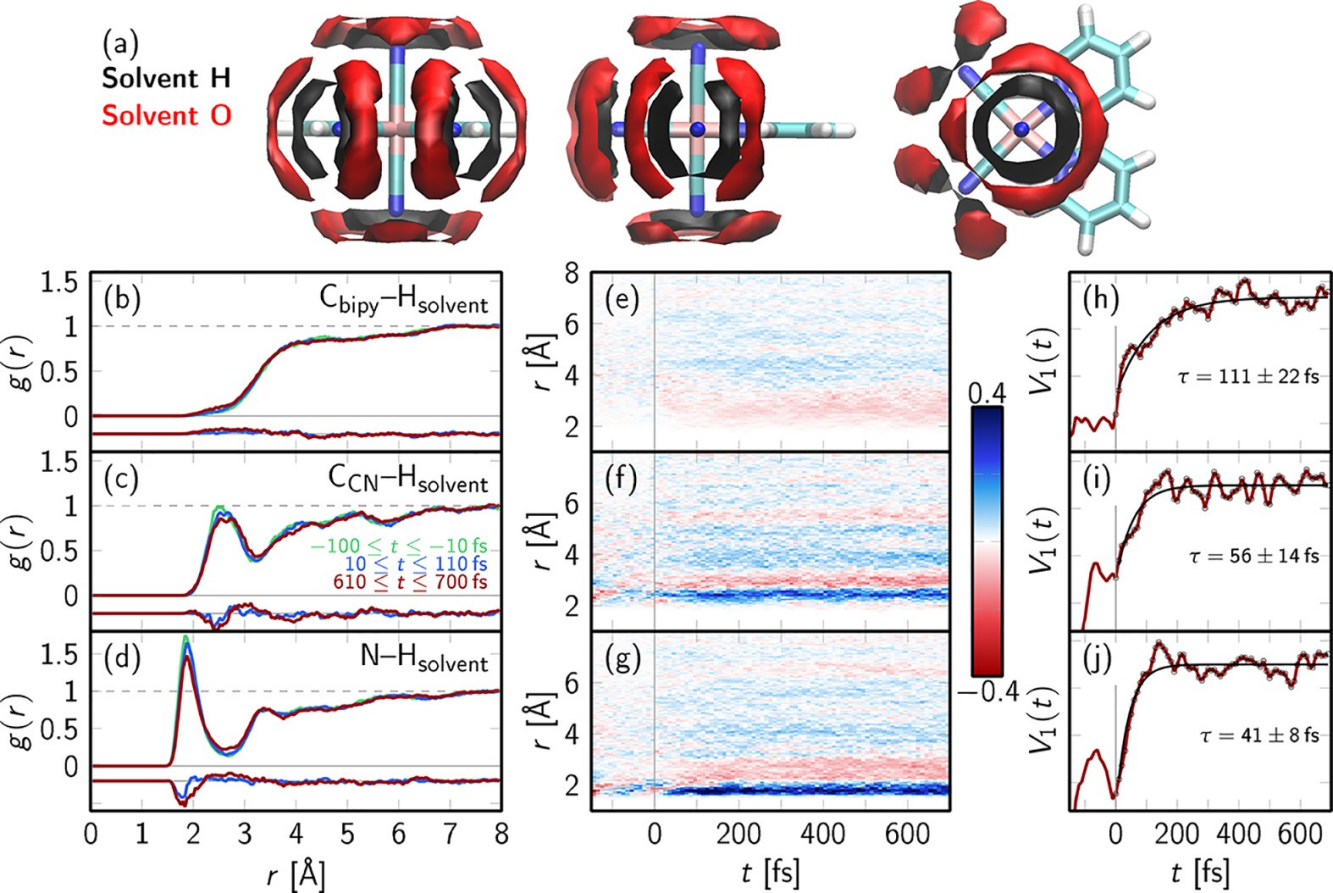
Solvent distribution and dynamics. (a) Initial 3D spatial solvent distributions, showing regions where the density is at least three times the average particle density. RDFs of (b, e, h) the cyanide C atoms (C_CN_), (c, f, i) the bpy C atoms (C_bpy_), and (d, g, j) the N atoms relative to the solvent H atoms (H_solvent_). (b–d) Averaged RDFs for selected time spans: ground-state dynamics (green), early excited-state dynamics (blue), and late excited-state dynamics (red). Offset by −0.2 is the difference relative to the averaged ground state. (e–g) Difference RDFs for each time step relative to the average ground state. (h–j) First temporal components *V*_1_(*t*) (red) according to SVD analysis of the difference RDFs and kinetic fits (black) of the signal at *t* > 0 (circles).

The initial solvent distribution around the molecule before excitation is depicted by isosurfaces in Figure 4a. Each cyanide is coordinated by up to four water molecules arranged on a ring, attacking in a “side-on” fashion and evidencing the directional hydrogen bonds formed. The response of that solvent distribution to the excitation can be analyzed by means of RDFs. The temporal evolution of the solvent distribution is presented in Figure 4b,c as average solute–solvent RDFs of the carbon atoms from the bipyridine ligand (C_bpy_) and the cyanides (C_CN_) relative to the solvent hydrogen atoms (H_solvent_). Figure 4d shows the RDFs of all nitrogen atoms relative to the solvent hydrogen atoms. Additional RDFs are found in Section S3.5.

The RDFs are averaged over selected time ranges: in the ground state (−100 ≤ *t* ≤ −10 fs, green), at early relaxation times (10 ≤ *t* ≤ 110 fs, blue), and at late times (610 ≤ *t* ≤ 700 fs, red). The differences relative to the averaged ground state are shown at the bottom of Figure 4b–d (offset by −0.2). Figure 4e–g show difference RDFs for each time step relative to the average ground state. Since these differences are relatively small on these short time scales, we effectively separate the main trends from noise using a singular value decomposition (SVD). This decomposition can be written for a given difference RDF as Δ*g*(*r*, *t*) = *∑*_*i*_*U*_*i*_(*r*)·*s*_*i*_·*V*_*i*_(*t*), where the functions *U*_*i*_(*r*) are the left singular vectors describing the spatial dependence of the *i*th contribution and *V*_*i*_(*t*) are the right singular vectors that describe the temporal evolution of the *i*th component. The scalar numbers *s*_*i*_ are the singular values that provide the relative importance of the *i*th contribution in descending order. As in all three examples, *s*_1_ is significantly larger than all other *s*_*i*_ (Section S3.6), we only consider the first component here (*V*_1_(*t*)), shown in Figure 4h–j. These temporal profiles were fitted with monoexponential functions that provide a time scale for the solvent reorganization dynamics upon photoexcitation. Additional SVD components and fits are given in Section S3.6.

From the perspective of the carbon atoms of the complex, a very different solvation organization around the cyanides and bipyridine ligands is found. The bipyridine ligand (Figure 4b) shows unstructured solvent coordination, indicating only minor directed interactions with the nearest solvent and, furthermore, relatively small structural changes from the averaged ground (green) to excited states (blue and red). In contrast, the cyanides (Figure 4c) exhibit a stronger interaction with the nearest solvent, as observed from the sharp peak located at roughly 2.5 Å. This feature is even more prominent in the N–H_solvent_ RDFs (Figure 4d), showing a strong peak around 2 Å. Interestingly, the strong interaction between the cyanide ligands (Figure 4c and d) and the nearest water molecules in the ground state is rapidly and significantly weakened upon photoexcitation, as observed around 2–2.5 Å from the decrease in peak height and shift toward longer ligand–solvent distances upon excitation. An analysis of the solvation structure around the cyanides in a charge-transfer-weighted fashion reveals slightly different RDFs (N–H_solvent_ and N–O_solvent_) for MLCT and MC states (Section S3.7). It is found that the MLCT state induces a slightly stronger solvent response, with a larger decrease of the N–H RDF peak below 2 Å and larger increase at 2–3 Å compared to the MC state (Figure S11a).

The time-resolved difference RDFs (Figure 4e–g) show that the response of the solvent–solute structure around the cyanides upon excitation is dominated by a shift of the first solvation shell toward longer distances; see the reduction around 2 Å (blue) and the increase around 2.5 Å (red). Figure 4h–j evidence that the dominating contribution to this shift occurs on very fast time scales. For the solvent distribution around the bipyridine ligand (Figures 4e,h) the response time is approximately 100 fs, although the changes are only very small. However, around the cyanide ligands (Figure 4i,j) the solvent relaxation is 40–50 fs.

The corresponding RDFs using the solvent oxygen atoms (Section S3.5) give consistent time scales of 55 fs for the rearrangement around the cyanides. As this time scale is different from the change in Fe–cyanide bond lengths in Figure 3 (100 fs time scale), we assume that the fast changes in solvation are independent of the solute bond length dynamics.

An analysis of the differences between equatorial and axial cyanides in terms of N–H and N–O RDFs (Section S3.8) reveals that the axial cyanides attract slightly more hydrogen bonds (sharper peaks at ∼2 and ∼3 Å, respectively) than the equatorial cyanides. However, these stronger axial hydrogen bonds are already found in the ground state. Even though the axial hydrogen bonds start out stronger than the equatorial ones, both ligands show comparable solvent relaxation time scales of 40–60 fs.

As documented in Sections S3.9 and 3.10, we also investigated the solvent dynamics around the cyanides in terms of hydrogen bond counting using traditional distance/angle criteria and in terms of angular-resolved RDFs (ARDFs). The hydrogen bond counting shows that after excitation the number of hydrogen bonds around the cyanides decreases from about 3.8 to 3.3 per cyanide (Figure S13). However, the time scale of this decrease is hard to quantify, as extracted monoexponential decay times depend strongly on the chosen distance/angle criteria, varying between 40 and 130 fs. Hence, it appears that the solvent rearrangement is better described by the SVD analysis of the RDFs. The ARDFs (Section S3.10) seem to indicate that solvent relaxation includes water moving slightly away from the cyanides, but also turning the H atom a bit away from the cyanide N atoms. Unfortunately, the noise level in the ARDFs is too large to follow the solvent dynamics in more detail.

### Time-Resolved X-ray Scattering Difference Signals

**Figure 5:**
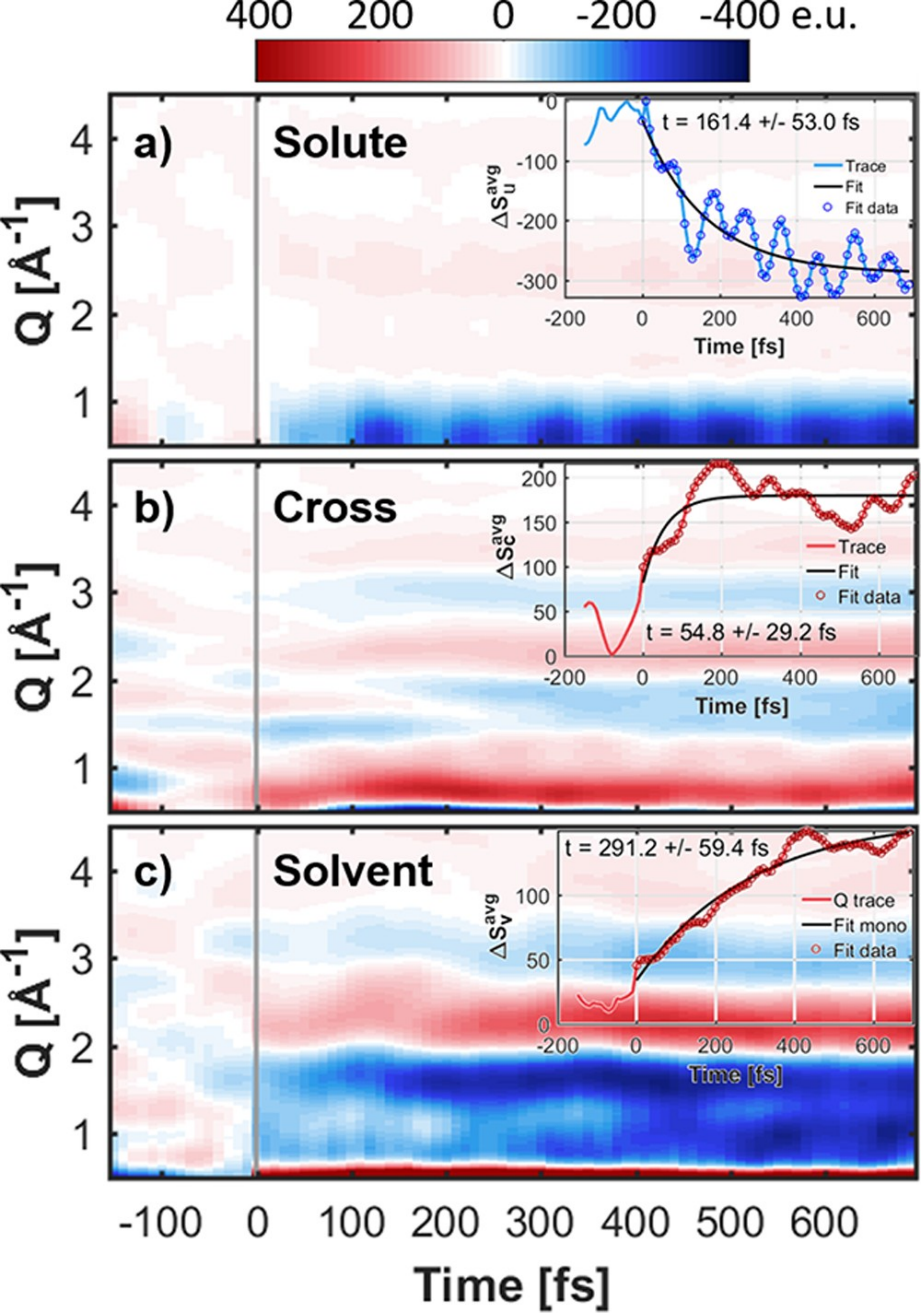
Calculated time-dependent difference scattering signals, Δ*S*(*Q*, *t*), as a function of the scattering vector *Q* and time *t* after excitation. The calculated difference scattering signal is separated into solute (top), solute–solvent cross-term (middle), and solvent (bottom) contributions. The insets show traces of the average signal around the low-*Q* peaks (solute: 0.5 < *Q* < 0.7 Å^–1^, cross: 0.6 < *Q* < 1.0 Å^–1^) or a larger range for the solvent (0.7 < *Q* < 4.0 Å^–1^) along with monoexponential fits. See Section S3.11 for details on the normalization of the different contributions.

In order to connect the results with an experimental observable directly sensitive to structure, we also calculated the time-resolved XSS difference signals (Section S2.6). Using the procedure of Dohn et al.,^39^ the total difference scattering signal Δ*S*(*Q*, *t*) = *S*(*Q*, *t*) – *S*_average ground_(*Q*) is partitioned into three components arising from changes in the solute structure, in the solvent structure, and in the solute–solvent cross-term interactions, which generally evolve on several different time scales. Figure 5 shows the calculated difference scattering signals Δ*S*(*Q*, *t*) as a function of the scattering vector *Q* and time *t*. A one-dimensional representation of the same data is given in Section S3.11 to facilitate comparison of the three contributions.

The solute contributions (Figure 5a) evidence a strong negative feature (dark blue) for *Q* ≤ 1 Å^–1^, consistent with a local decrease in electron density near the metal center, as a result of metal–ligand bond elongations, in agreement with experimental observations for similar metal complexes in MC excited states.^15,63,64^ We also observed metal–ligand bond elongations mainly associated with the population of MC excited states. A monoexponential fit of the difference signal in the 0.5 < *Q* < 0.7 Å^–1^ region (see inset) gives a time constant of about 160 fs.

**Figure 6:**
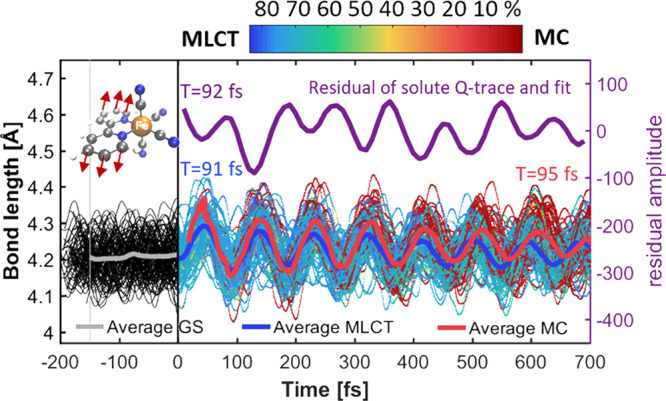
Temporal evolution of the ring–ring stretch mode of the bipyridine ligand (illustrated in the upper left corner), calculated as the average of the distances between equivalent C/N atoms of the two pyridine units. Color indicates charge transfer character. Thick lines show the average of bonds related to predominant MLCT (blue) or MC (red) character. The plot above (in purple) shows the residual of the monoexponential fit from the inset of Figure 5 (top), to enable comparison of the similar oscillation periods. Periods (*T*) of the oscillations are calculated via Fourier transforms of the averages.

On top of the monoexponential decay we observe strong oscillations, which have a period of about 92 fs according to a Fourier transformation of the fit residual. In order to investigate the origin of this coherent 92 fs oscillation, we analyzed the vibrational modes within the excited-state dynamics. In the Fourier transformations of the normal mode coordinates obtained from the average trajectory, we found one mode showing a strong and coherent oscillation with a period of about 93 fs, matching the 92 fs period from the residual. Inspection of this mode identified it as the ring–ring stretching mode of the bipyridine. Figure 6 shows this motion in terms of the average of the distances between equivalent C/N atoms of the two pyridine units. From the color coding, we see that the oscillation period does not depend strongly on the character of the state, as the oscillation period is 91 fs (366 cm^–1^) for MLCT states and 95 fs (351 cm^–1^) for MC states. The slight decrease in oscillation frequency from MLCT to MC state is consistent with the results of the frequency calculations in implicit solvation (Section S3.12), which give 88 fs (378 cm^–1^) for the MLCT state and 96 fs (349 cm^–1^) for the MC state. Comparison of these oscillation periods with the bond length oscillations of the Fe–C and Fe–N bond lengths in Figure 3 (about 85 fs) also shows that the bpy ring–ring stretch motion mixes with the Fe–N stretch. It is probably due to this mixing that this mode contributes so strongly to the difference scattering signals, as the Fe atom is the strongest scatterer.

We turn next to the solute–solvent and solvent–solvent contributions to the difference XSS signal (Figure 5b and c). Here, discernible patterns throughout the simulated time frame arise from changes in the solvent structure in the vicinity of the excited solute, highlighting the sensitivity of XSS to the structural dynamics in the largely disordered solvent. In particular, shape differences between the signals below *t* < 200 fs and at longer time scales indicate an ultrafast reorganization of the solvent. We observe a strong positive feature (red area) around 0.6 < *Q* < 1.0 Å^–1^, directly upon excitation. This early feature might arise from the previously mentioned weakening and reorientation of the H-bonds between the cyanides and the nearest water molecules and the concomitant increase of solute–solvent distances. The time evolution of the early feature is characterized by the monoexponential fit of the average cross-term signal around the strong positive peak (0.6 < *Q* < 1.0 Å^–1^) shown in the inset of Figure 5b that provides a time constant of the cross term of about 55 fs. This is in very good agreement with the 40–55 fs time constants obtained from the analysis of the C_CN_/N_CN_–O_solvent_/H_solvent_ difference RDFs (Figure 4 and Section S3.5). On the contrary, the 55 fs time constant from the cross term does not match the RDF time constants for the bipyridine, evidencing that the difference scattering signals are primarily due to rearrangement of the solvent around the cyanide ligands.

The cross-term contribution also shows a feature around 1.5 < *Q* < 3.5 Å^–1^ (negative peak 1.8 Å^–1^, positive peak 2.3 Å^–1^, negative peak 3.0 Å^–1^) that appears more slowly than the above-mentioned strong positive peak at low *Q*. A monoexponential fit to the average absolute cross-term signal (not shown) in this range gives a time constant of 0.62 ± 0.16 ps. This characteristic feature is most likely the solvent heat response due to transfer of energy from the solute to the solvent, as the heat difference scattering signal of water observed in experiments shows such a feature in this *Q* range and typically appears within a few hundred fs to a few ps.^63,65,66^

The solvent contribution in Figure 5c shows strong features over a large *Q* range (0.7 < *Q* < 4.0 Å^–1^). The strong features show a direct response of the solvent upon excitation, indicating a significant rearrangement in the solvent, expected to be observable experimentally. The overall time scale (inset plot) of the solvent rearrangement is about 0.3 ps, as extracted from a monoexponential fit of the average absolute signal over the large *Q* range. Thus, as recently demonstrated by Biasin et al.^67^ for a bimetallic CN-bridged Fe–Ru complex, an ultrafast time-resolved XSS experiment would be expected to be sensitive to both the structural changes in the solute, i.e., the Fe–ligand bond elongations plus the motions within the bipyridine ligand, and the solvent, including the immediate solvent reorganization around the cyanide ligands as well as slower dynamics.

**Figure 7:**
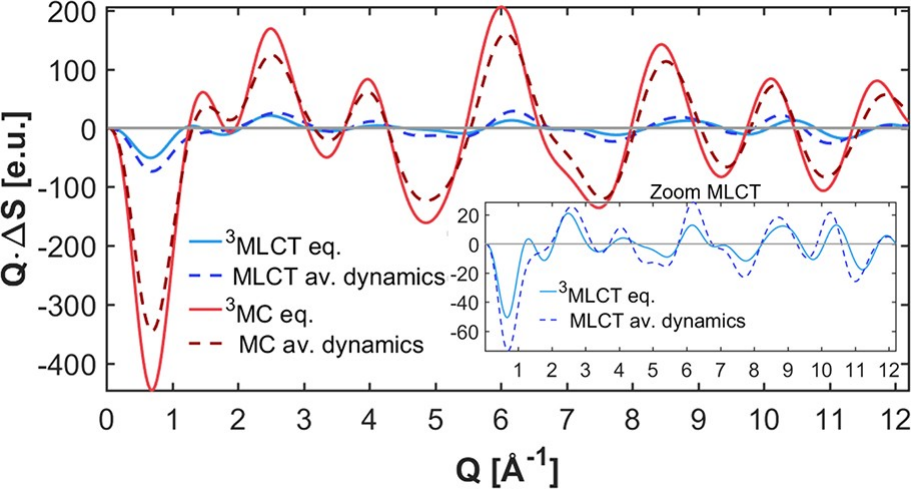
Comparison of solute difference scattering contribution Δ*S*_*u*_ calculated from either CT-weighted average geometries from the 99 SHARC trajectories (av. dynamics) or the optimized geometries (eq.). The *y*-axis is multiplied with *Q* to enhance the trends at larger values of *Q*. The inset shows a zoom-in of the MLCT data.

Oftentimes, the interpretation of time-resolved XSS data relies on optimized structures for the different involved electronic states.^68,69^ However, given the large amount of energy in the complex—after excitation but prior to relaxation (to the lowest excited-state PES) and cooling (transferring energy to the solvent)—it is actually very unlikely that the system is close to any minima on the PESs; therefore, average structures from the QM/MM-SHARC dynamics simulations are more appropriate than simple optimized structures. The difference between these two approaches (optimization versus dynamical average) can be seen in Figure 7 for the solute difference XSS signal. We computed CT-weighted average geometries (dashed) to obtain separate results for the MLCT (blue) and MC states (red), which can be compared to the optimized MLCT and MC structures (solid). The difference signal has much larger magnitude for the MC states than for the MLCT states, due to the larger structural changes compared to the ground state. Furthermore, the averages from the dynamics produce a different magnitude and shape than the optimized structures (for ^3^MLCT the average gives larger magnitudes, for ^3^MC the optimized structure gives larger magnitudes). Hence, basing the interpretation of experimental signals on optimized structures—as often done in the literature^68,69^—could lead to an over- or underestimation of the fraction of excited molecules. Additionally, the differences in shape of the calculated signals between the dynamics averages and optimized structures may influence the experimentally extracted structural response of the solute. Especially for the case of MLCT, we find significant differences in signal shape (see the inset). This illustrates that high-quality dynamics simulations might be needed to precisely extract experimental structural changes as well as excited-state populations and even time scales.

## Discussion

Experimentally,^20,35,36^ [Fe(CN)_4_(bpy)]^2–^ exhibits a strong solvatochromism of the absorption spectra as well as of its photoinduced dynamics. Simulations of the absorption spectra using implicit water (via a solvent continuum model) cannot reproduce this effect, whereas the absorption spectra with implicit ACN or DMSO reproduce the experiments. Simulations using explicit solvent molecules within an electrostatic QM/MM framework provide a much better description of the large shift of absorption in water. For this reason, our nonadiabatic dynamics simulations were carried out with this explicit solvent description.

Excitation at 2.35 ± 0.10 eV (506–551 nm) populates the *S*_3_ state of ^1^MLCT character. The excited singlet population decays via ISC with a time constant of 0.21 ± 0.02 ps to triplet states with ^3^MLCT and ^3^MC character. The superposition of ISC and charge transfer dynamics (from MLCT states to MC states) is well described by a phenomenological model with a branched decay mechanism including ^1^MLCT → ^3^MLCT (0.35 ± 0.04 ps) and ^1^MLCT → ^3^MC (0.53 ± 0.09 ps) processes. The overall ISC time constant is in reasonable agreement with ultrafast decay constants (0.1–0.2 ps) from spectroscopy experiments.^35,36^ However, the experimental time constants were not assigned to ISC (since the experiment does not distinguish between singlet and triplet MLCT states) but to an overall decay of the MLCT states. In contrast to the assigned decays from spectroscopy, our simulations predict a lifetime of the MLCT states of about 0.9 ps. The differences between the experimental and our results could arise from several factors. On one hand, it is possible that our simulations overestimate the ^3^MLCT lifetime due to a slight underestimation of the solvent shift of the MLCT states in the QM/MM calculations (recall Figure 1), producing slightly too low MLCT states relative to the MC states. On the other hand, the experimental decay constants involve several uncertainties in the assignments, since the optical transient absorption data found a slower (∼0.2 ps) MLCT → MC transition than that extracted from X-ray emission spectroscopy (XES) data (0.09 ps) and was under slightly different experimental conditions, i.e., excitation at 500 or 400 nm.^35,36^

A branched decay mechanism was also observed in both experiments^15^ and simulations^16^ of iron–carbene systems, in which initially excited MLCT states bifurcate into long-lived ^3^MLCT and ^3^MC excited states. The experimental results,^15^ employing both time-resolved XES and XSS, show an initial excitation into MLCT states, where spin cannot be assigned, which decays into both ^3^MLCT (60%) and ^3^MC (40%) states with lifetimes in the ps regime of both states. The calculations reported in ref (16), which included both TD-DFT and MM-MD and QM/MM-MD simulations, indicate direct excitation into ^1^MLCT states followed by ps long-lived ^3^MLCT and ^3^MC excited states, with a strong dependence on the inclusion of solvent effects.

One important observation from our simulations is the strong dependence of ISC on the nuclear motion (cf. Figure 2). If nuclei are frozen, the time scale and yield of ISC considerably change, from ∼210 fs and nearly 100% quantum yield with moving nuclei to larger than 700 fs and 10% yield with frozen nuclei (see Figure 2c,d). This is most probably because the energy gaps between the initial *S*_3_ state and the triplet states are too large and nuclear motion is required to tune the singlet–triplet energy gaps that enable ISC. This illustrates one aspect of the strong coupling between electronic and nuclear dynamics in this molecular system. Similar nuclear-driven ISC was found previously in a combined X-ray scattering and emission study^63^ of the [Fe(bpy)_3_]^2+^ complex, which reported that electronic population transfer from ^3^MC to ^5^MC is driven by Fe–ligand bond stretch motion. However, this behavior cannot be generalized to every transition metal complex.^70,71^ For example in [Re(CO)_3_(im)(phen)]^+^ (im = imidazole, phen = 1,10-phenanthroline), excited-state dynamics simulations revealed an immediate ISC process that is essentially independent of nuclear motion.^32^ In particular, an ultrafast (∼8 fs) singlet–triplet equilibration was also observed under frozen nuclei conditions—whereas the subsequent slower (∼420 fs) ISC component was not—thereby evidencing the role of electronic effects. This difference between Re and Fe complexes might be attributed to Re being a 5d metal, with significantly larger SOCs and smaller singlet–triplet gaps than in Fe. However, SOC is not the only parameter to determine ISC rates, as there are reports^70,71^ of Re complexes with slower ISC than in Ru/Fe complexes despite the stronger SOC of the former. It was suggested that the SOCs eventually reach a saturated level and structural effects, in particular Re–ligand modes, as well as the size of singlet–triplet gaps, modulate the ISC rate.

The analysis of the structural changes within the solute shows that the Fe–N bond lengths greatly depend on the predominant charge transfer character of the electronic state. Excited states of mainly MC character are associated with longer (∼0.2 Å) Fe–N bonds relative to the ground state, where the time to adapt to the longer bond length is 100–200 fs. This is in good agreement with observations of Fe–ligand bond elongations related to MC population, on the order of about 0.2 Å as reported for the [Fe(bpy)_3_]^2+^ system^72,73^ and other iron-based complexes.^74−76^ In contrast, the population of excited states of predominantly MLCT character leads to smaller (≤0.04 Å) Fe–N bond elongations or no structural changes relative to the ground state. Density functional theory (DFT) geometry optimizations^77^ of the ruthenium analogue [Ru(bpy)(CN)_4_]^2–^ including 4–8 explicit water molecules evidence Ru–N bond elongations of about 0.05 Å from the ground to the lowest lying triplet state showing ^3^MLCT character, in excellent agreement with our observations. From theoretical work, the reported bond length changes related to population of MLCT excited states in both the [Ru(bpy)_3_]^2+^ system^78−80^ and the [Fe(bpy)_3_]^2+^ system^73,81^ are even smaller (<0.01 Å). Experimental work based on X-ray absorption spectroscopy^82^ on [Ru(bpy)_3_]^2+^ also suggests small structural changes on the order of 0.03 Å. Thus, the structural response of the Fe–bipyridine bonds of tetracyano complexes related to MLCT states appears to be similar or slightly larger than for tris-bipyridine complexes.

One interesting aspect of the solute structural response is the strong difference between the Fe–C bond length dynamics of the equatorial and axial cyanides in the MC state (recall Figure 3). Whereas the axial cyanides stay fully coordinated, the equatorial Fe–C bonds stretch strongly upon population of the MC state, indicating a change of bonding status. This observation can be explained by studying the molecular orbitals of [Fe(CN)_4_(bpy)]^2–^ (Section S3.1). The three lowest-energy ^3^MC states are described by excitations from one of the three t_2g_ orbitals (numbers 79, 80, 81) to orbital 86, which is the lower of the two e_g_ orbitals (86, 87). Orbital 86 is localized on the four equatorial Fe–N/C bonds, and consequently the population of this orbital weakens these bonds and induces the strong structural changes observed in the dynamics.

The structural reorganization of the solvent is best discussed with the RDFs presented in Figure 4b–d. Already in the electronic ground state (green curves) we observe that the nearest solvation structure is different around the cyanides and around the bipyridine ligands, with a stronger interaction in the first case. This difference in solvation structure was also demonstrated by RDFs from classical MD simulations by Jay et al.^38^ Actually, such a ligand-dependent solvation structure was suggested by Toma et al.^20^ back in 1983, where a solvent such as water (high acceptor number) preferentially stabilizes the electronic (metal-centered) ground state of the metal complex by allowing for removal of electron density on the cyanide ligands and thereby increase the π-backbonding with the metal. These strong solute–solvent bonds formed between the cyanides and the nearest water molecules are expected to give rise to the observed strong solvatochromism (Section S3.13). Here, we note that even though the MLCT transitions involve both the Fe(CN)_4_ moiety and the bipyridine ligand, the negative charge on the latter is rather delocalized, and thus this ligand does not significantly contribute to the solvatochromism.

In the electronic excited state at later times (red curves in Figure 4b–d), the cyanide–water interactions remain strong, although slightly weakened compared to the ground state, as observed from a small broadening and shift to slightly longer distances in the corresponding RDFs. Similar weakening trends were reported^83^ for the hexacyano complex [Fe^II^(CN)_6_]^4–^ compared to [Fe^III^(CN)_6_]^3–^. In this case, the observed weakening was ascribed to the overall smaller charge of the complex, which consequently reduces the electrostatic interaction between the cyanides and the nearest solvent. For the case of [Fe(CN)_4_(bpy)]^2–^, the overall charge of the complex remains the same during excitation, but the initially populated MLCT states formally correspond to a reduced bpy fragment and an oxidized Fe(CN)_4_ fragment, which likely changes the cyanide–water interactions in a similar way to oxidation of [Fe^II^(CN)_6_]^4–^. The difference RDFs (Figure 4e–g) depict an almost instantaneous change of solvation after excitation, which suggests that the solvent starts to reorganize as a response to changes in charge distribution, rather than as a response to the structural changes within the solute. This is evident from the observation that the main bond elongations of the solute (Figure 3) take approximately the same time (about 50–100 fs) as this solvent reorganization. We assume that the solvent relaxation dynamics also directly affects the solute CT dynamics. Solvent relaxation leads to weaker hydrogen bonds, which lowers the energy of MLCT states (Section S3.13), thus presumably slowing down charge transfer (relative to the hypothetical situation that solvent is present but frozen to the ground-state distribution). On the contrary, ISC occurs between states of similar CT character, and hence solvent relaxation does not notably affect the electron spin dynamics. Future studies could be envisioned to examine trends in other H-bond donating solvents such as methanol, ethanol, and propanol, as well as other polar aprotic solvents such as DMSO or ACN.

The calculated XSS difference signals reflect some of the solute and solvent structural changes discussed above. The solute dynamics show a low-*Q* signal growing with a time scale of 160 fs, which exhibits coherent oscillations with a period of ∼92 fs that arise from the coherent oscillations observed in the bipyridine ligand. Importantly, we also show that there is a significant difference (both in shape and amplitude) in the XSS signal that arises from an optimized solute structure compared to the averaged nonadiabatic trajectories. There are multiple effects contributing to this observation. First, optimized structures represent the molecule in a cold state, whereas the excited molecule contains a large amount of excess energy, such that different parts of the PESs are visited that could not be visited by a cold molecule. Second, the nonadiabatic trajectories include multiple different MLCT and MC states (excitations from different t_2g_ orbitals into different π* and e_g_ orbitals) and the different forces exerted on the nuclei if these states are populated. Conversely, the optimized structures are typically only obtained for the lowest MLCT or MC state. As shown in Figure 2, in our simulations even after 700 fs almost half of the trajectories are not yet relaxed to the lowest adiabatic triplet surface, showing that multiple MLCT or MC states are relevant. We suggest that this dynamical effect should be actually observable by XSS experiments with sufficient statistics and time resolution and an appropriate *Q*-range coverage. This would enable a detailed structural refinement of time-resolved scattering data, providing access to the structural distribution in the hot MC state and potentially in the MLCT states of the solute. Conversely, this also implies that future high-resolution XSS experiments should not rely on optimized structures but rather on dynamics simulations.

The simulated solute–solvent cross term evidences both fast (almost instantaneously) and slower dynamics (above 200 fs), with a particularly interesting positive feature at *Q* below 1 Å^–1^ that appears on a time scale of about 50 fs. This feature arises from the reorganization dynamics of the solvent around the cyanide ligands (recall the RDFs for N and C_CN_ in Figure 4f–g and Section S3.5). Our simulations thus predict that XSS experiments can observe such ultrafast solvent reorganization dynamics (as has been reported previously^67^), given that such experiments continuously improve in terms of resolution in both space (through higher *Q*-space coverage) and time domains. We hope that the present nonadiabatic dynamics simulations stimulate such time-resolved XSS experiments.

## Conclusion

We have simulated the ultrafast excited-state dynamics of the transition metal complex [Fe(CN)_4_(bpy)]^2–^ in water. Using hybrid QM/MM surface hopping dynamics, we have disentangled the different interactions that connect the electronic and nuclear degrees of freedom of the solute and the solvent, once they are activated upon irradiation.

**Figure 8:**
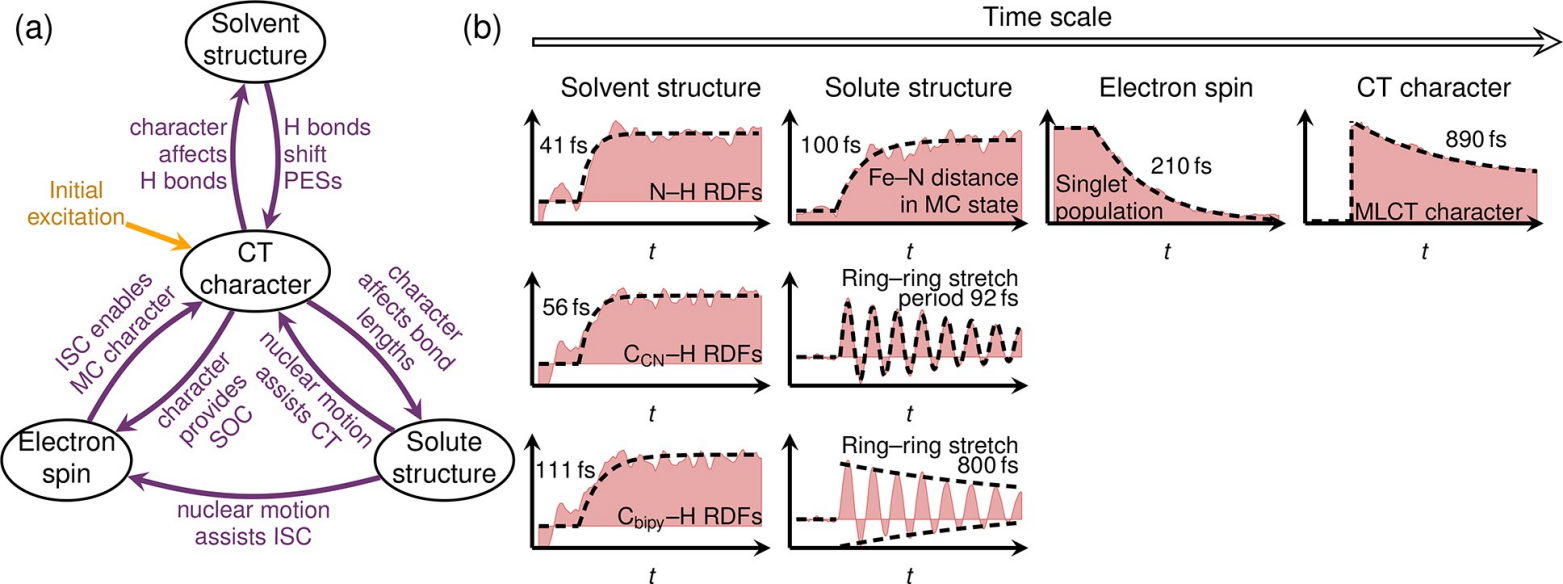
(a) Considered degrees of freedom in [Fe(CN)_4_(bpy)]^2–^, with main processes and interactions. (b) Temporal evolution of exemplary degrees of freedom in a time line.

Based on a comprehensive analysis of the trajectories, here we propose a holistic interpretation of the dynamics of the system. To this aim, in Figure 8 we gather the involved processes and their interactions, as well as the temporal dynamics through which they manifest. The electronic degrees of freedom of the solute are collectively addressed as the “electron spin” and the “CT character”, which characterize the multiplicity as well as the particular electronic transitions, respectively. The complementary nuclear degrees of freedom include the “solute structure”, best illustrated with bond length changes, and the “solvent structure”, best visualized in the form of SVD traces of the RDFs. As sketched in Figure 8a, the initial electronic excitation, which initiates the change from a closed-shell electronic state to an MLCT state, starts the dynamics and directly affects all other degrees of freedom. The change of occupation of the metal d orbitals significantly increases the SOC, or in other words, the CT character controls ISC. At the same time, the CT character also affects both the solute structure and solvent structure. The first is reflected, for example, by the oscillations in the bpy ligand in the MLCT states as well as in the increased Fe–N and Fe–C bonds in the MC state. The second can be seen in the immediate reduction of hydrogen bonding after excitation. In turn, these degrees of freedom also affect the evolution of the CT character. ISC is a prerequisite for populating states of MC character, because ^1^MC states are not accessible. Additionally, both solvent and solute nuclear motion are required because otherwise the energies of MLCT and MC states would not be tuned for a transition to take place. In turn, the solvent structure, e.g., in the form of hydrogen bonds, modulates strongly the relative energy of MLCT and MC states, giving rise to the strong solvent dependence of the photoinduced processes in [Fe(CN)_4_(bpy)]^2–^. Likewise, the solute structure seems to be strongly connected to the electron spin, as without nuclear relaxation there is a dramatic decrease of ISC.

Although these processes are intertwined and many overlap in time, it is particularly interesting that they seem to follow an unconventional time line. After excitation, the fastest among the mentioned processes appears to be the solvent response; see sketch in Figure 8b. Specifically, the changes in hydrogen-bonding structure involving the cyanides take place on a 50 fs time scale. This is in contrast with the expected ultrafast structural response of the solute, e.g., the elongation of the Fe–N bonds after entering the MC state, which take place more slowly, on a 100 fs time scale. The response of the bpy ligand to charge transfer occurs on a similar time scale, with a 92 fs oscillation period (and damping of that oscillation within several hundred fs). Remarkably, the response of the electronic degrees of freedom is even slower than the solute fastest relaxation; that is, the spin flip takes place within 200 fs and the MLCT → MC interconversion requires several hundred fs. Hence, even if our simulations show that electronic excitation induces ultrafast responses in all the nuclear and electronic degrees of freedom, not all of them are coupled with each other to the same extent. In this particular case, specific solvent reorganization seems to be immediate, followed by nuclear relaxation of the solute that drives ISC and CT dynamics.

In order to allow future experimental work to support or challenge our simulations, we have calculated time-resolved X-ray solution scattering difference signals based on contributions from the solute, solvent, and solute–solvent cross term. Encouragingly, the solute–solvent and solvent scattering contributions indicate that this unusually fast solvent reorganization could be observed experimentally; thus, we hope that this work motivates experiments in this direction.

## Supporting Information Available

Supplementary method and analysis details; supplementary results (orbitals, vertical excitations, charge transfer analysis, optimized/average structures, solvent structure, one-dimensional X-ray scattering signals, vibrational analysis, solvent shifts) (PDF)
