# Supplementary material for: Resolving Femtosecond Solvent Reorganization Dynamics in an Iron Complex by Nonadiabatic Dynamics Simulations
Source: J Am Chem Soc. 2022 Jul 1;144(28):12861–73. doi: 10.1021/jacs.2c04505 (PMC9305979; doi:10.1021/jacs.2c04505)
Supplement: Supplementary file 1 — ja2c04505_si_001.pdf [file ja2c04505_si_001.pdf]

Supporting Information: Resolving femtosecond solvent reorganization dynamics in an iron complex by nonadiabatic dynamics simulations

Diana Bregenholt Zederkof,[†,‡] Klaus B. Møller,[¶] Martin M. Nielsen,[†] Kristoffer Haldrup,[†] Leticia González,[§] and Sebastian Mai[*,§]

†*Department of Physics, Technical University of Denmark, Fysikvej, bygning 307, 2800 Kongens Lyngby, Denmark.*

‡*Scientific Instrument Femtosecond X-ray Experiments, European XFEL GmbH, Holzkoppel 4, 22869 Schenefeld, Germany.*

¶*Department of Chemistry, Technical University of Denmark, Kemitorvet, bygning 207, 2800 Kongens Lyngby, Denmark.*

§*Institute of Theoretical Chemistry, Faculty of Chemistry, University of Vienna, Währinger Straße 17, 1090 Vienna, Austria.*

E-mail: sebastian.mai@univie.ac.at

Contents

S1 Methods **S-3**
S1.1 Supplementary quantum chemistry calculations S-3
Effect of GGA exchange S-4
S1.2 Initial condition generation S-5
S1.3 Electronic structure level of theory S-9
S1.4 SHARC excited state dynamics S-9
S1.5 SHARC frozen-nuclei dynamics S-10

S2 Data Analysis **S-11**
S2.1 Analyzed set of trajectories S-11
S2.2 Electronic representations and populations S-11
S2.3 Charge transfer character analysis S-12
S2.4 Vibrational analysis S-13
S2.5 Radial distribution functions S-13
S2.6 Calculation of X-ray solution scattering signals S-13

S3 Supplementary results **S-16**
S3.1 Vertical excitations using implicit solvation S-16
S3.2 Charge transfer characters in the simulated absorption spectrum S-20
S3.3 Charge transfer character from individual fragments S-21
S3.4 Comparison of averaged goemetries from explicit solvation and optimized geometries from implicit solvation S-22
S3.5 Additional RDFs S-23
S3.6 SVD of RDFs S-24
S3.7 Charge transfer weighted RDFs S-26
S3.8 RDFs of equatorial versus axial cyanides S-28
S3.9 Hydrogen bonds S-30
S3.10 Angle-resolved RDFs S-32
S3.11 X-ray solution scattering signals S-35
S3.12 Vibrational analysis using implicit solvation S-37
S3.13 Solvent effects on electronic energies S-39

S4 Cartesian coordinates of optimized structures **S-41**

Supplementary References **S-43**

S1 Methods

This section provides additional details on the computational details carried out in this study. In the following sections, we first describe the set of supplementary quantum chemistry calculations. Subsequently, we describe all steps of the nonadiabatic dynamics simulations: the sampling of initial conditions in aqueous solution; the employed electronic structure methods; the nonadiabatic dynamics simulations including internal conversion, intersystem crossing, and solvent dynamics; and the comparative frozen-nuclei dynamics.

S1.1 Supplementary quantum chemistry calculations

We performed a number of supplementary quantum chemistry calculations to initiate and support our discussion in the main text.

In the first set of calculations, we optimized the equilibrium geometries of the singlet ground state and the lowest triplet MLCT and MC states. Initial geometries for the latter two were taken from suitable snapshots from the excited-state SHARC trajectories. Whereas all other computations in this work were conducted with ORCA (see below), these optimizations were carried out with Gaussian 16[S1] due to the availability of analytical TDDFT frequencies in the presence of implicit solvent. For full consistency with the SHARC simulations (see below in Section S1.3), we adjusted the Hartree-Fock and GGA exchange to match the B3LYP* settings we used for the ORCA calculations described below (B3LYP with VWN5, 15% Hartree-Fock exchange, and 85% GGA exchange). The lowest triplet surface was described with the Tamm-Dancoff approximation (TDA), the solvent with the IEFPCM method,[S2,S3] and dispersion through the D3 correction.[S4] Optimizations and frequency calculations for all three minima were carried out in three solvents (water, acetonitrile, DMSO).

In the second set of simulations, we performed vertical excitation calculations at the optimized ground state geometries. The electronic structure settings were the same as described in detail in Section S1.3. We computed 30 singlet and 30 triplet states, using TDA and implicit solvation through C-PCM. These calculations were carried out for acetonitrile, DMSO, and water with ORCA.

The third set of calculations was carried out to investigate the effect of using the non-standard value of 85% GGA exchange in the B3LYP* functional instead of 72%. Here, we also computed 30 singlet and 30 triplet states, using TDA and implicit solvation through C-PCM (for water). We carried out two such calculations, once with the default GGA exchange and once with the modified value of 85%. These calculations were carried out with ORCA. Subsequently, we computed wave function overlaps between the two sets of TDA response vectors, using the WFoverlap code.[S5]

Effect of GGA exchange

The results of the last set of calculations is shown in Figure S1. Both calculations use identical settings, except for the contribution of GGA exchange. As can be seen, the change in GGA exchange affects the results only to a minor extend, especially for the low-lying states. The RMSD of all 60 excitation energies is 0.021 eV, whereas the RMSD for the states included in the dynamics simulations (S_1 to S_5 and T_1 to T_7) is only 0.013 eV. The state characters are also very well preserved, with every state from one calculation overlapping at least to 96% with the corresponding state from the other calculation. For the states included in the dynamics, the overlaps are at least 99.7%. This shows that the different GGA exchange fraction does not notably affect our results.

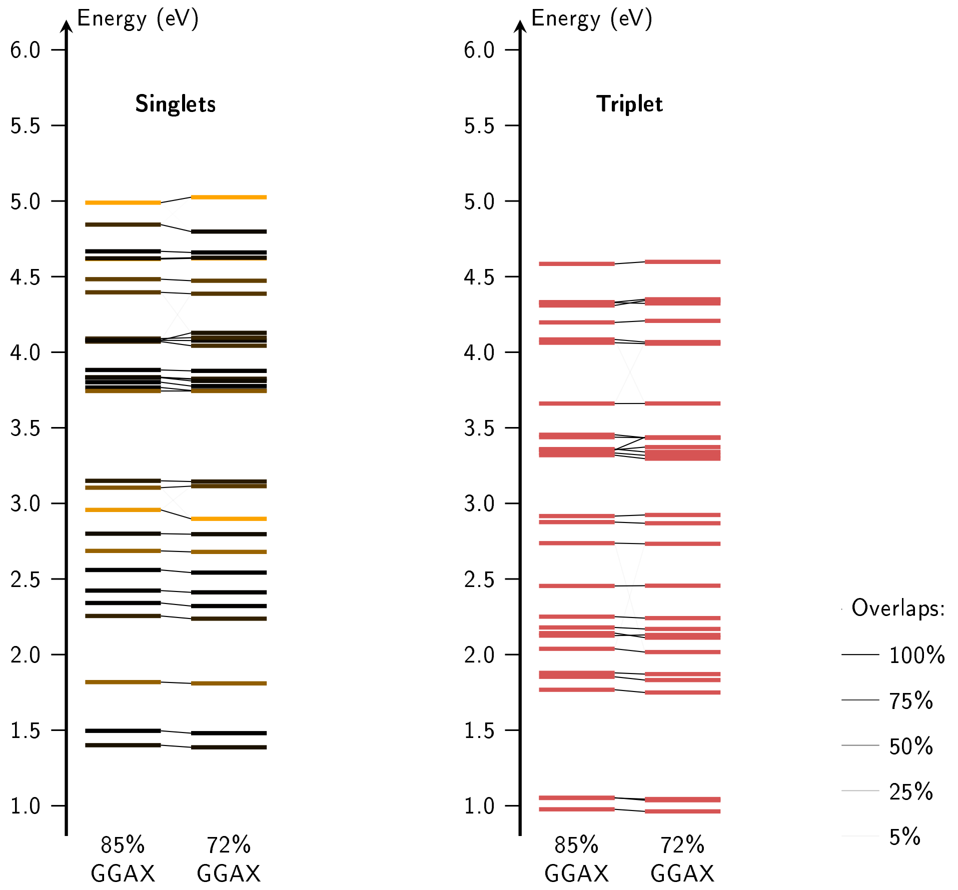

Figure S1: Vertical excitation energies, oscillator strengths (black is dark, orange is bright, red is triplet), and wave function overlaps correlating the states of two calculations with B3LYP* using the value of 85% for the GGA exchange (as used in the present work) and the value of 72% (as originally proposed for B3LYP*.[S6]

S1.2 Initial condition generation

The first step in the excited-state dynamics simulations is the preparation of the initial conditions. Within the SHARC package, this includes the initial coordinates and velocities of all atoms (solute and solvent), as well as the initial active electronic state and initial electronic wave function coefficients. In the present work, these were obtained in two stages, first preparing the nuclear positions/velocities and subsequently fixing the electronic quantities.

In the first stage, we prepared the initial nuclear coordinates and velocities. The general preparation procedure is described and discussed in Ref. S7, and presented schematically in Figure S2. To produce appropriate initial conditions for the solute embedded in a box of water, we first carried out classical MD simulations using AMBER17.[S8] For the iron complex, an preliminary force field was created using the python-based metal center parameter builder, `mcpb.py`, described in Ref. S9 (see below for refinement details). The equilibrium geometry and corresponding frequencies were computed at the B3LYP/LANL2DZ[S10–S12] level of theory in gas phase. From the geometry and frequencies, `mcpb.py` derived initial bond, angle, and dihedral terms based on the Seminario method.[S13] The point charges were then computed using the RESP method[S14] from the electron density obtained at the optimized geometry with the same method, but including solvent effects through the IEFPCM (water) method.[S2,S3] These density functional calculations were carried out with Gaussian 09.[S15]

The system used throughout all dynamics simulations was built from the iron complex that was solvated in a truncated octahedron water box with a smallest radius of about 29.7 Å (25 Å distance between the solute atoms and the nearest faces). The solvent was represented by 5412

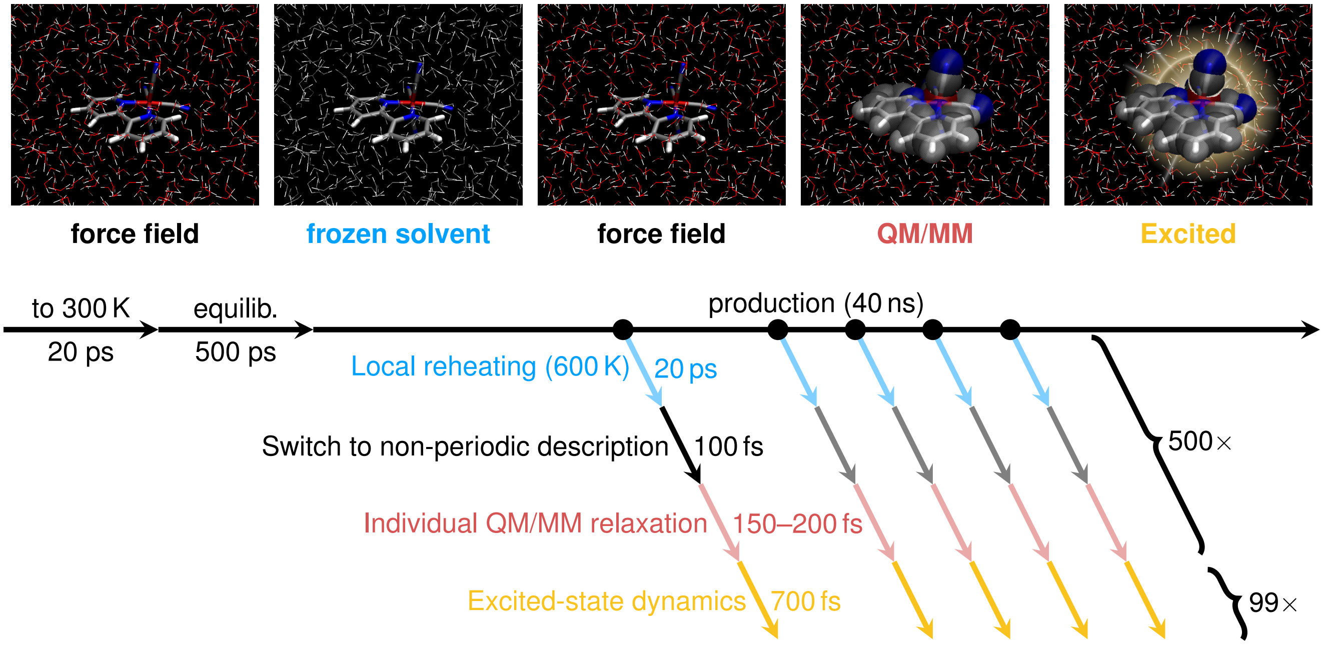

Figure S2: Schematic of the initial condition generation. The system (complex, two Na^+ counter ions, 5412 water molecules) was first heated to 300 K over 20 ps and equilibrated to 300 K and 1 bar over 500 ps. Then, 500 snapshots were extracted from a 40 ns simulation. All these steps were done at MM-MD level. For each snapshot, the solute was then reheated to 600 K with frozen solvent, before the system was switched to non-periodic conditions. Each snap was then propagated for 150–200 fs in the ground state (using QM/MM). Of the 500 snapshots, 116 were selected for excitation and propagated for 700 fs in the excited state using SHARC. 99 trajectories were used for the analysis. For further details, see text.

flexible-type SPC/Fw[S16] water molecules, plus two sodium ions for neutralization. The system was first minimized, followed by a thermalization run to 300 K (NVT ensemble) for 20 ps, and an equilibration run to 1 bar for 500 ps (NPT ensemble).

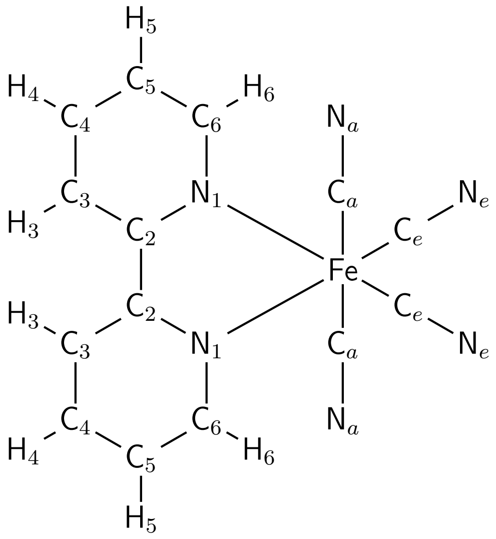

Figure S3: Connectivity and atom labeling for the force field parameters in Tables S1 and S2.

In order to refine the bond length parameters of our force field, we ran a 3 ps QM/MM trajectory (time step 0.5 fs, NPT ensemble) to serve as reference. This calculation was done in AMBER using the AMBER-ORCA interface[S17] using the B3LYP* functional,[S6,S18] that has only 15% of Hartree-Fock exchange (compared to 20% in B3LYP) and was shown to work very well for iron complexes.[S19,S20] The calculations employed the ZORA-def2-SVP basis set,[S21] the ZORA scalar relativistic correction,[S22] and the D3 dispersion correction.[S4] The distributions of bond lengths from the QM/MM trajectory was then compared to the results of an MM MD simulation using the initial force field, and the equilibrium bond lengths were adjusted to match the averages. The angles were not adjusted since they were already in good agreement with the distributions of angles from the QM/MM trajectory. The average bond lengths were calculated from fits of histograms of the different types of bonds. The bond distributions were assumed to follow a normal distribution and fit with a Gaussian function, $G(r)$:

$$G(r) = He^{-(r-\mu)^2/(2\sigma^2)} \tag{1}$$

where H is the maximum height of the curve, μ is the expectation value and thus the calculated average bond length of a given bond, and σ is the standard deviation. The adjusted equilibrium bond lengths R_{equil} and estimated mean values μ are given in Table S1. The associated force constants K_{bond} were updated accordingly from a ratio of the MM constants before adjustments, $K_{\text{bond}}^{\text{MM}}$ and the QM/MM constants, $K_{\text{bond}}^{\text{QMMM}}$ squared multiplied with the standard deviation σ_{MM} of the MM bond distributions, squared.

Using the updated force field, an MM MD production run of 40 ns was carried out and 500 snapshots, equally distributed with time spacing of 80 ps, were collected. This set of instantaneous geometries and velocities of the whole system was then further prepared. Since pure classical MD simulations at 300 K leads to too low internal energy of the metal complex compared to the zero-point energy,[S23–S25] we applied a small local temperature adjustment procedure to account for this effect, as discussed in Ref. S7. Hence, each of the 500 snapshots were briefly re-heated to 600 K for 20 ps with frozen solvent atoms.

After the local reheating step, the following simulations were run without periodic boundary

Table S1: Force field parameters used in the MM MD simulations (K_{bond}, R_{equil}, K_{angle}, θ_{equil}, σ, ε) and (for bonds and angles) comparison between averages μ from QM/MM MD and MM MD before and after updating the equilibrium distances and forces.

— Bond parameters —					
Atoms	K_{bond} (kcal/mol/Å^2)	R_{equil} (Å)	$\mu_{\text{QM/MM}}$ (Å)	$\mu_{\text{MM}}^{\text{after}}$ (Å)	$\mu_{\text{MM}}^{\text{before}}$ (Å)
Fe–N_1	34.5	1.954	2.029	2.035	2.038
Fe–C_a	123.0	1.952	1.942	1.946	1.965
Fe–C_e	99.0	1.915	1.914	1.915	1.947
C_a–N_a	954.6	1.174	1.179	1.180	1.159
C_e–N_e	954.6	1.174	1.180	1.178	1.159
N_1–C_2	391.7	1.348	1.362	1.364	1.353
C_2–C_3	375.3	1.392	1.406	1.408	1.420
C_3–C_4	469.9	1.392	1.397	1.397	1.404
C_4–C_5	353.9	1.399	1.403	1.403	1.402
C_5–C_6	433.2	1.391	1.397	1.398	1.405
C_6–N_1	401.1	1.332	1.348	1.347	1.356
C_2–C_2'	288.3	1.465	1.474	1.479	1.495

— Angle parameters —				
Atoms	K_{angle} (kcal/mol/rad^2)	θ_{equil} (degree)	$\mu_{\text{QM/MM}}$ (degree)	μ_{MM} (degree)
N_1–Fe–N_1'	91.1	81.5	80.1	80.0
C_a–Fe–C_e	124.7	89.6	90.7	89.9
C_a–Fe–N_1	123.1	90.4	89.3	90.1
N_1–Fe–C_e	113.9	92.9	94.3	94.3
C_e–Fe–C_e	151.9	92.7	90.7	91.2
N_1'–Fe–C_e	113.9	174.4	175.9	174.3
C_a–Fe–C_a	104.5	179.0	183.1	183.3
Fe–C_a–N_a	36.2	174.9	182.4	176.5
Fe–C_e–N_e	36.9	174.5	178.0	174.2
Fe–N_1–C_2	120.6	115.7	114.8	113.7
Fe–N_1–C_6	133.4	125.9	125.9	126.5

— Lennard–Jones parameters —		
Atom	σ (Å)	ε (kcal/mol)
Fe	1.409	0.01721
N_1	1.824	0.17000
C_a	1.908	0.21000
C_e	1.908	0.21000

conditions, and thus all atoms were imaged into the primary cell, with the solute centered. The system was propagated in the NVE ensemble for a short time of 100 fs, in order to allow the solvent to respond to the reheated solute, but still avoiding too much energy being transferred from the solute to the solvent. The coordinates and velocities from the last frame of the short re-equilibration step were then collected and converted into the SHARC initial condition format.[S7]

As we are especially interested in the change in dynamical behaviour after the excitation, for each initial condition we ran a short ground state QM/MM MD simulation using SHARC at the same level of theory also used for the excited-state dynamics, i.e., using DFT instead of TD-DFT

Table S2: Partial charges obtained from RESP fitting of the B3LYP/LANL2DZ/CPCM electron density.

Fe	-0.0177
C_a	0.1753
N_a	-0.7845
C_e	0.1792
N_e	-0.7959
N_1	0.0572
C_2	0.1026
C_3	-0.2064
C_4	-0.1098
C_5	-0.1343
C_6	-0.1730
H_3	0.1754
H_4	0.1682
H_5	0.1624
H_6	0.1923

(see Section S1.3 for details on the electronic structure method). These short QM/MM trajectories were run for a randomized time between 150–200 fs in order to avoid possible effects arising from the switch in level of theory in the later excited-state dynamics simulations. The endpoints of the trajectories from the individual QM/MM relaxation step were collected and constituted the final coordinates and velocities for the excitation process, concluding stage one of the initial condition generation process.

In the second preparation stage, for each of the 500 initial geometries we selected the initial electronic excited state. For this, we first carried out a vertical excitation calculation at each geometry, computing 20 singlet and 20 triplet states (see Section S1.3 for the level of theory). From the resulting excitation energies and oscillator strengths, we computed the absorption spectrum shown in the main manuscript, Figure 1c, from a convolution using Gaussians of 0.1 eV full width at half maximum. The triplet states did not contribute to the absorption spectrum and were computed to estimate the density of triplet states and the number of triplet states to include in the dynamics simulations.

The selection of the initial electronic state is modeled by an instantaneous excitation process.[S26] In order to compare the results to optical studies of the same system,[S27,S28] we wished to excite at 500 nm, corresponding to 2.5 eV. As the simulated steady state absorption spectrum is slightly shifted (approx. 45 nm) relative to the experimental one, we decided to shift the desired excitation energy to the maximum wavelength in the simulated spectrum, i.e., to 545 nm or 2.3 eV. In order to excite a statistically sufficient fraction of the 500 initial conditions, we broadened the excitation window and excited at 2.35±0.10 eV (506–551 nm), which resulted in 116 of the 500 geometries being excited into the S_3 state, which is the only bright state within the first absorption band according to Figure 1c in the main manuscript. These 116 geometries (with corresponding velocities) constituted the starting point of the excited state dynamics simulations. The initial wave function coefficients were set such that the spin-free S_3 state has a coefficient of 1. The spin-mixed state that has the largest contribution from the S_3 was chosen as the initial active state.

S1.3 Electronic structure level of theory

The electronic structure calculations involved in the ground state QM/MM dynamics, vertical excitation energies, and the excited state dynamics simulations all used the same level of theory. The calculations were carried out with ORCA, version 4.1.

We chose to employ the B3LYP* functional[S6,S18] that has only 15% of Hartree-Fock exchange (compared to 20% in B3LYP) and was shown to work very well for iron complexes.[S19,S20] This was accomplished in ORCA by adding the keywords `ScalHFX 0.15` and `ScalDFX 0.85`, as was given in the ORCA 4.1 documentation. Hence, the functional used in our computations is slightly different from the original definition of B3LYP*. However, above (Section S1.1) we present data comparing the original B3LYP* and our settings, showing very small deviations and indicating that this difference to the original definition is inconsequential for our conclusions.

As a compromise between accuracy and efficiency, the calculation employed a mixed-ζ basis set combination, using the ZORA-def2-SVP basis set for C, N, and H, and the ZORA-def2-TVP basis set for Fe.[S21] Scalar relativistic effects were described with the ZORA Hamiltonian,[S22] and the empirical D3 dispersion correction[S4] was also used. The calculations were sped up with the RIJCOSX approximation implemented in ORCA[S29] using the SARC/J auxiliary basis set.[S30] Excited states were computed with the TDA.[S31]

In the QM/MM simulations, the metal complex was located in the QM region, whereas the water molecules and two sodium ions constituted the MM region. The interaction between these regions was described with electrostatic embedding, as implemented in ORCA. In the dynamics simulations carried out with SHARC, the MM energy and gradients were computed with Tinker 6.3.3,[S32] interfaced to ORCA through the SHARC–ORCA interface code. The force field parameters for water and sodium ions were taken from the AMBER files described above and translated into Tinker input format.

S1.4 SHARC excited state dynamics

Based on the choice of the initially active state and the density of singlet and triplet states, we chose to include the 6 lowest singlets (including the ground state, i.e. S_0-S_5) and 7 triplets (T_1-T_7)—giving a total of 27 states ($6 + 3\times7$)—in the dynamics simulations. The length of the simulations was 700 fs, using time steps of 0.5 fs for the nuclear propagation. The electronic wave functions were propagated with the local diabatization method[S33] and the three-step propagator method of SHARC[S34] using a time step of 0.02 fs. The required wave function overlap matrices were computed with the WFoverlap program,[S5] treating the TDA response vectors as CI vectors and truncating every vector to 99.95% of its norm to speed up the overlap computations. The wave function propagation also included the spin–orbit matrix elements provided by ORCA.

The dynamics simulations carried out with SHARC (150–200 fs in the ground state, as discussed above, and 700 fs in the excited state) were run with no periodic boundary conditions and no thermostat, and hence in the NVE ensemble. This is reasonable as the simulations are short enough that the Fe complex is not notably affected by the solvent–vacuum interface (see also below). In order to keep the total energy constant during a surface hop, the velocity vector of the atoms of the Fe complex were rescaled appropriately. An energy-based decoherence correction scheme[S35] was applied to the diagonal states in order to account for the well-known over-coherence problem in surface hopping. The velocities of the MM region were not considered when rescaling the velocity vector or for the decoherence correction. In order to compute the gradient of the currently active diagonal state, a linear combination of the gradients of all MCH states that were within 0.15 eV to the active state was formed.[S36]

S1.5 SHARC frozen-nuclei dynamics

For the SHARC dynamics simulations with frozen nuclei, we performed a single-point calculation for each of the 116 initial geometries, using 6 singlets and 7 triplets and including spin-orbit couplings. The resulting data was then fed into the SHARC dynamics driver at every time step, using zero initial velocities, zero gradients, and unity overlap matrices. The frozen-nuclei simulations were propagated until 500 fs with a 0.5 fs time step (for write out) and a 0.02 fs time step for the electronic wave function. Note that in the main text we only show the first 135 fs, as the later part of the frozen dynamics does not provide additional useful information. As the overlap matrices were set to unity, only the spin-orbit couplings induce state-to-state population transfer. For comparison purposes, the electronic wave function was subjected to the same energy-based decoherence correction scheme[S35] as in the full simulations.

S2 Data Analysis

In this section we describe all methods used to analyze the results of the nonadiabatic dynamics simulations.

S2.1 Analyzed set of trajectories

We propagated all 116 initial conditions described above. Unfortunately, network errors corrupted the restart files of 17 of the trajectories, so that they could not be propagated until the 700 fs total simulation time. Thus, the results presented in the main manuscript and here are based on the 99 trajectories that completed the 700 fs simulation time. As the aborted trajectories are not due to convergence errors or other problems in the electronic structure calculations, we do not expect that the reduction of the ensemble introduces a bias in the results. The only expected effect is a slight increase of the uncertainties of the results.

S2.2 Electronic representations and populations

Within the SHARC method, two main representations of the electronic state basis are employed, called the *molecular Coulomb Hamiltonian* (MCH) and the *diagonal* representations.[S34] In the MCH representation, the electronic basis states are the eigenstates of the molecular Coulomb Hamiltonian $\hat{H}^{\mathrm{MCH}}$ (the clamped nucleus Hamiltonian including only electronic kinetic energy and Coulomb terms) that are directly obtained within the electronic structure code, here ORCA. In the diagonal representation, the basis states are the eigenstates of the total electronic Hamiltonian $\hat{H}^{\mathrm{tot}}$ that also includes additional terms, here spin–orbit couplings. Directly computing these eigenstates with electronic structure methods is non-trivial and very expensive, and often not available in electronic structure packages. Hence, in SHARC, we only compute the eigenstates of $\hat{H}^{\mathrm{tot}}$ in the subspace of the fewest MCH eigenstates. In order to do so, we first compute a set of MCH states using ORCA (i.e., 6 singlet and 3×7 triplet states) and the matrix form of $\hat{H}^{\mathrm{tot}}$ in this basis, with elements $H_{ij}^{\mathrm{MCH}} = \langle \Phi_i^{\mathrm{MCH}} | \hat{H}^{\mathrm{tot}} | \Phi_j^{\mathrm{MCH}} \rangle$. This matrix is denoted as $\mathbf{H}^{\mathrm{MCH}}$ (the superscript indicating the basis, not the operator). We then obtain the diagonal states by a matrix diagonalization

$$\mathbf{H}^{\mathrm{diag}} = \mathbf{U}^{\dagger} \mathbf{H}^{\mathrm{MCH}} \mathbf{U}, \tag{2}$$

where $\mathbf{U}$ is the matrix containing the eigenvectors of $\mathbf{H}^{\mathrm{MCH}}$ and which serves as basis transformation matrix. The diagonal elements of $\mathbf{H}^{\mathrm{diag}}$ are the eigenenergies that are used as the potential energy surfaces for the nuclear dynamics and surface hopping. The gradients of these potential energy surfaces are computed as described previously.[S36] Knowing the matrix $\mathbf{U}$ also allows transforming the electronic results (populations, energies, oscillator strengths, charge transfer data) between both representations during analysis.

The populations in the MCH representation is calculated from an incoherent average over the set of trajectories, where for every trajectory the "quantum" amplitudes in diagonal representation are transformed in the MCH representation:

$$P_i^{\mathrm{MCH}}(t) = \frac{1}{N_{\mathrm{traj}}} \sum_{\mathrm{traj}} \left| \sum_{\alpha} U_{i\alpha} c_{\alpha}^{\mathrm{diag}}(t) \right|^2. \tag{3}$$

Here, N_{traj} is the number of trajectories, i is an MCH state, α is a diagonal state, and $c_{\alpha}^{\mathrm{diag}}$ is a time-dependent electronic wave function coefficient in the diagonal representation.

Note that by omitting the basis transformation in this equation, we could also compute the

populations in the diagonal representation. However, although they are necessary for the propagation algorithm (as they govern the hopping between the diagonal potential energy surfaces), they are very difficult to analyze. Hence, in the main manuscript, we discuss the electronic evolution only in terms of the MCH representation, which allows a direct interpretation of ISC as spin is a good quantum number in the MCH basis. We also note that neither diagonal nor MCH populations provide information on the electronic state characters (e.g., the T_1 MCH state could have MLCT character at one geometry and MC character at another one). Hence, the wave function character is separately analyzed, as described below.

S2.3 Charge transfer character analysis

The characters of the electronic states in the dynamics simulations were tracked at every time step in terms of charge transfer numbers computed with the TheoDORE program.[S37–S39] These charge transfer numbers are obtained from Löwdin population analysis of the TD-DFT transition density matrices between ground state and each of the excited states. TheoDORE uses molecular fragments, such that one obtains a matrix of charge transfer numbers for each pair of fragment. In the dynamics simulations, we saved charge transfer numbers for 21 fragments (each non-hydrogen atom considered as individual fragment) as 21×21 matrices with matrix elements Ω_{AB} (between fragments A and B). Such a matrix was computed for each of the 27 states (using a zero matrix for the ground state), for each of the 99 analyzed trajectories, and at each time step t. As visualization of this large data set is tricky, we simplify it in several steps. First, as we are only interested in the evolution of the actual electronic wave function, we average over all electronic states α using the MCH populations:

$$\Omega_{AB}^{\text{traj}}(t) = \sum_{i=1}^{N_{\text{MCH}}} \left| \sum_{\alpha=1}^{N_{\text{diag}}} U_{i\alpha}(t)\, c_{\alpha}^{\text{diag}}(t) \right|^2 \Omega_{AB}^{\text{state}\,i}(t), \tag{4}$$

where i runs over the MCH states and α runs over the diagonal states. $\Omega_{AB}^{\text{state}\,i}(t)$ is an element of the charge transfer matrix for MCH state i. This provides one charge transfer matrix, $\Omega_{AB}^{\text{traj}}$ for each time step and each trajectory. For additional analysis, we also computed the matrices $\Omega_{AB}^{\text{traj,S}}(t)$ and $\Omega_{AB}^{\text{traj,T}}(t)$ considering only the singlet or triplet contributions to the wave function, respectively.

Subsequently, we averaged the charge transfer matrices across all trajectories, resulting in one total charge transfer matrix, Ω_{AB}^{tot}, for each time step t:

$$\Omega_{AB}^{\text{tot}}(t) = \sum_{k=1}^{N_{\text{traj}}} \frac{\Omega_{AB}^{\text{traj},k}(t)}{N_{\text{traj}}} \tag{5}$$

where $\Omega_{AB}^{\text{traj}k}$ is the charge transfer matrix for trajectory k.

Third, after analysis of the 21×21 charge transfer matrices,[S39] we summed blocks of the matrices to obtain the MLCT, MC, LMCT, and LC characters of the entire ensemble in the following way:

$$\begin{aligned}
\Omega_{\text{MLCT}}(t) &= \sum_{A\in\text{Fe,CN}} \sum_{B\in\text{bpy}} \Omega_{AB}^{\text{tot}}(t) \\
\Omega_{\text{MC}}(t) &= \sum_{A\in\text{Fe,CN}} \sum_{B\in\text{Fe,CN}} \Omega_{AB}^{\text{tot}}(t) \\
\Omega_{\text{LMCT}}(t) &= \sum_{A\in\text{bpy}} \sum_{B\in\text{Fe,CN}} \Omega_{AB}^{\text{tot}}(t) \\
\Omega_{\text{LC}}(t) &= \sum_{A\in\text{bpy}} \sum_{B\in\text{bpy}} \Omega_{AB}^{\text{tot}}(t)
\end{aligned} \tag{6}$$

S2.4 Vibrational analysis

To analyze coherent vibrations of the solute after excitation, we aligned all frames of all excited trajectories using the Kabsch algorithm,[S40] computed the average trajectory, and subsequently computed vibrational modes by principal component analysis. The corresponding frequencies were obtained by Fourier transformation of the principal component mode coordinates.

S2.5 Radial distribution functions

Radial distribution functions (RDFs) were computed for all pairs of the following groups of atoms: Fe, C, N, H of solute, H of solvent, O of solvent. We first computed histograms accumulated over all 99 trajectories. For negative times (-150 to 0 fs), we accumulated the 99 ground state trajectories corresponding to the 99 excited state trajectories. The histograms were normalized by dividing with $4\pi R^2 \, \mathrm{d}R \, N_{\mathrm{traj}} \, N_{\mathrm{pairs}}/V$, where $\mathrm{d}R$ is 0.04 Å, N_{traj} is 99, $V = 4 \cdot (34.27\,\text{Å})^3$ is the volume of the truncated octahedron box, and N_{pairs} is $N_l N_m$ if l and m are different sets of atoms, and $N_l(N_l - 1)$ otherwise. The reference for the difference RDFs was computed by integrating the RDFs for all 500 ground state trajectories between -150 and 0 fs. As the last step, the RDFs and difference RDFs were smoothed by averaging in 10 fs intervals.

For the solvent-solvent contributions, we avoided effects due to the non-periodic boundary conditions by computing the RDFs only for atoms l that were within 17 Å to the Fe atom. Given the smallest radius of the truncated octahedron of about 29 Å, this allowed us to obtain the solvent-solvent RDF up to 12 Å. For these RDFs, the normalization constant was recomputed every time step as the 17 Å sphere did not contain a constant number of atoms.

Angular-radial distribution functions (ARDFs) were computed only for the hydrogen bonds around the cyanide ligands. We first obtained two-dimensional histograms for these pairs of variables: (i) $R(\mathrm{N_{CN}} - \mathrm{O_{water}})$ and $\alpha(\mathrm{N_{CN}} - \mathrm{H_{water}} - \mathrm{O_{water}})$, (ii) $R(\mathrm{N_{CN}} - \mathrm{O_{water}})$ and $\alpha(\mathrm{C_{CN}} - \mathrm{N_{CN}} - \mathrm{O_{water}})$, and (iii) $R(\mathrm{N_{CN}} - \mathrm{H_{water}})$ and $\alpha(\mathrm{C_{CN}} - \mathrm{N_{CN}} - \mathrm{H_{water}})$; axial and equatorial cyanide ligands were treated separately. Here, the first pair was used to estimate the number of hydrogen bonds using standard threshold values ($2.45\,\text{Å} \leq R(\mathrm{N_{CN}} - \mathrm{O_{water}}) \leq 3.55\,\text{Å}$ and $135^\circ \leq \alpha(\mathrm{N_{CN}} - \mathrm{H_{water}} - \mathrm{O_{water}}) \leq 180^\circ$). The other two pairs of variables were used to investigate the spatial distributions of H and O atoms around the cyanide groups. These histograms used larger bin widths of 0.5 Å and 30° to reduce the noise in the ARDFs. The normalization constant was used as before, but also included a $\sin(\alpha)$ factor.

S2.6 Calculation of X-ray solution scattering signals

Simulations offer a powerful complementary tool for interpretation of experimental observables. In particular, ultrafast time-resolved X-ray scattering experiments may show signals which are difficult to interpret. X-ray Solution Scattering (XSS) is a global probe observing changes in both the solute and solvent, and the changes are typically on the order of percentages or even permilles for these types of systems.[S41,S42] Here, computational methods may assist in reducing the complexity and extract potentially hidden information. In order to simulate the expected XSS signal, we only need information on the nuclear coordinates, which are easily obtained from simulations. From the interatomic distances, r_{ij}, the XSS signal S may be calculated by use of the Debye equation[S43]

$$S(Q) = \sum_{i,j=1}^{N} f_i(Q) f_j(Q) \frac{\sin Q\, r_{ij}}{Q\, r_{ij}} \tag{7}$$

where the sum runs over the number of atoms N, and f_i is the atomic form factor of atom i, and Q is the so-called scattering vector defining the difference between the incoming and outgoing wave vector of the X-ray radiation.

However, when including thousands of solvent molecules, it quickly becomes infeasible to use the Debye formula, since the sum runs over each atom. Alternatively, the scattering may be calculated from RDFs,[S44] as the ones computed as detailed above in section S2.5. Hence, RDFs act not only as convenient measures of the distribution of distances, but also provides a direct connection to experimental observables. Here, separate RDFs, $g_{l,m}(r)$ for each pair of atom types l and m are used. The total scattering S is then calculated according to:

$$S(Q) = \sum_{l \in t} N(l) f_l^2 + \sum_{l,m \in t} f_l(Q) f_m(Q) \ \frac{N(l)\left(N(m) - \delta_{lm}\right)}{V} 4\pi \int_0^R r^2 \left(g_{l,m}(r) - g_{l,m}^{\infty}\right) \frac{\sin(Qr)}{Qr} dr \quad (8)$$

where l and m run over all atom types in the system (t indicates the set of all atom types), and $N(l)$ is the number of atoms of type l. Furthermore, $f_l(Q)$ is the atomic form factor, V is the system volume, R is the maximum distance within the size of the simulated system, and $g_{l,m}^{\infty}$ is the distribution in the constant-density limit at long distances, which is either 1 or 0, if the RDFs are normalized.

For a system of a solvated metal complex it is favorable to analyze the scattering signal from different contributions to the total signal, since the complex and the solvent might change on different time scales. Therefore, we analyze the calculated scattering in terms of the solute-solute S_u, solvent-solvent S_v and solute-solvent cross S_c interactions.

$$\begin{aligned}
S_u(Q) &= \sum_{l \in u} N_u(l) f_l(Q)^2 + \sum_{l,m \in u} f_l(Q) f_m(Q) \frac{N_u(l)\left(N_u(m) - \delta_{l,m}\right)}{V} 4\pi \int_0^R r^2 \left[g_{l,m}(r)\right] \frac{\sin(Qr)}{Qr} dr \\
S_v(Q) &= \sum_{l \in v} N_v(l) f_l(Q)^2 + \sum_{l,m \in v} f_l(Q) f_m(Q) \frac{N_v(l)\left(N_v(m) - \delta_{l,m}\right)}{V} 4\pi \int_0^R r^2 \left[g_{l,m}(r) - 1\right] \frac{\sin(Qr)}{Qr} dr \quad (9) \\
S_c(Q) &= 2 \sum_{l \in u} \sum_{m \in v} f_l(Q) f_m(Q) \frac{N_u(l) N_v(m)}{V} 4\pi \int_0^R r^2 \left[g_{l,m}(r) - 1\right] \frac{\sin(Qr)}{Qr} dr
\end{aligned}$$

where $u = \{\text{Fe, N, C, H}_{\text{solute}}\}$ is the set of solute-type atoms, and $v = \{\text{H}_{\text{solvent}}, \text{O}_{\text{solvent}}\}$ is the set of solvent-type atoms in the system. Furthermore, for an atom of type l, then $N_u(l)$ and $N_v(l)$ states the number of atoms of that type l, in the solute and solvent, respectively.

As described in section S2.5, the RDFs were normalized slightly different for the solvent-solvent than solute-solute and solute-solvent cross interactions, which affects the appropriate calculation of the scattering. Thus, the number density term (N/V) in each expression was different for the solvent contribution than for the solute and cross terms. For the cross contribution the volume was the full size, $V = 4 \cdot (34.27\,\text{Å})^3$ and the number of atoms, were the following:

$$\begin{aligned}
N_u(\text{Fe}) &= 1 & N_u(\text{C}) &= 14 & N_v(\text{O}_{\text{solvent}}) &= 5412 \\
N_u(\text{N}) &= 6 & N_u(\text{H}_{\text{solute}}) &= 8 & N_v(\text{H}_{\text{solvent}}) &= 10824
\end{aligned}$$

Hence, using the RDFs and dividing the numerous atoms into different *types*, in contrast to summing over every single atom, greatly reduces the computational cost of calculating the scattering signals. For the solvent contribution, only the solvent atoms within 17 Å of the Fe-atom was considered and thus the volume within the 17 Å sphere around Fe was used, $V = \frac{4}{3}\pi \cdot (17\,\text{Å})^3$. As the number of solvent molecules within this sphere varied by less than 1%, the average number of atoms were

used, $N_v(\mathrm{O_{solvent}}) = 685.65$, $N_v(\mathrm{H_{solvent}}) = 1371.28$. We note that the calculated scattering of the solute and cross terms describe the scattering per solute molecule, whereas the solvent term only describes the solvent contribution of a theoretical sample containing ∼685 water molecules per complex molecule, corresponding to a concentration of ∼80 mM, and for comparison to experiments the solvent term should be rescaled to match experimental conditions.

Due to the finite size of the simulation "box", integration over the total box size might introduce unphysical truncation oscillations in the calculated scattering signal.[S45] To account for this problem, we employ a weight function, similar to other work,[S45–S47] which ensures that the lowest distances are given a higher weight, and long distances are set to zero.

$$w(r) = \begin{cases} 1, & \text{if } r < r_{\text{const}} \\ 1 - 3\left(\frac{r - r_{\text{const}}}{r_{\text{max}} - r_{\text{const}}}\right)^2, & \text{if } r_{\text{const}} \leq r \leq \frac{1}{3}(2\, r_{\text{const}} + r_{\text{max}}), \\ \frac{3}{2}\left(1 - \left(\frac{r - r_{\text{const}}}{r_{\text{max}} - r_{\text{const}}}\right)\right)^2, & \text{if } \frac{1}{3}(2 r_{\text{const}} + r_{\text{max}}) < r < r_{\text{max}}, \\ 0, & \text{if } r > r_{\text{max}}. \end{cases}$$

where $r_{\text{const}} = 5\,\text{Å}$ defines the lowest the distance for the cross term, without dampening the signal, $r_{\text{max}} = 25\,\text{Å}$ is the distance where the signal is believed to be converged, and hence the weight is set to zero for higher distances. For the solvent term, $r_{\text{const}} = 10\,\text{Å}$ and $r_{\text{max}} = 15\,\text{Å}$ based on a reliable region of the corresponding RDFs. Note, that the weight function is only relevant for RDFs involving solvent type atoms and thus only necessary for the cross term and solvent term.

In order to obtain the (time-dependent) difference scattering signals as are measured in XSS, we simply calculate it directly from the difference RDFs, $\Delta g(r,t) = g(r,t) - g(r)_{\text{average ground}}$, where the RDF from a given time step (-150-700 fs) is subtracted from the average of all RDFs before excitation. The difference scattering signals is then calculated in terms of contributions from the changes in the solute ΔS_u, solvent ΔS_v and cross term ΔS_c interactions, as shown in the main manuscript, Figure 5.

$$\begin{aligned} \Delta S_u(Q) &= \sum_{l,m \in u} f_l(Q) f_m(Q) \frac{N_u(l)\left(N_u(m) - \delta_{l,m}\right)}{V} 4\pi \int_0^R r^2 \left[\Delta g_{l,m}(r)\right] \frac{\sin(Qr)}{Qr} dr \\ \Delta S_v(Q) &= \sum_{l,m \in v} f_l(Q) f_m(Q) \frac{N_v(l)\left(N_v(m) - \delta_{l,m}\right)}{V} 4\pi \int_0^R r^2 w(r) \left[\Delta g_{l,m}(r)\right] \frac{\sin(Qr)}{Qr} dr \\ \Delta S_c(Q) &= 2 \sum_{l \in u} \sum_{m \in v} f_l(Q) f_m(Q) \frac{N_u(l) N_v(m)}{V} 4\pi \int_0^R r^2 w(r) \left[\Delta g_{l,m}(r)\right] \frac{\sin(Qr)}{Qr} dr \end{aligned} \quad (10)$$

S3 Supplementary results

S3.1 Vertical excitations using implicit solvation

This section gives a brief overview over the involved orbitals and state characters according to an implicit solvation calculation, and compare the results in different solvents. Figure S4 depicts the five Fe d orbitals and the four bpy π^* orbitals that are predominantly involved in all low-energy MLCT and MC states. Table S3 compiles the excitation energies, oscillator strengths, and leading orbital transitions of the low-lying electronic states (i.e., those that arise from the 18 transitions between the orbitals in Figure S4). The lowest-energy three MLCT states (both singlet and triplet) are the excitations from the three occupied d orbitals to the lowest π^* orbital, where the transition 80→82 corresponds to the bright S_3 state that is the initially excited one. The next three MLCT states are excitations to orbital 83, but these are typically 0.5–1.0 eV higher. The lowest three MC states are transitions from the three occupied d orbitals to orbital 86, which is the in-plane $d_{x^2-y^2}$. The next set of three MC states are excitations to orbital 87, which are 0.1–0.2 eV higher at the S_0 minimum and more than 2 eV higher at the optimized MC minimum. The fact that the in-plane $d_{x^2-y^2}$ orbital is slightly lower in energy affects the dynamics of axial and equatorial ligands, as discussed in the main text.

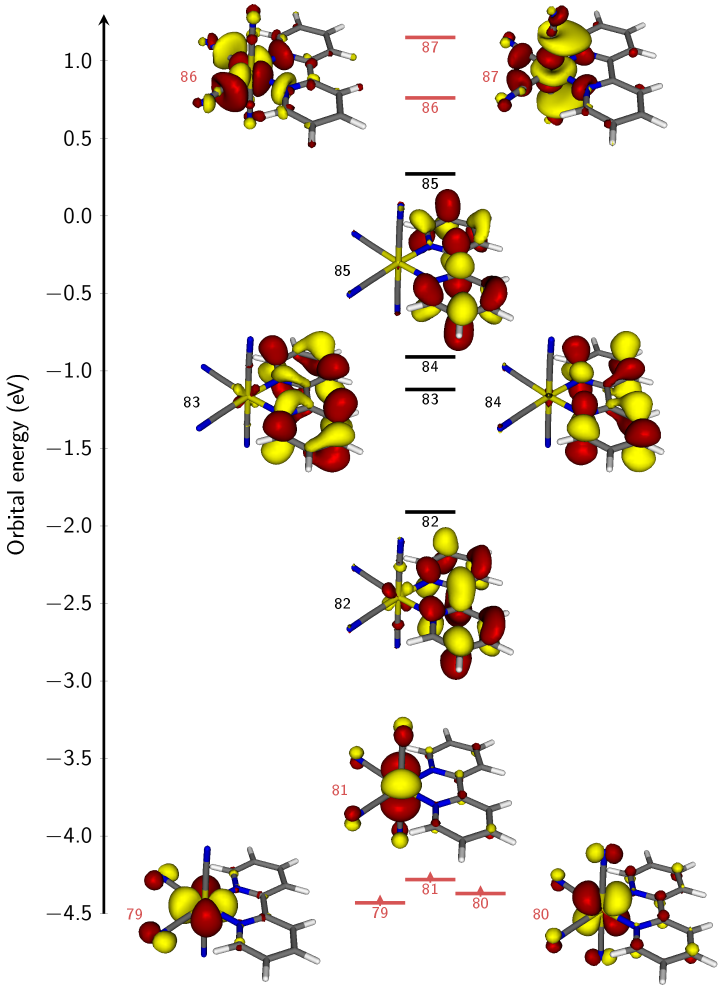

Figure S4: Depiction of molecular orbitals of $[Fe(CN)_4(bpy)]^{2-}$ at the S_0 minimum, computed using implicit solvation. Orbital energies for explicit solvation are discussed below in Figure S21.

Table S3: Excitation characters of electronic states in the B3LYP*/mixed basis vertical excitation calculation in implicit water solvation (Table S4 left column). t_{2g} orbitals are denoted as d, e_g orbitals as d^*. Only states arising from excitations within the orbitals in Figure S4 are shown.

State	E (eV)	f_{osc}	Weight	From	To	Character
S_1	1.40	0.00	98%	d_{A_2} (81)	π_1^* (82)	MLCT
S_2	1.50	0.00	97%	d_{A_1} (79)	π_1^* (82)	MLCT
S_3	1.82	0.03	88%	d_{B_1} (80)	π_1^* (82)	MLCT
S_4	2.26	0.00	96%	d_{A_2} (81)	π_2^* (83)	MLCT
S_5	2.34	0.00	95%	d_{A_1} (79)	π_2^* (83)	MLCT
S_6	2.42	0.00	60%	d_{B_1} (80)	π_2^* (83)	MLCT
S_7	2.56	0.00	98%	d_{A_1} (79)	π_3^* (84)	MLCT
S_8	2.69	0.04	95%	d_{B_1} (80)	π_3^* (84)	MLCT
S_9	2.80	0.00	93%	d_{A_1} (79)	$d_{B_2}^*$ (86)	MC
S_{10}	2.96	0.08	40%	d_{A_2} (81)	π_3^* (84)	MLCT
S_{11}	3.10	0.03	35%	d_{A_2} (81)	$d_{A_1}^*$ (87)	MC
S_{12}	3.15	0.00	60%	d_{A_2} (81)	$d_{B_2}^*$ (86)	MC
S_{14}	3.77	0.00	60%	d_{B_1} (80)	$d_{A_1}^*$ (87)	MC
S_{15}	3.80	0.00	48%	d_{B_1} (80)	$d_{B_2}^*$ (86)	MC
S_{16}	3.83	0.00	90%	d_{A_1} (79)	π_4^* (85)	MLCT
S_{17}	3.83	0.00	98%	d_{B_1} (80)	π_4^* (85)	MLCT
S_{18}	3.83	0.00	59%	d_{A_2} (81)	π_4^* (85)	MLCT
S_{20}	4.07	0.01	57%	d_{A_1} (79)	$d_{A_1}^*$ (87)	MC
T_1	0.98	—	96%	d_{A_2} (81)	π_1^* (82)	MLCT
T_2	1.05	—	94%	d_{B_1} (80)	π_1^* (82)	MLCT
T_3	1.05	—	95%	d_{A_1} (79)	π_1^* (82)	MLCT
T_4	1.77	—	93%	d_{A_2} (81)	π_2^* (83)	MLCT
T_5	1.85	—	96%	d_{A_1} (79)	π_2^* (83)	MLCT
T_6	1.88	—	86%	d_{B_1} (80)	π_2^* (83)	MLCT
T_7	2.04	—	90%	d_{A_2} (81)	π_3^* (84)	MLCT
T_8	2.13	—	91%	d_{A_1} (79)	$d_{B_2}^*$ (86)	MC
T_9	2.14	—	97%	d_{A_1} (79)	π_3^* (84)	MLCT
T_{10}	2.18	—	95%	d_{B_1} (80)	π_3^* (84)	MLCT
T_{11}	2.25	—	90%	d_{A_2} (81)	$d_{B_2}^*$ (86)	MC
T_{12}	2.45	—	77%	d_{B_1} (80)	$d_{B_2}^*$ (86)	MC
T_{13}	2.74	—	68%	d_{A_2} (81)	$d_{A_1}^*$ (87)	MC
T_{14}	2.88	—	80%	d_{A_1} (79)	$d_{A_1}^*$ (87)	MC
T_{15}	2.92	—	81%	d_{B_1} (80)	$d_{A_1}^*$ (87)	MC
T_{16}	3.32	—	65%	d_{A_2} (81)	π_4^* (85)	MLCT
T_{20}	3.36	—	98%	d_{A_1} (79)	π_4^* (85)	MLCT
T_{22}	3.45	—	94%	d_{B_1} (80)	π_4^* (85)	MLCT

In Table S4 we compare the vertical excitation energies of the Fe complex in three different implicit solvents. The absorption spectra based on these data are shown in Figure 1 in the main text. The triplet energies are listed for completeness. Figure S5 shows the excitation energies and oscillator strengths from the table graphically and also presents the wave function overlaps between the states of the different calculations. Based on these overlaps, it can clearly be seen that the state characters of states below 3.3 eV are not affected by the choice of solvent. At higher energies, close-lying states might reorder due to small differential shifts. The only exception is found in the triplet calculations, where one high-energy triplet state shifts by more than 1 eV when changing the solvent.

Table S4: Results of vertical excitation calculations with different implicit solvents, showing the first 30 singlet excitation energies and oscillator strengths as well as the first 30 triplet excitation energies. Computed at the B3LYP*/mixed basis level of theory, using C-PCM solvation.

	— H_2O —			— DMSO —			— ACN —		
	E_{Singlet} (eV)	f_{osc}	E_{Triplet} (eV)	E_{Singlet} (eV)	f_{osc}	E_{Triplet} (eV)	E_{Singlet} (eV)	f_{osc}	E_{Triplet} (eV)
1	1.40	0.00	0.98	1.32	0.00	0.97	1.37	0.00	0.96
2	1.50	0.00	1.05	1.41	0.00	1.04	1.46	0.00	1.03
3	1.82	0.03	1.05	1.74	0.03	1.04	1.79	0.03	1.04
4	2.26	0.00	1.77	2.16	0.00	1.76	2.22	0.00	1.75
5	2.34	0.00	1.85	2.24	0.00	1.84	2.31	0.00	1.84
6	2.42	0.00	1.88	2.34	0.00	1.88	2.39	0.00	1.89
7	2.56	0.00	2.04	2.46	0.00	2.02	2.52	0.00	2.01
8	2.69	0.04	2.13	2.61	0.04	2.12	2.66	0.04	2.11
9	2.80	0.00	2.14	2.80	0.00	2.13	2.80	0.00	2.13
10	2.96	0.08	2.18	2.85	0.10	2.17	2.87	0.10	2.16
11	3.10	0.03	2.25	3.11	0.01	2.25	3.12	0.01	2.25
12	3.15	0.00	2.45	3.15	0.00	2.46	3.15	0.00	2.46
13	3.74	0.03	2.74	3.64	0.03	2.74	3.70	0.03	2.74
14	3.77	0.00	2.88	3.75	0.00	2.88	3.77	0.00	2.88
15	3.80	0.00	2.92	3.75	0.00	2.92	3.77	0.00	2.92
16	3.83	0.00	3.32	3.77	0.00	3.30	3.80	0.00	3.29
17	3.83	0.00	3.33	3.77	0.00	3.31	3.80	0.00	3.30
18	3.83	0.00	3.35	3.80	0.00	3.35	3.84	0.00	3.35
19	3.88	0.00	3.35	3.91	0.00	3.36	3.84	0.00	3.36
20	4.07	0.01	3.36	3.91	0.00	3.42	4.02	0.00	3.41
21	4.08	0.00	3.44	3.96	0.00	3.44	4.03	0.00	3.43
22	4.08	0.00	3.45	3.97	0.00	3.64	4.04	0.00	3.61
23	4.09	0.01	3.66	3.98	0.00	3.74	4.07	0.00	3.63
24	4.40	0.01	4.06	4.31	0.01	4.02	4.36	0.01	3.98
25	4.48	0.01	4.08	4.36	0.01	4.05	4.43	0.01	4.04
26	4.62	0.33	4.20	4.51	0.00	4.20	4.58	0.00	4.20
27	4.62	0.00	4.31	4.55	0.00	4.31	4.62	0.32	4.30
28	4.67	0.00	4.33	4.61	0.33	4.33	4.62	0.00	4.33
29	4.84	0.01	4.33	4.72	0.00	4.34	4.74	0.00	4.36
30	4.99	0.22	4.58	4.97	0.23	4.53	4.99	0.22	4.56

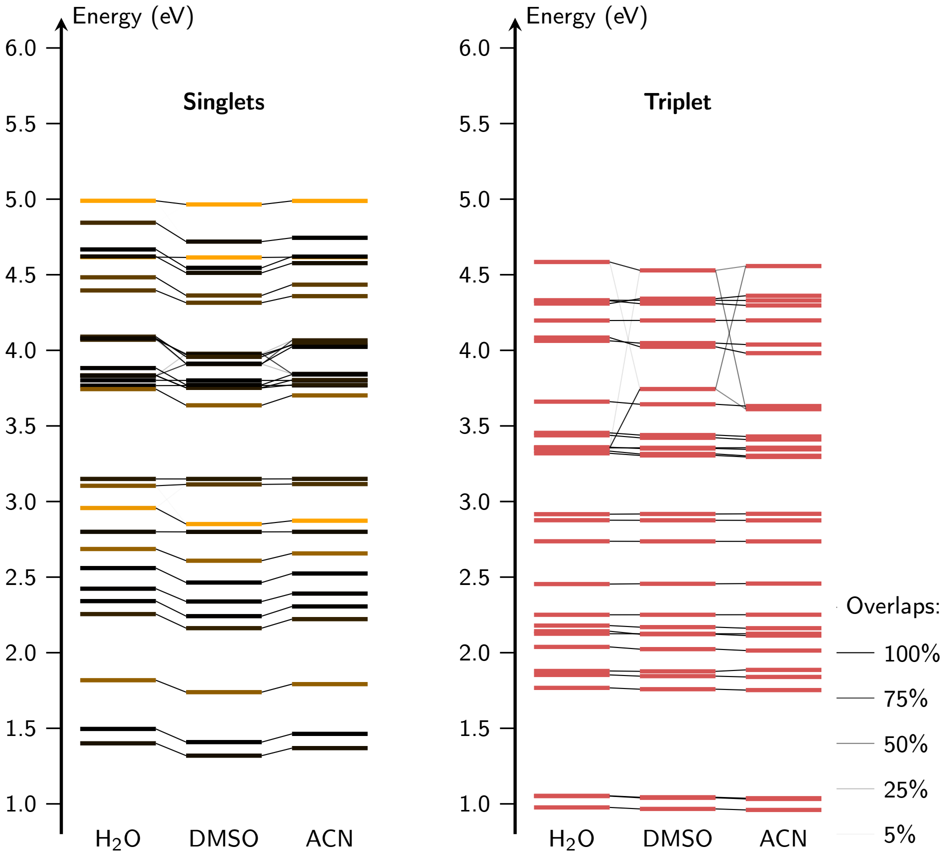

Figure S5: Vertical excitation energies from Table S4, oscillator strengths (black is dark, orange is bright, red is triplet), and wave function overlaps correlating the states of the different calculations.

S3.2 Charge transfer characters in the simulated absorption spectrum

Figure S6 presents the absorption spectrum (towards positive y-axis) and the mirrored density of states (towards negative y-axis) decomposed into different charge transfer contributions. The left panel show the spectra decomposed into contributions of MC character, and the right panel, into contributions of MLCT character, as defined above in Equation (6). As can be seen, the bright excited states within the lowest energy band of the absorption spectrum ($500 - 600$ nm) can be characterized as states of predominantly (80–90%) MLCT character (light blue).

Inspecting additionally the density of states (DOS) spectra is useful because is also shows the presence of dark states within a given energy region, as is often the case in transition metal complexes. From the DOS spectra, it can be seen that states of more than 80% MLCT character (blue colors, right panel) dominate the lowest energy band, however, few states of more than 80% MC character also exists at below 600 nm. These states of mainly MC character are most likely dark states, since they are contributing to the absorption spectrum. The second DOS energy band consist of states that show both high (blue colors) and low (red colors) MLCT contributions and a mixture of both MLCT and MC (yellow/green colors). Thus, the composition of the states in the second energy band is more complex than the lowest energy band. The third and very sharp band consists of states of neither MLCT or MC character (predominantly less than 20%), and thus involves other types of charge transfer character, e.g., ligand-centered.

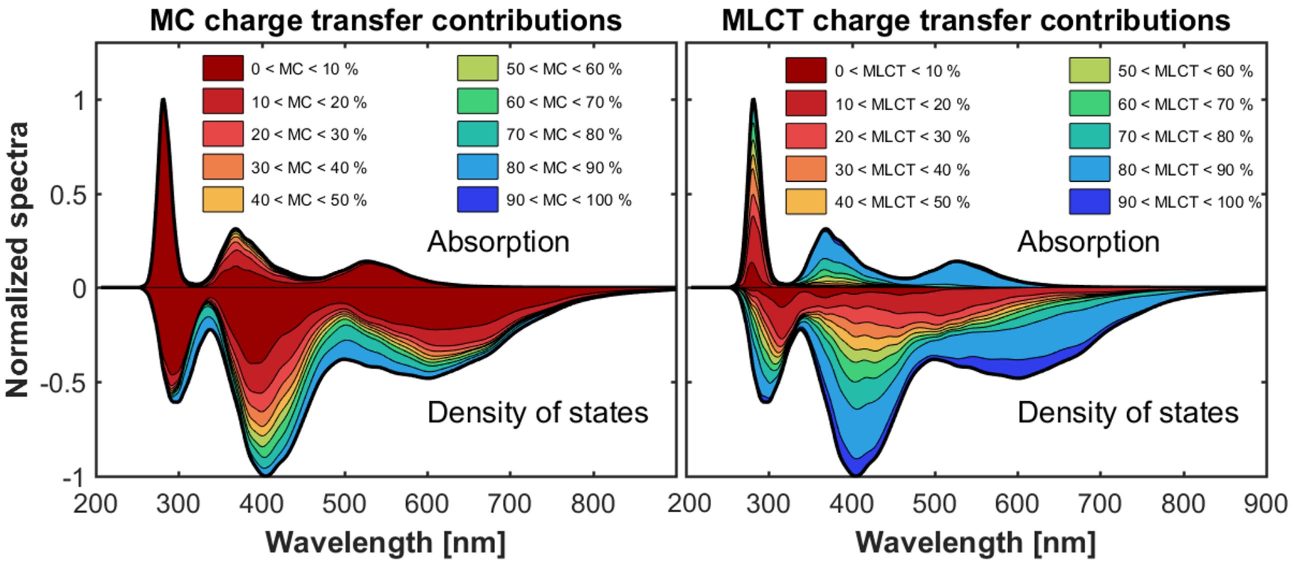

Figure S6: Simulated absorption spectrum (positive) and density of states (negative, including triplet states) decomposed into different charge transfer character contributions. Left panel, shows the decomposition of the spectra into contributions of MC character, and in the right panel, we decomposed into contributions of MLCT character.

S3.3 Charge transfer character from individual fragments

Figure S7 presents the individual time-dependent charge transfer descriptors for three fragments (Fe, $(CN)_4$, and bpy) to the electronic wave function, either using the total wave function (left), only the singlet contributions (middle), or only triplet contributions (right). The main contributions to the state character arise from charge transfer between the Fe→bpy (bright red), Fe→Fe (dark red), and CN→bpy fragments. Analysis of the character of only the singlet states shows almost exclusively contributions from Fe→bpy and CN→bpy directly upon excitation, which decreases to almost zero by the end of the 700 fs simulated time. As discussed previously for some transition metal complexes,[S48] often tightly bound cyanide or carbonyl ligands form an electronic unit with the metal atom due to very covalent bonds. In such situations, it is expedient to treat the metal and those ligands as one fragment for charge transfer analyses, as we have done in the main text.

Consequently, the Fe→bpy and CN→bpy can be summed up to yield the MLCT contribution, showing that the electronic dynamics in the singlet states involves only MLCT states (middle panel). In contrast, analysis of only the triplets shows a mixture of Fe→bpy, CN→bpy, and Fe→Fe charge transfer contributions, and thus a mixture of both MLCT and MC characters that grow in with almost equal contributions. The remaining contributions are less than 10%, and thus only minimal LMCT and LC character is observed.

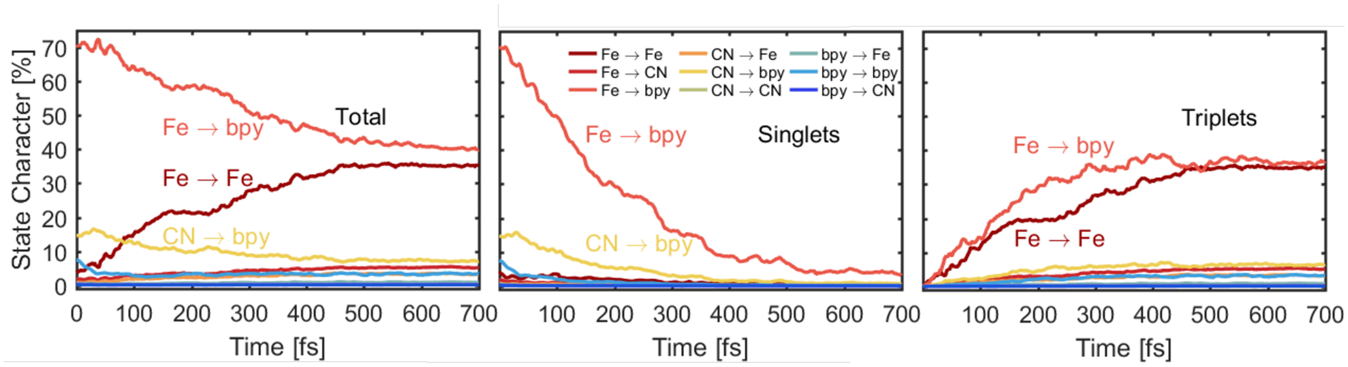

Figure S7: Time-dependent composition of the electronic wave function in terms of charge transfer character. LEFT: The contributions of charge transfer character of the total electronic wave function. MIDDLE: the charge transfer composition of only the singlet states. RIGHT: the contributions considering only the triplet states.

S3.4 Comparison of averaged goemetries from explicit solvation and optimized geometries from implicit solvation

In Table S5, we compare characteristic bond lengths from the SHARC dynamics simulations with bond lengths obtained from optimized geometries in implicit solvent. The bond lengths from the SHARC simulations are total averages over all trajectories, all time steps, and all spin, weighted by the CT state character to obtain average results for the ground state (GS), the MLCT states, and the MC states. The bond lengths from optimized geometries were obtained as described in Section S1.1; the Cartesian coordinates are given below in XYZ format (Section S4). Note that there exist several MC and MLCT states with slightly different minima; here we only look at one ^{3}MC and one ^{3}MLCT minimum each, optimized from suitable starting geometries.

The table considers three aspects: (i) how bond lengths depend on CT character in the dynamics, (ii) how bond lengths differ between explicit and implicit solvation, (iii) and how bond lengths differ between implicit solvents (ACN, DMSO, water). (i) Comparing the results from the SHARC trajectories in different states provides the same findings as Figure 3 in the main text. The Fe–X bonds do not change strongly in the MLCT state compared to the ground state, whereas the MC state induces strong stretching of Fe–N and equatorial Fe–C bonds. On the contrary, the MLCT state induces changes in the bipyridine bond lengths. (ii) Explicit and implicit calculations show the same trends in the geometric changes induced by the different states. However, there are numeric differences, in particular for Fe–X and cyanide C–N bond lengths, which arise because implicit solvation does not consider hydrogen bonding. (iii) The table also shows clearly that implicit solvation produces almost identical geometries in different solvents, again because hydrogen bonding is not described.

Table S5: Bond lengths from average structures (weighted by state character) extracted from the dynamics simulations (SHARC). For comparison, bond lengths of the optimized structures (opt) in different states and with different implicit solvent. Optimized with Gaussian, (TD-)B3LYP*/mixed basis, using IEFPCM implicit solvation treatment. See Figure S3 for the atom labels.

State	Solvent	r_{FeN} (Å)	$r_{\mathrm{FeC_e}}$ (Å)	$r_{\mathrm{FeN_a}}$ (Å)	$r_{\mathrm{C_eN_e}}$ (Å)	$r_{\mathrm{C_aN_a}}$ (Å)	$r_{\mathrm{N_1C_2}}$ (Å)	$r_{\mathrm{N_1C_6}}$ (Å)	$r_{\mathrm{C_2C_2}}$ (Å)	$r_{\mathrm{C_2C_3}}$ (Å)	$r_{\mathrm{C_3C_4}}$ (Å)	$r_{\mathrm{C_4C_5}}$ (Å)	$r_{\mathrm{C_5C_6}}$ (Å)
^{1}GS	H_2O (SHARC)	2.041	1.904	1.931	1.156	1.145	1.340	1.352	1.478	1.389	1.384	1.390	1.396
^{1}GS	ACN (opt)	2.021	1.942	1.973	1.175	1.174	1.343	1.356	1.473	1.393	1.398	1.393	1.403
^{1}GS	DMSO (opt)	2.022	1.941	1.972	1.175	1.174	1.343	1.356	1.473	1.393	1.398	1.393	1.403
^{1}GS	H_2O (opt)	2.022	1.941	1.972	1.175	1.174	1.343	1.356	1.474	1.393	1.398	1.393	1.403
$^{1/3}$MLCT	H_2O (SHARC)	2.076	1.918	1.943	1.149	1.142	1.337	1.380	1.442	1.390	1.407	1.381	1.419
^{3}MLCT	ACN (opt)	2.014	1.941	1.967	1.169	1.169	1.348	1.393	1.421	1.381	1.427	1.375	1.428
^{3}MLCT	DMSO (opt)	2.013	1.942	1.967	1.169	1.169	1.348	1.393	1.421	1.381	1.427	1.375	1.427
^{3}MLCT	H_2O (opt)	2.012	1.942	1.967	1.169	1.169	1.349	1.393	1.421	1.381	1.427	1.375	1.427
$^{1/3}$MC	H_2O (SHARC)	2.264	2.031	1.942	1.129	1.136	1.336	1.345	1.484	1.385	1.386	1.390	1.397
^{3}MC	ACN (opt)	2.346	2.076	1.971	1.173	1.174	1.335	1.345	1.489	1.396	1.397	1.394	1.406
^{3}MC	DMSO (opt)	2.344	2.076	1.971	1.173	1.174	1.336	1.345	1.489	1.396	1.397	1.394	1.406
^{3}MC	H_2O (opt)	2.336	2.078	1.970	1.173	1.174	1.336	1.345	1.489	1.396	1.397	1.394	1.406

S3.5 Additional RDFs

This section presents additional RDFs for selected solute–solvent atom pairs. Each plot shows in the left panel the RDFs averaged over different time spans, as in Figure 4. In the right panel, each plot shows the difference RDFs at each time step, relative to the average ground state RDF. The insets show the first V_1 component from the SVD analysis along with kinetic fits. In general, solute–H RDFs show less noise than the corresponding solute–O RDFs, because there are twice as many H atoms, leading to better statistics, and the H atoms oscillate much faster than the O atoms, leading to better sampling. However, the trends from solute–H and solute–O RDFs are the same.

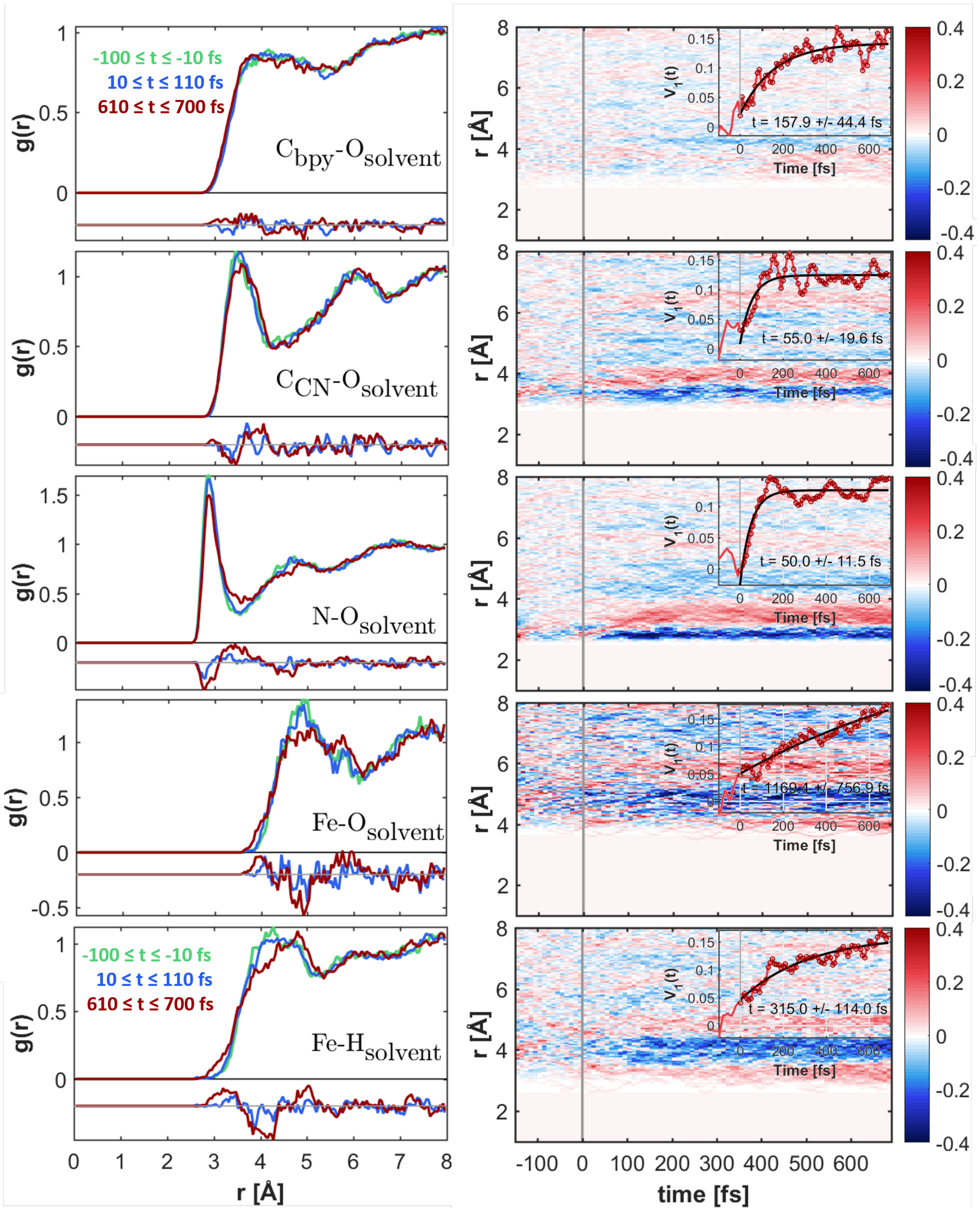

Figure S8: Additional solute-solvent radial distribution Functions (RDFs).

S3.6 SVD of RDFs

The figures presented in this section presents the main components from a singular value decomposition (SVD) $\Delta g(r,t) = \sum_i U_i(r) \cdot s_i \cdot V_i(t)$ of the 2D difference RDFs $\Delta g(r,t)$. Figure S9 shows the components from the difference RDFs presented above in Figure S8 (second column) and Figure S10 shows the SVD components from the difference RDFs presented in the main text in Figure 4 (right column). Each color corresponds to a given set i consisting of $U_i(r)$, s_i, and $V_i(t)$. The first column in Figures S10 and S9 presents the weighted $U_i(r)$ components for the 3 components ($i = 1, 2, 3$) with the largest singular values s_i. These $U_i(r)$ components describe the most important spatial shapes of the difference RDFs. The second column presents the corresponding weighted $V_i(t)$ components, describing the time evolution in the difference RDFs. The last column gives the first 10 singular values s_i, describing the weight of each component in descending order.

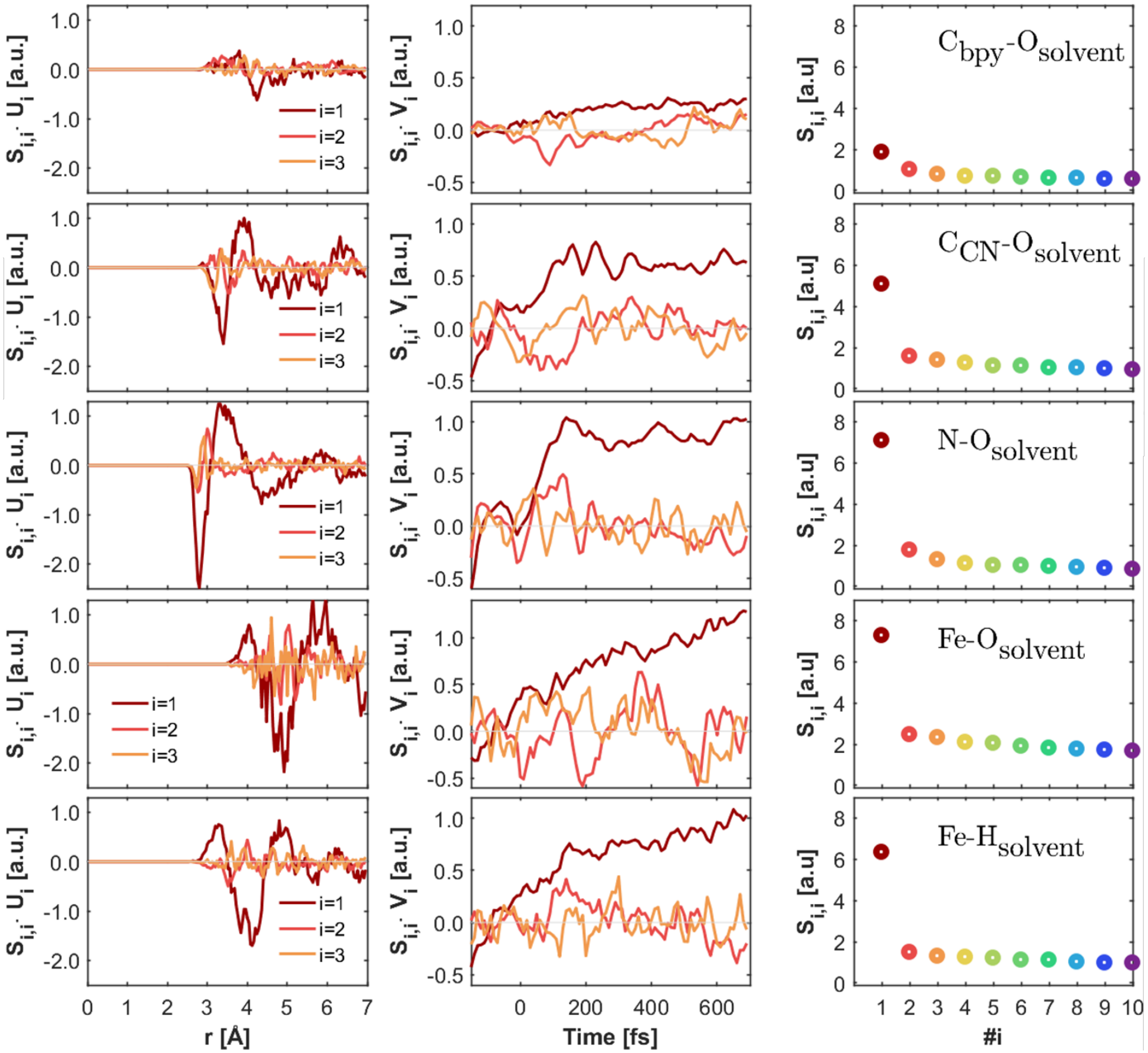

Figure S9: Components U_i,V_i,$S_{i,i}$ from an SVD of the additional difference RDFs $\Delta g(r,t)$ presented in Section S3.5, Figure S8.

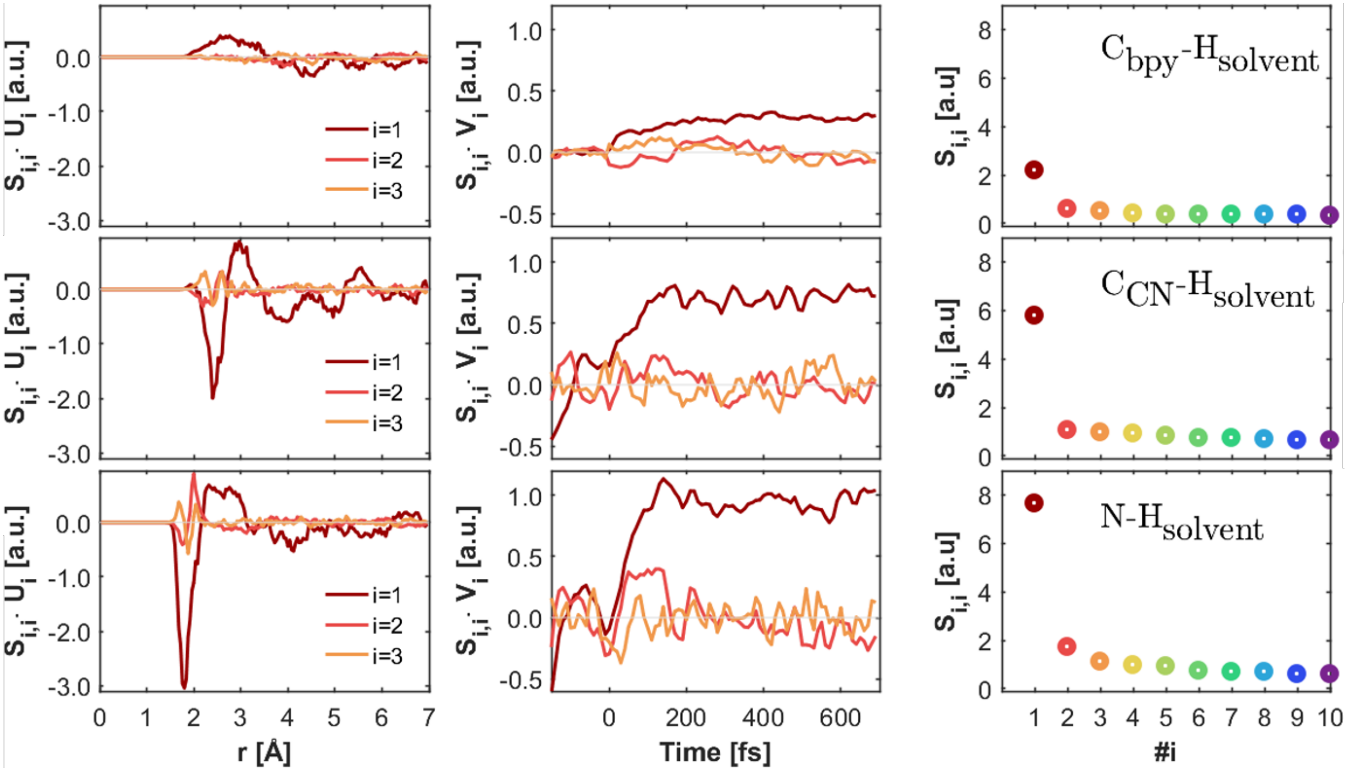

Figure S10: Components U_i,V_i,$S_{i,i}$ from an SVD of the difference RDFs $\Delta g(r,t)$ presented in the main text in Figure 4.

S3.7 Charge transfer weighted RDFs

Figures S11 and S12 shows the charge transfer weighted RDFs for the cyanide nitrogen atoms relative to water H and O atoms. In each figure, panel (a) shows the time-averaged RDFs weighted according to mainly MLCT (light blue) or mainly MC (dark red) charge transfer character. Below (offset by -0.2) is the average difference (dark blue) showing a small dip at ~ 1.9 Å or ~ 2.7 Å followed by a broad positive feature at longer distances. Panel (b) shows the differences (MLCT$-$MC) for each time step in the excited state, since the ground state is by definition not included in the charge transfer analysis. The charge transfer weighted RDFs shows only small differences between MLCT and MC character (see panels (c) and (d)), however, with a significant trend that MLCT states have slightly weaker interactions between cyanides and water observed from a small decrease in peak height around 1.9 Å or 2.7 Å and slight broadening at 2–3 Å or 3–4 Å compared to the MC state. The difference plot in panel (b) shows that the small dip around 1.9 Å or 2.7 Å (blue features) remains throughout the dynamics.

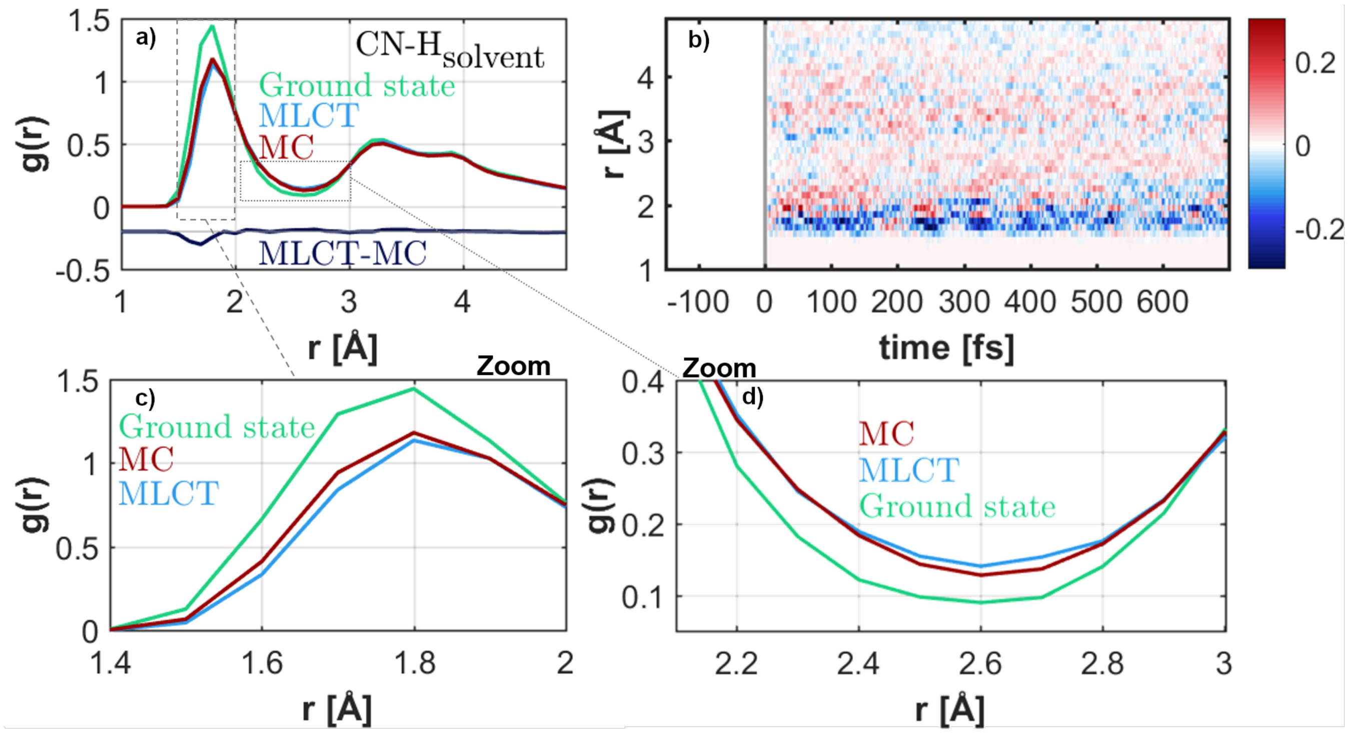

Figure S11: (a) Comparison of averaged N–H RDFs weighted according to MLCT (light blue) or MC (dark red) character and their difference (dark blue, offset by -0.2). (b) MLCT-MC weighted RDF differences for each time step in the excited state. (c) and (d) Zooms of the most relevant parts of (a).

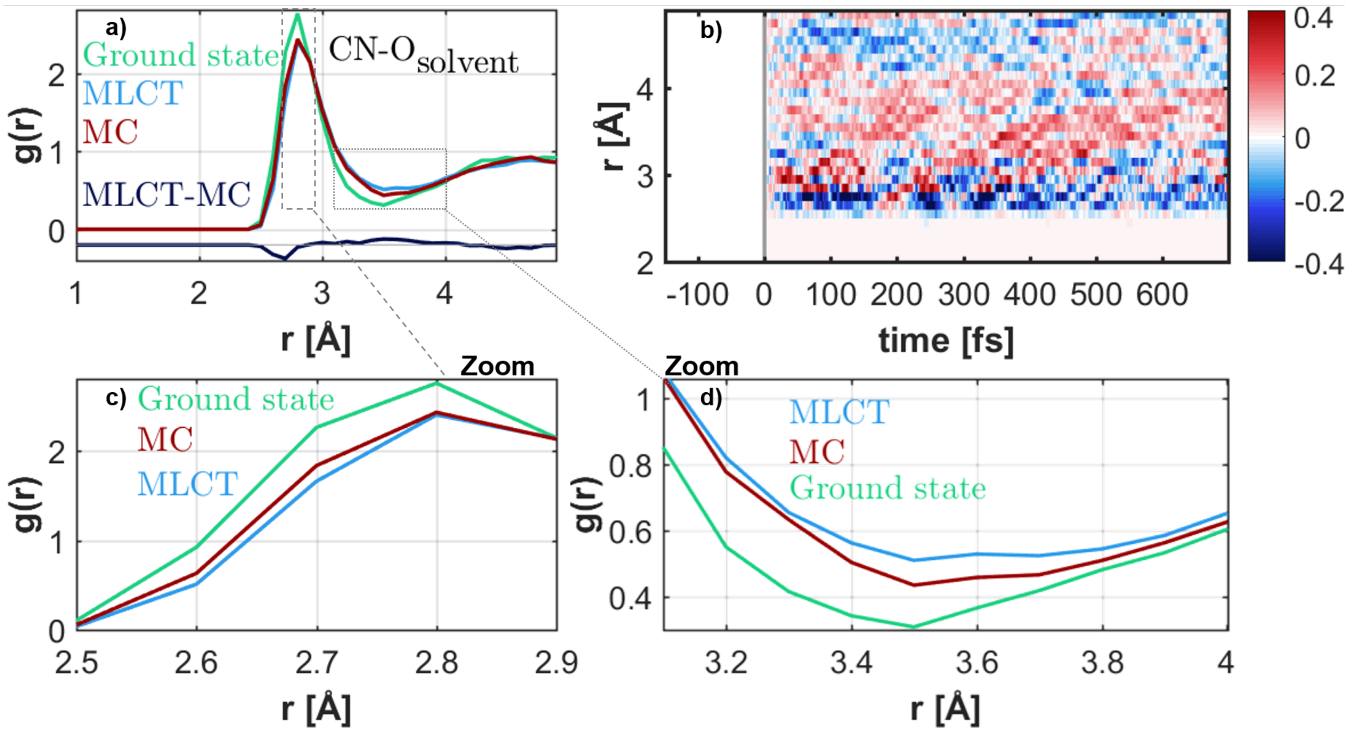

Figure S12: (a) Comparison of averaged N–O RDFs weighted according to MLCT (light blue) or MC (dark red) character and their difference (dark blue, offset by −0.2). (b) MLCT-MC weighted RDF differences for each time step in the excited state. (c) and (d) Zooms of the most relevant parts of (a).

S3.8 RDFs of equatorial versus axial cyanides

Figures S13 and S14 shows the N–H and N–O solute-solvent RDFs from the axial cyanides (top), equatorial cyanides (middle), and the differences between the two (bottom). In each of the two figures, the two top left plots shows the averaged RDFs over selected time steps in the ground state (green), early excited state times (blue), and late excited state times (dark red). The two top right plots shows the excited–ground state differences for each time step relative to the average ground state. The insets show the first temporal component, $V_1(t)$ from an SVD of the time-dependent differences and monoexponential fits. Overall, the shapes are very similar and resembles the overall trends observed in Figure S9 for the N-$O_{solvent}$ RDFs. We mainly observe a general weakening of the interaction with the nearest solvent (broadening and decrease in peak height at roughly 1.9 Å or 2.8 Å) on a 40–60 fs time scale (see insets), although with indications of a slightly faster response around the axial than the equatorial cyanides. The bottom left plot compares the time-averaged RDFs of axial (pink) and equatorial (purple) cyanides and the average difference (red, offset by −0.2). From the average RDFs, we find that the solvation structure around the axial and equatorial are similar, but with a slightly stronger interaction of the axial cyanides with the nearest solvent. The bottom right plot shows the axial–equatorial differences for each time step in both the ground and excited state dynamics simulations. The inset shows the first temporal component, $V_1(t)$, which indicates that the differences between axial and equatorial cyanides are static, despite the bond elongations found for the equatorial cyanides in the dynamics.

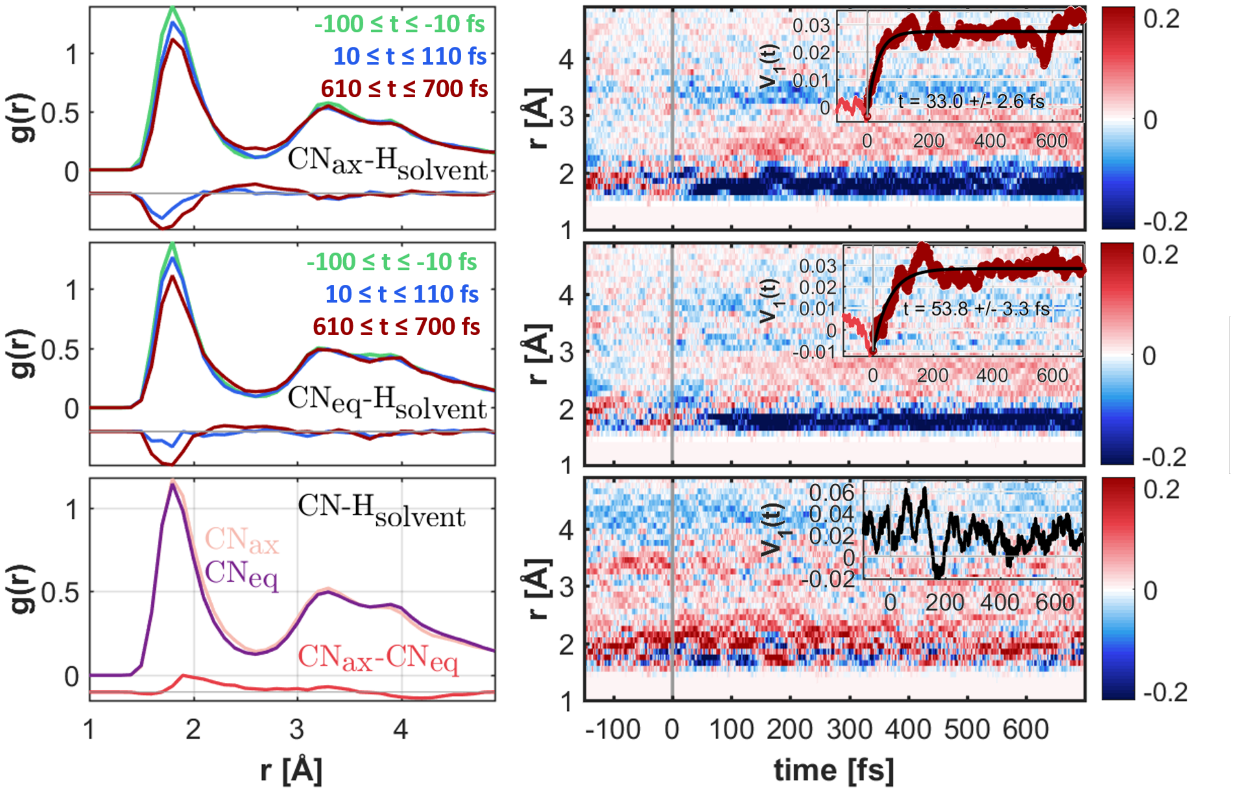

Figure S13: N–H solute-solvent radial distribution Functions (RDFs) from axial (ax) or equatorial (eq) cyanide nitrogens. Top and middle plots shows the average RDFs at selected time ranges and differences to the average ground state. Bottom plot shows the time-averaged RDFs for the axial (pink), equatorial (purple) cyanides and the difference between them below (red, offset by −0.2). Insets show the first temporal component, $V_1(t)$ from an SVD.

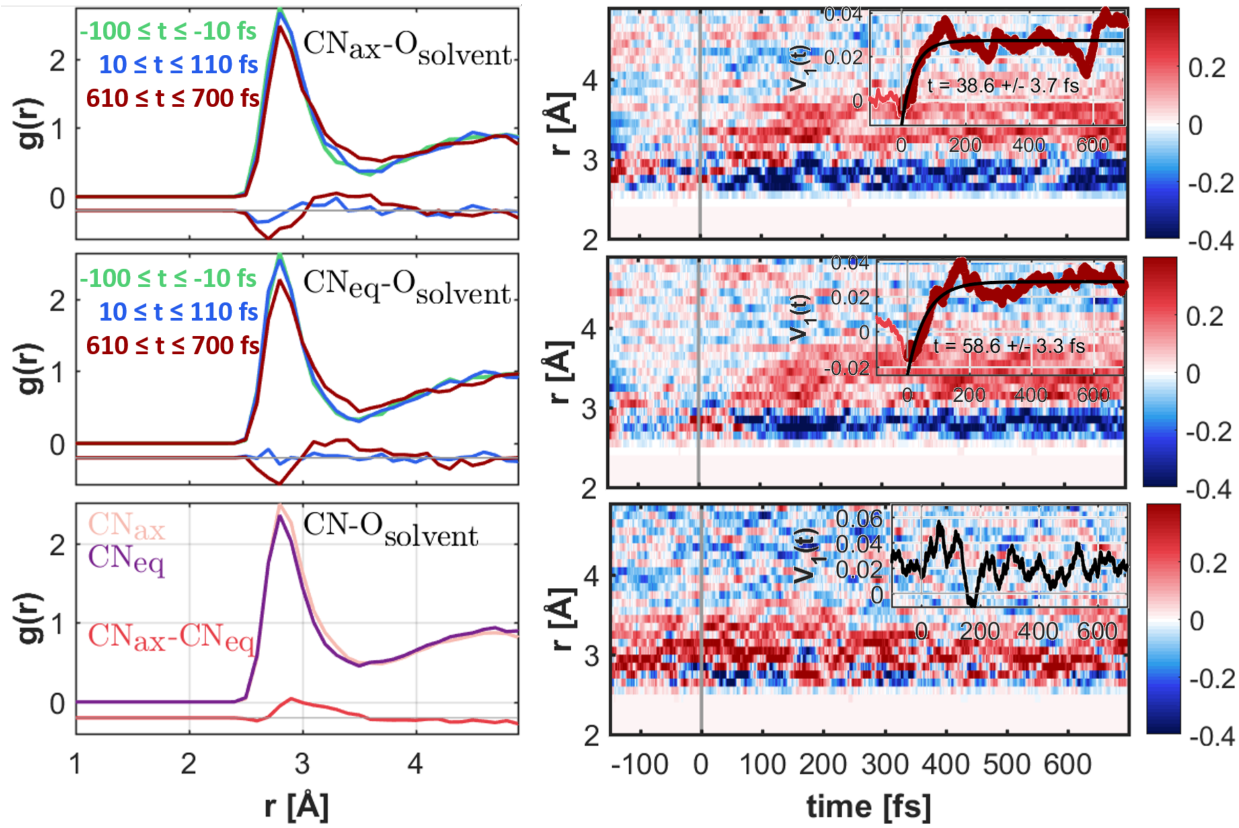

Figure S14: N–O solute-solvent radial distribution Functions (RDFs) from axial (ax) or equatorial (eq) cyanide nitrogens. Top and middle plots shows the average RDFs at selected time ranges and differences to the average ground state. Bottom plot shows the time-averaged RDFs for the axial (pink), equatorial (purple) cyanides and the difference between them below (red, offset by -0.2). Insets show the first temporal component, $V_1(t)$ from an SVD.

S3.9 Hydrogen bonds

The strong cyanide–water interaction observed in the RDFs inspired us to extract the number of H-bonds around the cyanide ligands for each time step of the simulations. A hydrogen bond was defined by occurrences of water H-atoms within an N-O distance of 2.45$\leq R_{\text{N-O}} \leq$3.55 Å and angle of 135$\leq \alpha_{\text{NHO}} \leq 180°$. These criteria were chosen based on the main peak in the angular-resolved radial distribution function (see below).

The left panel of Figure S15 shows the temporal evolution of the number of hydrogen bonds per cyanide, as obtained with the above H-bond thresholds (total in black, axial cyanides in purple, equatorial cyanides in green), along with a kinetic fit giving a time decay constant of about 113 fs. The residual between data and fit is shown below (with an offset of 3.1).

However, the noise level of the data is rather high, and very sensitive to the chosen distance and angle thresholds. To investigate the effect on the extracted time scale, we extracted the number of H bonds using different thresholds for the maximum distance (i.e., using 2.45$\leq R_{\text{N-O}} \leq R_{\max}$) and for the minimum angle (using $\alpha_{\min} \leq \alpha_{\text{NHO}} \leq 180°$). The middle and right panels of Figure S15 show the results of the distance and angle scans. The corresponding time constants are given in Table S6, which vary between 40–130 fs for different choices of thresholds. Due to this degree of arbitrariness, in the main text we instead employ singular value decomposition of the respective RDFs to extract the time constants of solvation dynamics.

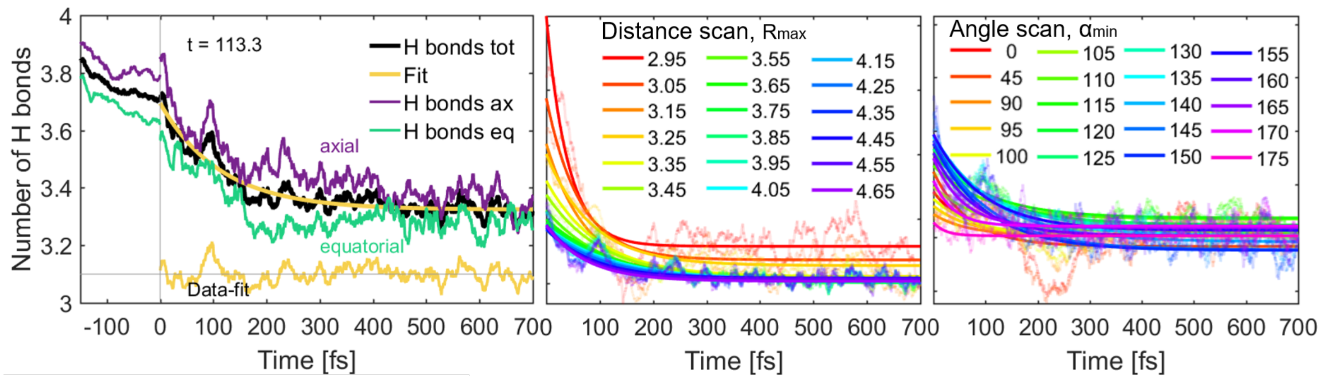

Figure S15: LEFT: Time-dependent nearest number of H-bonds per cyanide ligands (black), only the axial (purple) or only the equatorial (green) cyanides. A monoexponential fit (yellow) of the total data gives a time decay constant of $\sim$ 113 fs and the residual is shown below with an offset of 3.1. MIDDLE: Results of the H-bond dynamics and fits for various choices of the maximum threshold $R_{\max}$, defined from the solute-solvent N-O distances, $R_{\text{N-O}}$. RIGHT: Results of the H-bond dynamics and fits for various choices of the minimum threshold $\alpha_{\min}$, defined from the solute-solvent N–H-O angle α_{NHO}. The y-axis of both scan-plots are arbitrary since the data was re-scaled to the last point at $t = 700$ fs.

Table S6: Monoexponential time constants fitted from the data in Figure S15.

r_{max} (Å)	τ_{decay} (fs)[a]	α_{min} (°)	τ_{decay} (fs)[b]
2.95	40.3	0	61.8
3.05	62.5	45	68.4
3.15	74.6	90	66.0
3.25	85.1	95	70.8
3.35	97.2	100	80.5
3.45	104.4	105	95.7
3.55	113.3	110	98.1
3.65	129.8	115	103.1
3.75	127.3	120	112.1
3.85	129.4	125	116.1
3.95	121.2	130	115.9
4.05	117.6	135	113.3
4.15	111.7	140	116.7
4.25	108.4	145	107.5
4.35	91.4	150	100.8
4.45	83.3	155	94.1
4.55	74.7	160	79.8
4.65	75.7	165	55.9
4.75	76.8	170	38.3

[a] Fitted using $r_{\mathrm{min}} = 2.45$ Å, $\alpha_{\mathrm{min}} = 135°$, $\alpha_{\mathrm{max}} = 180°$, and the given r_{max}.
[b] Fitted using $r_{\mathrm{min}} = 2.45$ Å, $r_{\mathrm{max}} = 3.55$ Å, $\alpha_{\mathrm{max}} = 180°$, and the given α_{min}.

S3.10 Angle-resolved RDFs

The time-dependent solvent distribution around the cyanides in the excited-state trajectories was quantified with angle-resolved RDFs (ARDFs), as shown in Figures S16 and S17. Each column in the figures shows solute-solvent N–O (or N–H) distances, $R_{\text{N-O}}$, relative to an angle α formed between the different solute (C or N) and solvent (O or H) atoms. The top row presents the normalized ARDFs, averaged over the ground state trajectories (–150 to 0 fs), and the remaining rows present the difference ARDFs relative to the ground state directly following excitation (0–50 fs), at early times (100–150 fs) or later times (650–700 fs). Each of the left halves shows the ARDFs for the axial cyanides, and the right halves for the equatorial cyanides. Note that 99 trajectories are insufficient to converge the ARDFs. Hence, we regard these findings from them as speculative.

The left column of Figures S16 and S17 shows the donor–acceptor distance $R_{\text{N-O}}$ versus the donor–hydrogen–acceptor angle $\alpha_{\text{N-H-O}}$, which are the main parameters to identify hydrogen bonds. We find a sharp peak located at 3 Å and 135–180° that represents the H bonds. A weaker peak at the same distance and at approximately 60-70° (smaller yellow spots) arises from the second hydrogen atom bonded to the donor oxygen. Further structure arising from the second solvation shell can be seen at 135–180° and distances above 4 Å. The time-dependent difference plots show that after excitation the main peak is reduced (as observed in the RDFs). It appears that this reduction is accompanied by a reorientation of the water molecules, as seen by the decrease of density for 150–180° and an increase for 150–90°.

The middle column of Figures S16 and S17 shows the dependence of solvent density of the C–N–O angle and thus describes where the water molecules are relative to the cyanide. The right column shows the location of hydrogen atoms analogously. Both plots indicate that water molecules are located in a half sphere around the cyanide N atoms, mostly located "side-on" (O–N and N–C bonds roughly perpendicular) or "end-on" (water molecule roughly on the C–N bond axis). The time-dependent difference plots are very noisy and do not allow extracting trends. Furthermore, only little difference is observed in the dynamics of axial and equatorial cyanides.

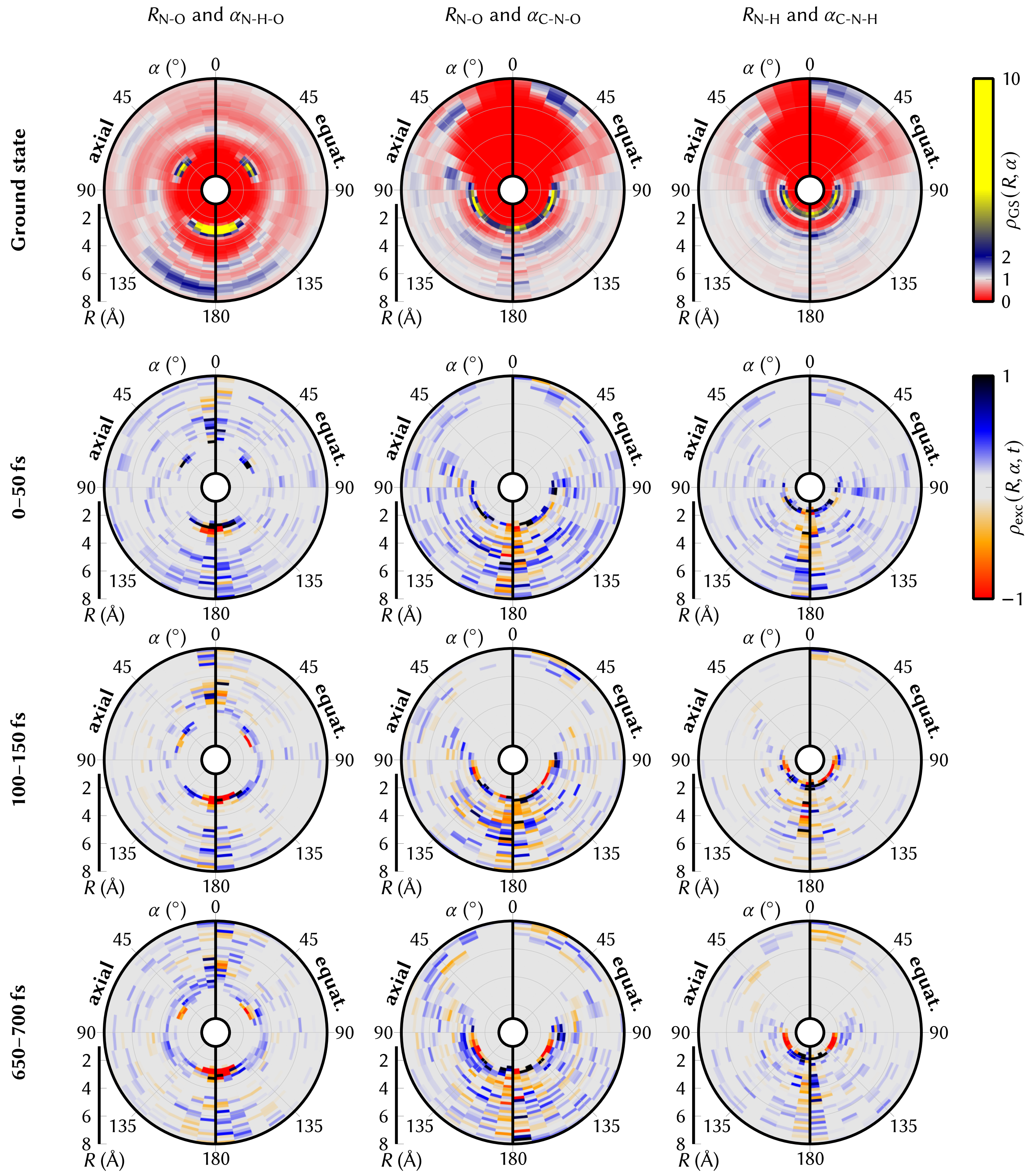

Figure S16: Angular-radial distribution functions (ARDF) of water relative to cyanide ligands for $[Fe(CN)_4(bpy)]^{2-}$. The left halves of all plots correspond to the axial cyanides, the right halves to the equatorial cyanides. The left column plots the donor–acceptor distance $R_{\text{N-O}}$ versus the donor–hydrogen–acceptor angle $\alpha_{\text{N-H-O}}$, which are the main parameters to identify hydrogen bonds (present around $R_{\text{N-O}} \approx 3.0$ Å and $\alpha_{\text{N-H-O}} > 135°$). The second column plots the same distance but the C=N–O angle, therefore illustrating the position of oxygen relative to the cyanides. The third column illustrates the position of hydrogen relative to the cyanides. The first row gives the normalized ARDF averaged between -150 and 0 fs. The latter rows give the difference ARDFs (relative to ground state) at 0–50 fs, 100–150 fs, and 650–700 fs.

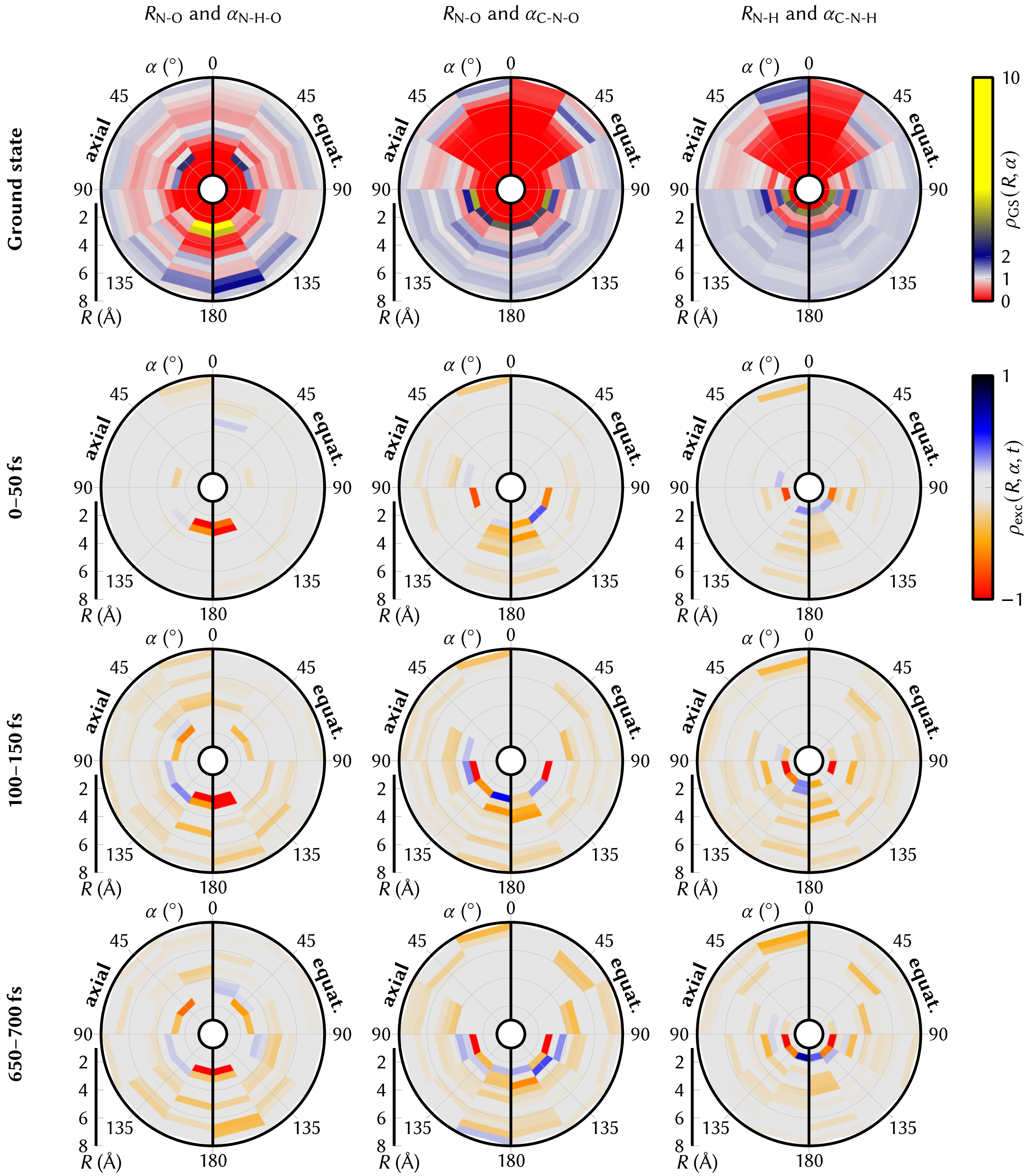

Figure S17: Angular-radial distribution functions (ARDF) of water relative to cyanide ligands for $[Fe(CN)_4(bpy)]^{2-}$, as in Figure S16 but with larger histogram bins to reduce noise.

S3.11 X-ray solution scattering signals

Figure S18 shows a one-dimensional representation of the X-ray scattering signals shown in Figure 5 of the main text. Panel (a) shows the time-averaged signals of solute, cross-term, and solvent scattering. Panels (b) to (d) show the (color-coded) time dependence of these three scattering contributions. The figure is primarily intended to enable a direct comparison of the scattering signal strength of the three contributions.

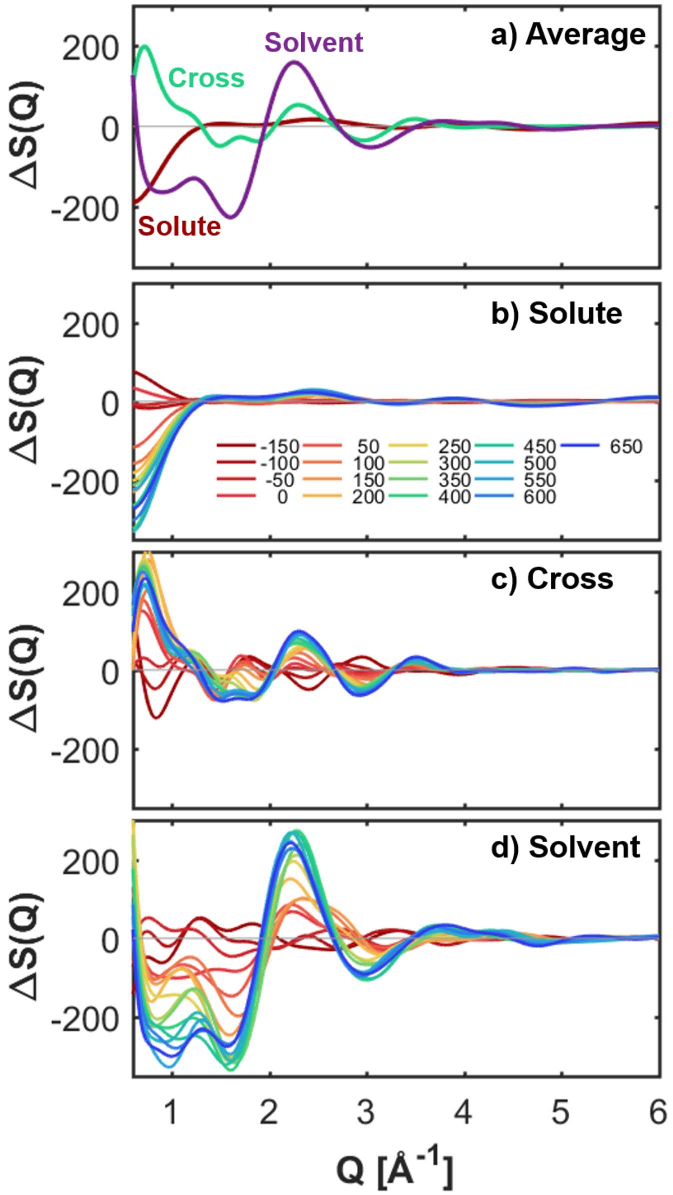

Figure S18: One-dimensional representation of the X-ray scattering signals shown in Figure 5 of the main text. Panel (a) shows the time-averaged signals of solute, cross-term, and solvent scattering. Panels (b) to (d) show the (color-coded) time dependence of these three scattering contributions. Note that all four panels use the same vertical axis to enable direct comparison.

Note that the three different contributions in Figures 5, 6, and S18 are calculated from RDFs with slightly different normalization. The RDFs relevant for the solute and cross term scattering used the entire simulation system of 5412 water molecules. This corresponds to a concentration of about 10 mM. The solvent–solvent RDFs instead were computed only for the 685 water molecules closest to the iron complex, because only those water molecules are properly surrounded by other water

molecules up to a sufficient distance. This would correspond to a quite high concentration of about 80 mM.

To directly compare to a given future experiment would hence require an appropriate rescaling of each contribution depending on the concentration used for the experiment. However, in case the experimental signals were scaled according to 1 Liquid Unit Cell (LUC),[S49] i.e., to one solute molecule per number of solvent molecules, according to the given concentration used at the measurements, then only the solvent contribution needs rescaling. This is observed from Equation S10, where the solute and cross terms scale with the number of solute atoms, N_u (thus independent of concentration when scaled to 1 LUC), whereas the solvent term scales with the number of solvent atoms, N_v.

We note that, additionally, the solute and cross-term contributions would depend on the fraction of excited molecules, which is also not considered in the relevant figures in this work.

S3.12 Vibrational analysis using implicit solvation

Simulated steady-state IR spectra obtained from frequency calculations in implicit solvation are shown in Figure S19 and corresponding frequencies in Table S7. Similar to the optimized geometries, the vibrational frequencies and IR intensities differ only marginally between different implicit solvents (frequencies differ by at most $5\,\mathrm{cm}^{-1}$). On the contrary, the IR spectra differ significantly between the different electronic states. Differences are rather large in the CH stretch region above $3000\,\mathrm{cm}^{-1}$, but the overall intensities in this regions are too low to distinguish the states.

The CN stretch region (around $2200\,\mathrm{cm}^{-1}$) shows the expected up-shift in the MLCT state, arising from a reduction in electron density on the Fe atom, which reduces π backbonding to the CN ligands, which in turn lowers the occupation of the antibonding π^* orbitals of the cyanides and makes the CN bond stronger. Given sufficient time resolution, time-resolved infrared spectroscopy could in principle observe the transient MLCT state, although it will be difficult to distinguish the ground state and MC state.

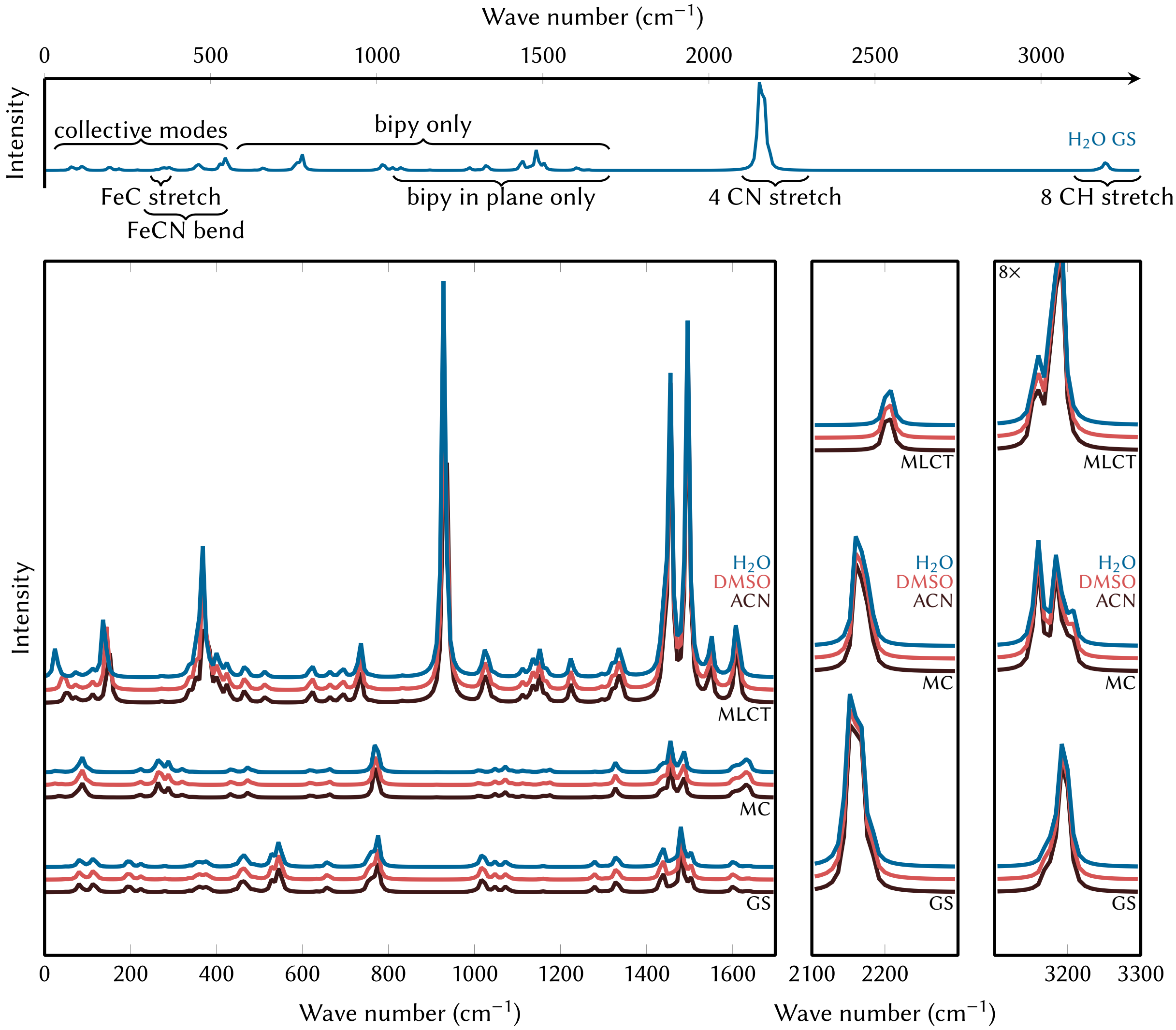

Figure S19: Simulated infrared spectra of the Fe complex in water using implicit solvation. Computed with B3LYP*/mixed basis and IEFPCM implicit solvation. Differences between solvents are minimal at this level, whereas differences between electronic states are very large.

At lower frequencies (below 1700 cm^{-1}), the spectra of ground state and MC state are relatively similar, in particular modes corresponding to vibrations of the bipy ligand. The MLCT state spectrum differs strongly from the other states' spectra due to featuring effectively a bipy^{-} anion with very different vibrations. The large dipole moment of the MLCT state also leads to much more intense bands than in the other states. At frequencies below 500 cm^{-1}, differences in the collective modes can be seen, but this region has low intensity and is typically more difficult to measure. The important Fe–X stretch modes are found in this low-frequency region, where their frequencies depend on the electronic state. From the frequency calculations, we expect that especially the Fe–N stretch modes shift to lower frequencies in the MC state.

Table S7: Vibrational frequencies from frequency calculations using implicit solvation, classified in C_{2v} symmetry.

Normal mode	GS (cm^{-1})	MLCT (cm^{-1})	MC (cm^{-1})	Normal mode	GS (cm^{-1})	MLCT (cm^{-1})	MC (cm^{-1})
$1a_1$	80	75	61	$1b_2$	77	61	38
$2a_1$	108	110	87	$2b_2$	116	119	79
$3a_1$	192	201	129	$3b_2$	219	161	97
$4a_1$	274	280	221	$4b_2$	368	360	253
$5a_1$	325	342	263	$5b_2$	411	378	280
$6a_1$	366	368	314	$6b_2$	469	427	454
$7a_1$	368	378	337	$7b_2$	523	516	479
$8a_1$	457	403	349	$8b_2$	637	622	625
$9a_1$	538	499	469	$9b_2$	665	659	667
$10a_1$	661	671	650	$10b_2$	1025	933	1018
$11a_1$	786	768	785	$11b_2$	1056	1033	1055
$12a_1$	1029	1028	1023	$12b_2$	1092	1054	1096
$13a_1$	1081	1040	1079	$13b_2$	1140	1148	1134
$14a_1$	1124	1120	1121	$14b_2$	1169	1161	1168
$15a_1$	1180	1177	1185	$15b_2$	1312	1302	1316
$16a_1$	1292	1235	1292	$16b_2$	1334	1332	1333
$17a_1$	1330	1345	1323	$17b_2$	1473	1448	1469
$18a_1$	1341	1383	1348	$18b_2$	1496	1479	1500
$19a_1$	1450	1462	1450	$19b_2$	1622	1509	1635
$20a_1$	1518	1545	1519	$20b_2$	1653	1623	1651
$21a_1$	1607	1568	1619	$21b_2$	2176	2239	2202
$22a_1$	1651	1599	1644	$22b_2$	3194	3178	3183
$23a_1$	2181	2239	2201	$23b_2$	3206	3197	3194
$24a_1$	2203	2241	2208	$24b_2$	3215	3202	3210
$25a_1$	3195	3180	3184	$25b_2$	3218	3215	3219
$26a_1$	3207	3201	3194				
$27a_1$	3217	3208	3211				
$28a_1$	3227	3216	3230				
$1b_1$	48	40	34	$1a_2$	38	40	17
$2b_1$	84	78	71	$2a_2$	84	89	79
$3b_1$	96	98	88	$3a_2$	116	102	82
$4b_1$	196	181	157	$4a_2$	263	236	241
$5b_1$	350	337	316	$5a_2$	347	331	294
$6b_1$	383	362	358	$6a_2$	471	425	423
$7b_1$	446	417	430	$7a_2$	474	448	432
$8b_1$	490	440	438	$8a_2$	579	539	577
$9b_1$	536	478	467	$9a_2$	767	730	758
$10b_1$	765	694	771	$10a_2$	845	770	854
$11b_1$	782	743	779	$11a_2$	922	844	922
$12b_1$	918	839	922	$12a_2$	1009	986	1015
$13b_1$	1010	991	1014	$13a_2$	1036	1000	1039
$14b_1$	1037	1000	1041				
$15b_1$	2190	2235	2192				

S3.13 Solvent effects on electronic energies

In Figure S20, we show how the excitation energies (based on one arbitrarily selected QM/MM geometry at $t = 0$) change when some or all of the solvent point charges are turned off. We divide the 5412 water molecules in two groups: the first group contains 17 water molecules where at least one atom is within 3.55 Å of any cyanide atom, and the second group with all other water molecules. We then scale the point charges of each group with a factor between 0 and 1. In this way, we compute four different scenarios (and their interpolations): vacuum, only cyanide H bonds, only bulk solvent (but no cyanide H bonds), and full solvation (all). We show in the figure how the vertical excitation energy depends on the environment and on CT character.

In panels (a) and (d), we see that MC states (red) are virtually unaffected by the solvation shell. On the contrary, panels (b) and (e) show that MLCT states are strongly up-shifted in energy relative to the ground state in water. The LC states (bipyridine $\pi\pi^*$) in panels (c) and (f) are also not notably affected by solvent effects.

Comparing the top row with the bottom row shows that turning on the 17 water molecules close to the cyanides has a much stronger effect on the energies than turning on all 4995 other water molecules. This shows clearly that the MLCT destabilization in water is primarily due to explicit solvent interactions, i.e., the H bonds between water and the cyanides.

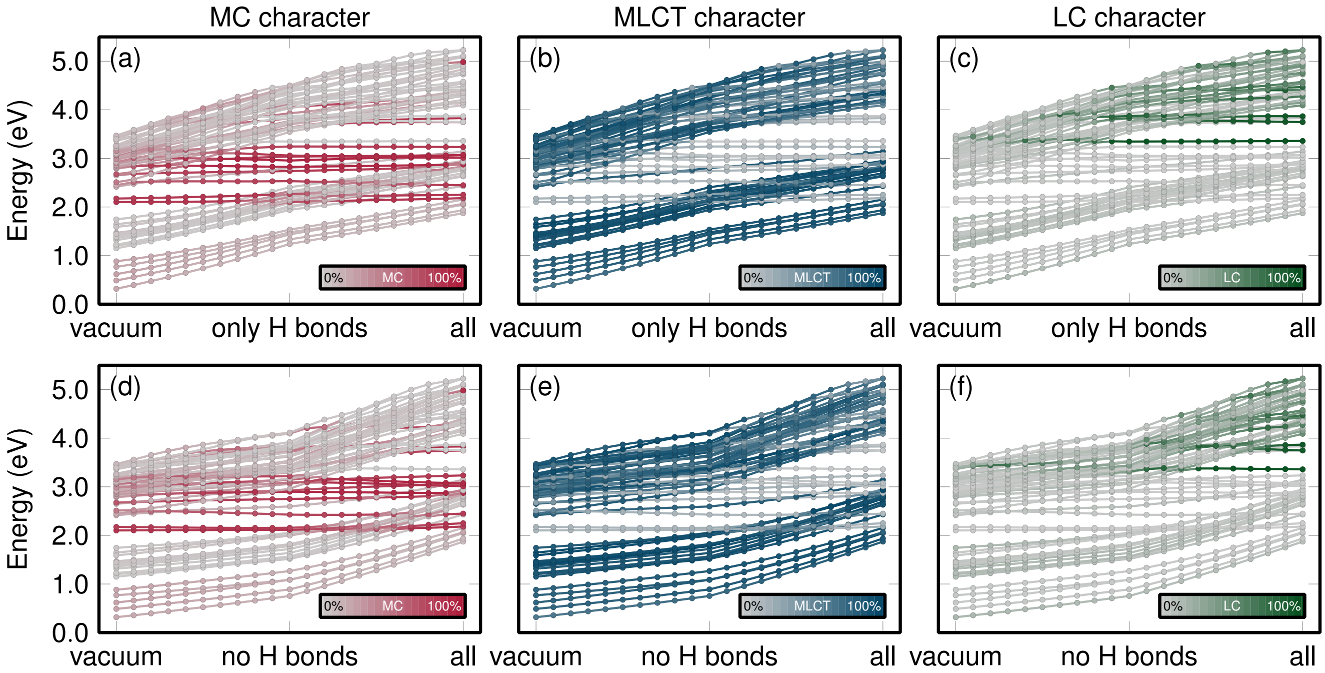

Figure S20: Scan of the vertical excitation energies of $[Fe(CN)_4(bpy)]^{2-}$ computed with QM/MM and scaling of the point charges. At the left of each plot, all point charges are zero (vacuum) and on the right all point charges are use the charges given in Section S1.2. In the top row, in the plot center the charges of 17 water molecules close to the cyanides are at full strength and all others are zero. In the bottom row, in the plot center the charges of those 17 water molecules are zero and all others are at full strength. Intermediate computations use interpolated point charges. The charge transfer character (using TheoDORE) is indicated by color.

The solvent shift can be explained by the relative changes in orbital energies, as shown in Figure S21 (corresponding to Figure S20a–c). As all absolute orbital energies are strongly lowered in solution, we show the orbital energies relative to the first unoccupied orbital for clarity. For reference, in absolute energies the t_{2g} orbitals are stabilized by about –5.5 eV from vacuum to full solvation, the cyanide π orbitals by about –6.2 eV, and the bipyridine π^* orbitals by –4.4 eV.

Several sets of orbitals can be identified. The orbitals localized on the Fe atom (panel (a)) are the five d orbitals, marked as t_{2g} (occupied) and e_g (virtual). In panel (b), two bunches of orbitals with large solvent shifts can be identified. Within the occupied block, we find eight cyanide π orbitals (with some d contributions) and—slightly lower—four cyanide N lone pairs. In the virtual block, we find eight cyanide π^* orbitals. In panel (c), bipy π and π^* orbitals can be seen, which have approximately constant energies relative to the lowest π^*. At higher energies, also several σ^*_{CH} orbitals of bipy occur, which are destabilized in solution due to their partial Rydberg character.

As described previously,[S50] the solvent shifts in the excitation energies can be explained by the strong electrostatic interaction of the negatively charged cyanides with the solvent. Due to backbonding, the cyanide π orbitals mix with the metal d orbitals, and therefore the interaction of the cyanides with the solvent stabilizes both the cyanide π orbitals and the metal d orbitals, where both occupied t_{2g} and virtual e_g levels are affected approximately equally. On the contrary, the bipyridine π and π^* orbitals are rather unaffected by solvent. This leads to an overall increase in MLCT energies, whereas the MC and LC states are not shifted relative to the ground state.

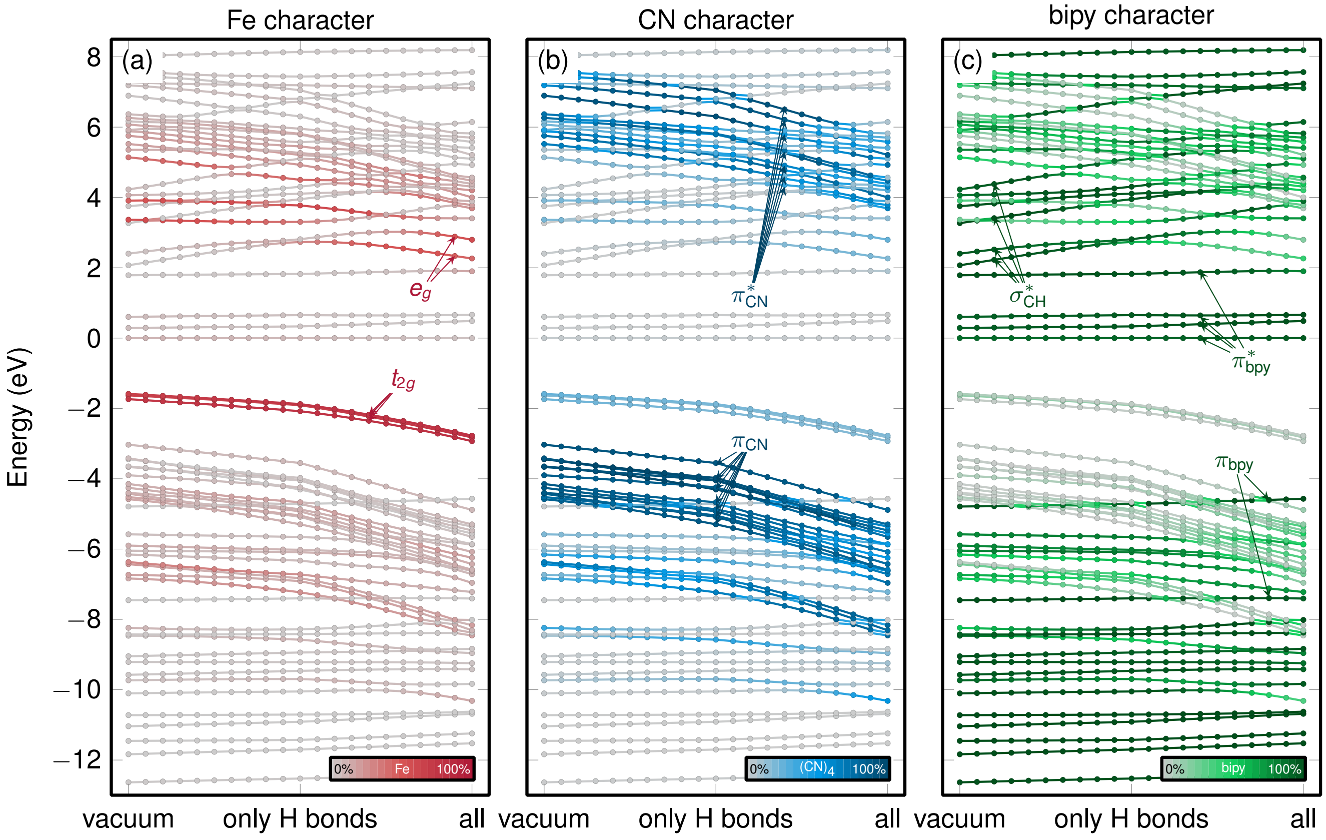

Figure S21: Scan of the orbital energies of $[Fe(CN)_4(bpy)]^{2-}$ computed with QM/MM and scaling of the point charges. The same calculations as in Figure S20a–c are presented. The orbital localization (Löwdin partitioning) is indicated by color. Orbital energies are relative to the energy of the first unoccupied orbital for clarity.

S4 Cartesian coordinates of optimized structures

```
29
water GS
C +0.351409 +2.638113 -0.027788
C +1.462096 +3.478766 -0.014935
C +0.351409 -2.638113 +0.027788
C +1.685488 -0.736725 +0.003472
C +1.685488 +0.736725 -0.003472
C +2.848846 +1.520957 +0.010973
C +2.848846 -1.520957 -0.010973
C +2.739110 -2.909404 -0.005855
C +1.462097 -3.478766 +0.014936
C +2.739110 +2.909404 +0.005855
N +0.451281 +1.298637 -0.020963
N +0.451281 -1.298637 +0.020964
H +3.831673 -1.050630 -0.029697
H +3.633955 -3.535107 -0.019025
H -0.666876 -3.029019 +0.041983
H +1.321191 +4.561158 -0.019433
H +1.321191 -4.561158 +0.019433
H +3.831673 +1.050630 +0.029697
H +3.633955 +3.535107 +0.019025
H -0.666876 +3.029019 -0.041983
Fe -1.098631 -0.000000 -0.000000
C -2.453120 +1.389560 -0.048387
N -3.242030 +2.260004 -0.084831
C -1.100552 -0.055981 -1.971112
N -1.071738 -0.088872 -3.143995
C -1.100552 +0.055981 +1.971112
N -1.071739 +0.088872 +3.143995
C -2.453120 -1.389560 +0.048386
N -3.242029 -2.260004 +0.084831
```

```
29
dmso GS
C +0.350604 +2.637707 -0.030248
C +1.461112 +3.478523 -0.016343
C +0.350604 -2.637707 +0.030248
C +1.685083 -0.736644 +0.003880
C +1.685083 +0.736645 -0.003880
C +2.848286 +1.521118 +0.011558
C +2.848286 -1.521117 -0.011558
C +2.738310 -2.909511 -0.006043
C +1.461113 -3.478523 +0.016343
C +2.738309 +2.909512 +0.006043
N +0.450702 +1.298223 -0.022829
N +0.450702 -1.298223 +0.022829
H +3.831124 -1.050808 -0.031647
H +3.632994 -3.535458 -0.020238
H -0.667861 -3.028098 +0.045556
H +1.319880 +4.560884 -0.021131
H +1.319881 -4.560883 +0.021131
H +3.831123 +1.050809 +0.031647
H +3.632993 +3.535459 +0.020238
H -0.667861 +3.028098 -0.045556
Fe -1.098810 +0.000000 +0.000000
C -2.452824 +1.390332 -0.052647
N -3.239762 +2.262377 -0.092135
C -1.100690 -0.061125 -1.971306
N -1.069639 -0.097158 -3.144013
C -1.100689 +0.061125 +1.971306
N -1.069638 +0.097158 +3.144013
C -2.452824 -1.390332 +0.052647
N -3.239762 -2.262377 +0.092135
```

```
29
acn GS
C +0.349990 +2.637415 -0.031622
C +1.460369 +3.478346 -0.017120
C +0.349991 -2.637415 +0.031622
C +1.684775 -0.736584 +0.004120
C +1.684774 +0.736585 -0.004120
C +2.847865 +1.521240 +0.011861
C +2.847865 -1.521239 -0.011861
C +2.737710 -2.909592 -0.006139
C +1.460370 -3.478346 +0.017120
C +2.737709 +2.909593 +0.006139
N +0.450260 +1.297919 -0.023888
N +0.450260 -1.297919 +0.023888
H +3.830715 -1.050941 -0.032693
H +3.632278 -3.535721 -0.020906
H -0.668613 -3.027419 +0.047535
H +1.318896 +4.560684 -0.022047
H +1.318897 -4.560684 +0.022047
H +3.830715 +1.050942 +0.032692
H +3.632278 +3.535721 +0.020906
H -0.668613 +3.027419 -0.047535
Fe -1.098923 +0.000000 +0.000000
C -2.452587 +1.390916 -0.055125
N -3.238067 +2.264156 -0.096371
C -1.100796 -0.064081 -1.971458
N -1.068083 -0.101921 -3.144043
C -1.100796 +0.064081 +1.971458
N -1.068083 +0.101921 +3.144043
C -2.452587 -1.390916 +0.055126
N -3.238068 -2.264155 +0.096370
```

```
29
water MLCT
C +0.370107 +2.658179 -0.000009
C +1.467762 +3.496417 -0.000011
C +0.370117 -2.658178 +0.000001
C +1.710140 -0.710618 +0.000000
C +1.710137 +0.710624 -0.000001
C +2.877042 +1.532675 +0.000000
C +2.877048 -1.532665 -0.000001
C +2.765862 -2.903460 +0.000000
C +1.467775 -3.496412 +0.000002
C +2.765851 +2.903470 -0.000005
N +0.453526 +1.312015 -0.000004
N +0.453531 -1.312014 +0.000000
H +3.862383 -1.064490 -0.000003
H +3.661712 -3.529262 -0.000001
H -0.643851 -3.061403 +0.000001
H +1.331435 +4.578593 -0.000016
H +1.331452 -4.578588 +0.000003
H +3.862380 +1.064504 +0.000005
H +3.661699 +3.529275 -0.000004
H -0.643863 +3.061401 -0.000012
Fe -1.071867 -0.000002 +0.000001
C -2.419353 +1.398368 +0.000000
N -3.232888 +2.237728 -0.000001
C -1.181118 -0.000006 -1.963709
N -1.194959 -0.000009 -3.132353
C -1.181109 +0.000003 +1.963712
N -1.194943 +0.000006 +3.132356
C -2.419348 -1.398377 +0.000007
N -3.232879 -2.237741 +0.000012
```

```
29
dmso MLCT
C +0.369973 +2.658068 -0.000006
C +1.467707 +3.496444 -0.000008
C +0.369967 -2.658069 +0.000001
C +1.709731 -0.710605 +0.000001
C +1.709733 +0.710601 -0.000003
C +2.876753 +1.532586 -0.000005
C +2.876750 -1.532592 +0.000003
C +2.765664 -2.903372 +0.000002
C +1.467700 -3.496447 +0.000000
C +2.765670 +2.903366 -0.000008
N +0.453298 +1.312209 -0.000004
N +0.453295 -1.312210 +0.000002
H +3.862046 -1.064297 +0.000005
H +3.661623 -3.529079 +0.000003
H -0.644055 -3.061165 +0.000001
H +1.331348 +4.578617 -0.000010
H +1.331339 -4.578620 -0.000001
H +3.862048 +1.064289 -0.000005
H +3.661630 +3.529072 -0.000010
H -0.644048 +3.061166 -0.000006
Fe -1.073204 +0.000001 +0.000001
C -2.419660 +1.398934 -0.000001
N -3.231408 +2.240041 -0.000002
C -1.180535 -0.000004 -1.963979
N -1.192906 -0.000006 -3.132643
C -1.180529 +0.000006 +1.963982
N -1.192896 +0.000009 +3.132646
C -2.419662 -1.398930 +0.000008
N -3.231412 -2.240035 +0.000012
```

```
29
acn MLCT
C +0.369876 +2.657983 -0.000014
C +1.467674 +3.496463 -0.000014
C +0.369842 -2.657988 +0.000001
C +1.709418 -0.710601 +0.000000
C +1.709428 +0.710580 +0.000000
C +2.876538 +1.532511 +0.000005
C +2.876518 -1.532547 +0.000000
C +2.765502 -2.903316 -0.000002
C +1.467630 -3.496481 -0.000001
C +2.765538 +2.903282 -0.000002
N +0.453129 +1.312357 -0.000007
N +0.453111 -1.312362 +0.000000
H +3.861785 -1.064165 -0.000002
H +3.661541 -3.528953 -0.000004
H -0.644224 -3.060980 +0.000002
H +1.331294 +4.578635 -0.000020
H +1.331237 -4.578651 -0.000001
H +3.861799 +1.064118 +0.000014
H +3.661585 +3.528908 +0.000002
H -0.644186 +3.060987 -0.000020
Fe -1.074225 +0.000005 +0.000001
C -2.419896 +1.399361 +0.000001
N -3.230290 +2.241787 +0.000001
C -1.180079 +0.000004 -1.964186
N -1.191332 +0.000003 -3.132866
C -1.180064 +0.000015 +1.964190
N -1.191311 +0.000021 +3.132869
C -2.419912 -1.399336 +0.000009
N -3.230316 -2.241752 +0.000015
```

```
29
water MC
C +0.354389 +2.681350 +0.020371
C +1.430106 +3.571224 +0.010581
C +0.649975 -2.642989 -0.014383
C +1.831961 -0.653154 +0.001081
C +1.748706 +0.833629 +0.001938
C +2.885693 +1.659998 -0.010950
C +3.053855 -1.348390 +0.013559
C +3.044722 -2.742431 +0.010023
C +1.817429 -3.408844 -0.005288
C +2.723421 +3.044456 -0.006368
N +0.508475 +1.354704 +0.016065
N +0.657659 -1.307560 -0.011043
H +4.003625 -0.814912 +0.027898
H +3.985060 -3.298216 +0.019913
H -0.340646 -3.105600 -0.024266
H +1.251754 +4.648133 +0.015627
H +1.759349 -4.498948 -0.008949
H +3.888052 +1.233580 -0.026270
H +3.596453 +3.700859 -0.016594
H -0.681115 +3.031734 +0.031194
Fe -1.333161 -0.064630 -0.001809
C -2.648811 +1.547582 +0.022227
N -3.410433 +2.439499 +0.037809
C -1.267342 -0.030724 -1.970953
N -1.187546 -0.006799 -3.141682
C -1.276785 -0.089976 +1.967563
N -1.203915 -0.102026 +3.138871
C -2.448112 -1.815075 -0.029839
N -3.106468 -2.785842 -0.044147
```

```
29
dmso MC
C +0.420651 +2.676175 -0.026936
C +1.517075 +3.540432 -0.015743
C +0.590570 -2.653696 +0.036643
C +1.818221 -0.692091 +0.005313
C +1.770226 +0.796226 -0.000152
C +2.926723 +1.595196 +0.015031
C +3.023360 -1.415753 -0.014688
C +2.981989 -2.809149 -0.006334
C +1.739719 -3.446705 +0.021177
C +2.797434 +2.983132 +0.006783
N +0.543116 +1.346268 -0.019156
N +0.628925 -1.318901 +0.028739
H +3.985097 -0.904446 -0.039553
H +3.908872 -3.386956 -0.022191
H -0.410425 -3.093208 +0.054813
H +1.364188 +4.621265 -0.023391
H +1.656108 -4.535109 +0.029010
H +3.918620 +1.145269 +0.035811
H +3.685951 +3.618406 +0.018646
H -0.606135 +3.050990 -0.042548
Fe -1.341058 -0.037536 -0.000215
C -2.602082 +1.612275 -0.055149
N -3.336735 +2.526164 -0.082585
C -1.273685 -0.094457 -1.969049
N -1.193278 -0.128636 -3.139383
C -1.293988 +0.025764 +1.969134
N -1.226572 +0.068100 +3.140027
C -2.485576 -1.768043 +0.043907
N -3.160968 -2.726726 +0.063124
```

```
29
acn MC
C +0.411724 +2.677085 -0.030926
C +1.505282 +3.544963 -0.017894
C +0.599621 -2.652166 +0.047797
C +1.820320 -0.686468 +0.008654
C +1.767335 +0.801700 +0.001796
C +2.921206 +1.604472 +0.019424
C +3.027883 -1.405933 -0.017048
C +2.991380 -2.799442 -0.007214
C +1.751473 -3.441209 +0.027508
C +2.787399 +2.991959 +0.009113
N +0.538539 +1.347639 -0.020988
N +0.633364 -1.317280 +0.038325
H +3.987627 -0.891206 -0.048226
H +3.920165 -3.374079 -0.027808
H -0.399828 -3.095006 +0.070633
H +1.348756 +4.625270 -0.027442
H +1.671647 -4.529896 +0.036769
H +3.914475 +1.157729 +0.044169
H +3.673778 +3.630203 +0.022944
H -0.616306 +3.048310 -0.050050
Fe -1.343452 -0.041637 -0.000766
C -2.605788 +1.605994 -0.072375
N -3.340844 +2.519275 -0.108058
C -1.266740 -0.112393 -1.968863
N -1.178517 -0.155633 -3.138327
C -1.303869 +0.036116 +1.968623
N -1.239525 +0.088004 +3.139298
C -2.478736 -1.777556 +0.050621
N -3.147516 -2.740790 +0.073125
```

```
29
water GS from MD
C +1.612729 -0.941294 +2.759624
C +0.559023 -1.184725 +3.632527
C +1.487421 -1.243434 -2.523752
C +0.263810 -1.478820 -0.569789
C +0.297830 -1.391850 +0.904752
C -0.801788 -1.648158 +1.726573
C -0.868195 -1.837219 -1.303944
C -0.800129 -1.896113 -2.691269
C +0.398658 -1.594796 -3.311649
C -0.670328 -1.542821 +3.106886
N +1.493301 -1.038605 +1.428356
N +1.429989 -1.184085 -1.186430
H -1.779272 -2.066135 -0.798884
H -1.659554 -2.170478 -3.271450
H +2.433915 -1.000913 -2.960285
H +0.706667 -1.092532 +4.692299
H +0.496841 -1.628986 -4.380515
H -1.738003 -1.923767 +1.293321
H -1.504744 -1.735799 +3.753600
H +2.581058 -0.659782 +3.121074
Fe +2.965297 -0.681580 +0.059345
C +4.282303 -0.244125 +1.362574
N +5.063760 +0.018288 +2.173153
C +3.500840 -2.534911 +0.141475
N +3.795090 -3.641097 +0.188681
C +2.425022 +1.170878 -0.021963
N +2.078256 +2.261054 -0.067363
C +4.207962 -0.376166 -1.352021
N +4.940998 -0.191310 -2.226490
```

```
29
water MLCT from MD
C +1.608480 -0.934851 +2.783410
C +0.558134 -1.189520 +3.658300
C +1.483670 -1.254390 -2.548470
C +0.259505 -1.476120 -0.552180
C +0.294317 -1.395560 +0.886650
C -0.812197 -1.666110 +1.734460
C -0.887695 -1.824850 -1.309900
C -0.821393 -1.884750 -2.687160
C +0.393154 -1.597200 -3.337590
C -0.681051 -1.563920 +3.105810
N +1.497450 -1.028960 +1.454550
N +1.435470 -1.190440 -1.214580
H -1.804470 -2.040570 -0.805361
H -1.691010 -2.147640 -3.260210
H +2.432740 -1.022550 -2.997740
H +0.700617 -1.098590 +4.719070
H +0.485621 -1.636030 -4.407110
H -1.749450 -1.951780 +1.302470
H -1.520290 -1.769670 +3.747950
H +2.575470 -0.643656 +3.158010
Fe +2.990500 -0.671123 +0.056384
C +4.321020 -0.221484 +1.360700
N +5.115410 +0.043788 +2.147060
C +3.491510 -2.546060 +0.153834
N +3.786340 -3.648540 +0.208950
C +2.402860 +1.178450 -0.038734
N +2.055340 +2.264270 -0.092223
C +4.259570 -0.370628 -1.352320
N +5.019080 -0.190408 -2.195720
```

```
29
water MC from MD
C +1.532230 -0.933274 +2.798280
C +0.477052 -1.192650 +3.658190
C +1.411550 -1.251190 -2.553940
C +0.230856 -1.489450 -0.568760
C +0.265464 -1.407420 +0.912404
C -0.838789 -1.690880 +1.720200
C -0.911988 -1.849620 -1.286230
C -0.865999 -1.905650 -2.674180
C +0.318569 -1.601230 -3.327350
C -0.730696 -1.581560 +3.101760
N +1.432100 -1.037560 +1.470190
N +1.371790 -1.195900 -1.219410
H -1.813440 -2.079040 -0.768797
H -1.733510 -2.179220 -3.234850
H +2.351550 -1.007200 -3.008580
H +0.601660 -1.092170 +4.721270
H +0.398018 -1.631310 -4.398610
H -1.758880 -1.988860 +1.275960
H -1.568240 -1.793230 +3.732210
H +2.489370 -0.633680 +3.176620
Fe +3.152150 -0.625388 +0.045668
C +4.452620 -0.188262 +1.535380
N +5.202240 +0.058756 +2.345050
C +3.626040 -2.506870 +0.133233
N +3.860820 -3.617140 +0.179666
C +2.564450 +1.222070 -0.045459
N +2.174460 +2.287500 -0.096457
C +4.391260 -0.357736 -1.547780
N +5.116140 -0.210399 -2.397070
```

Supplementary References

(S1) Frisch, M. J.; Trucks, G. W.; Schlegel, H. B.; Scuseria, G. E.; Robb, M. A.; Cheeseman, J. R.; Scalmani, G.; Barone, V.; Petersson, G. A.; Nakatsuji, H.; Li, X.; Caricato, M.; Marenich, A. V.; Bloino, J.; Janesko, B. G.; Gomperts, R.; Mennucci, B.; Hratchian, H. P.; Ortiz, J. V.; Izmaylov, A. F.; Sonnenberg, J. L.; Williams-Young, D.; Ding, F.; Lipparini, F.; Egidi, F.; Goings, J.; Peng, B.; Petrone, A.; Henderson, T.; Ranasinghe, D.; Zakrzewski, V. G.; Gao, J.; Rega, N.; Zheng, G.; Liang, W.; Hada, M.; Ehara, M.; Toyota, K.; Fukuda, R.; Hasegawa, J.; Ishida, M.; Nakajima, T.; Honda, Y.; Kitao, O.; Nakai, H.; Vreven, T.; Throssell, K.; Montgomery Jr., J. A.; Peralta, J. E.; Ogliaro, F.; Bearpark, M. J.; Heyd, J. J.; Brothers, E. N.; Kudin, K. N.; Staroverov, V. N.; Keith, T. A.; Kobayashi, R.; Normand, J.; Raghavachari, K.; Rendell, A. P.; Burant, J. C.; Iyengar, S. S.; Tomasi, J.; Cossi, M.; Millam, J. M.; Klene, M.; Adamo, C.; Cammi, R.; Ochterski, J. W.; Martin, R. L.; Morokuma, K.; Farkas, O.; Foresman, J. B.; Fox, D. J. Gaussian 16 Revision C.01. 2016; `https://gaussian.com`.

(S2) Miertuš, S.; Scrocco, E.; Tomasi, J. Electrostatic interaction of a solute with a continuum. A direct utilizaion of AB initio molecular potentials for the prevision of solvent effects. *Chem. Phys.* **1981**, *55*, 117–129.

(S3) Mennucci, B. Polarizable continuum model. *Wiley Interdiscip. Rev. Comput. Mol. Sci.* **2012**, *2*, 386–404.

(S4) Grimme, S.; Antony, J.; Ehrlich, S.; Krieg, H. A consistent and accurate ab initio parametrization of density functional dispersion correction (DFT-D) for the 94 elements H-Pu. *J. Chem. Phys.* **2010**, *132*, 154104.

(S5) Plasser, F.; Ruckenbauer, M.; Mai, S.; Oppel, M.; Marquetand, P.; González, L. Efficient and Flexible Computation of Many-Electron Wave Function Overlaps. *J. Chem. Theory Comput.* **2016**, *12*, 1207–1219.

(S6) Reiher, M.; Salomon, O.; Hess, B. A. Reparameterization of hybrid functionals based on energy differences of states of different multiplicity. *Theor. Chem. Acc.* **2001**, *107*, 48–55.

(S7) Mai, S.; Gattuso, H.; Monari, A.; González, L. Novel Molecular-Dynamics-Based Protocols for Phase Space Sampling in Complex Systems. *Front. Chem.* **2018**, *6*, 1–14.

(S8) Case, D.; Cerutti, D.; Cheatham, T.; III,; Darden, T.; Duke, R.; Giese, T.; Gohlke, H.; Goetz, A.; Greene, D.; Hornmeyer, N.; Izadi, S.; Kovalenko, A.; Lee, T. S.; LeGrand, S.; Li, P.; Lin, C.; Liu, J.; Luchko, T.; Luo, R.; Mermelstein, D.; Merz, K.; Monard, G.; Nguyen, H.; Omelyan, I.; Onufriev, A.; Pan, F.; Qi, R.; Roe, D. R.; Roitberg, A.; Sagui, C.; Simmerling, C.; Botello-Smith, W.; Swails, J.; Walker, R.; Wang, J.; Wolf, R.; Wu, X.; Xiao, L.; York, D.; Kollman, P. AMBER 2017. 2017.

(S9) Li, P.; Merz, K. M. MCPB.py: A Python Based Metal Center Parameter Builder. *J. Chem. Inf. Model.* **2016**, *56*, 599–604.

(S10) Lee, Chengteh; Yang, Weitao; Parr, R. G. Development of the Colle-Salvetti correlation energy formula into a functional of the electron density. *Phys. Rev. B.* **1988**, *37*, 785–789.

(S11) Becke, A. D. A new mixing of Hartree-Fock and local density-functional theories. *J. Chem. Phys.* **1993**, *98*, 1372–1377.

(S12) Hay, P. J.; Wedt, W. R. Ab initio effective core potentials for molecular calculations. Potentials of K to Au including the outermost core orbitals. *J. Chem. Phys* **1985**, *82*, 299–310.

(S13) Seminario, J. M. Calculation of Intramolecular Force Fields from Second-Derivative Tensors. *Int. J. Quantum Chem.* **1996**, *60*, 1271–1277.

(S14) Bayly, C. I.; Cieplak, P.; Cornell, W. D.; Kollman, P. A. A well-behaved electrostatic potential based method using charge restraints for deriving atomic charges: The RESP model. *J. Phys. Chem.* **1993**, *97*, 10269–10280.

(S15) Frisch, M. J.; Trucks, G. W.; Schlegel, H. B.; Scuseria, G. E.; Robb, M. A.; Cheeseman, J. R.; Al., E. Gaussian 09. 2016.

(S16) Wu, Y.; Tepper, H. L.; Voth, G. A. Flexible simple point-charge water model with improved liquid-state properties. *J. Chem. Phys.* **2006**, *124*, 024503–1–024503–12.

(S17) Götz, A. W.; Clark, M. A.; Walker, R. C. An extensible interface for QM/MM molecular dynamics simulations with AMBER. *J. Comput. Chem.* **2014**, *35*, 95–108.

(S18) Salomon, O.; Reiher, M.; Hess, B. A. Assertion and validation of the performance of the B3LYP* functional for the first transition metal row and the G2 test set. *J. Chem. Phys.* **2002**, *117*, 4729–4737.

(S19) Saureu, S.; Graaf, C. D. TD-DFT study of the light-induced spin crossover. *Phys. Chem. Chem. Phys.* **2016**, *18*, 1233–1244.

(S20) Pápai, M.; Vankó, G.; Graaf, C. D.; Rozgonyi, T. Theoretical Investigation of the Electronic Structure of Fe (II) Complexes at Spin-State Transitions. *J. Chem. Theory Comput.* **2013**, *9*, 509–519.

(S21) Weigend, F.; Ahlrichs, R. Balanced basis sets of split valence, triple zeta valence and quadruple zeta valence quality for H to Rn: Design and assessment of accuracy. *Phys. Chem. Chem. Phys.* **2005**, *7*, 3297–3305.

(S22) van Lenthe, E.; Baerends, E. J.; Snijders, J. G. Relativistic regular two-component Hamiltonians. *J. Chem. Phys.* **1993**, *99*, 4597.

(S23) Barbatti, M.; Sen, K. Effects of different initial condition samplings on photodynamics and spectrum of pyrrole. *Int. J. Quantum Chem.* **2016**, *116*, 762–771.

(S24) Klaffki, N.; Weingart, O.; Garavelli, M.; Spohr, E. Sampling excited state dynamics: Influence of HOOP mode excitations in a retinal model. *Phys. Chem. Chem. Phys.* **2012**, *14*, 14299–14305.

(S25) Zobel, J. P.; Heindl, M.; Nogueira, J. J.; González, L. Vibrational Sampling and Solvent Effects on the Electronic Structure of the Absorption Spectrum of 2-Nitronaphthalene. *J. Chem. Theory Comput.* **2018**, *14*, 3205–3217.

(S26) Barbatti, M.; Lischka, H. Can the nonadiabatic photodynamics of aminopyrimidine be a model for the ultrafast deactivation of adenine? *J. Phys. Chem. A* **2007**, *111*, 2852–2858.

(S27) Kjær, K. S.; Kunnus, K.; Harlang, T. C.; Van Driel, T. B.; Ledbetter, K.; Hartsock, R. W.; Reinhard, M. E.; Koroidov, S.; Li, L.; Laursen, M. G.; Biasin, E.; Hansen, F. B.; Vester, P.; Christensen, M.; Haldrup, K.; Nielsen, M. M.; Chabera, P.; Liu, Y.; Tatsuno, H.; Timm, C.; Uhlig, J.; Sundstöm, V.; Németh, Z.; Szemes, D. S.; Bajnóczi, É.; Vankó, G.; Alonso-Mori, R.; Glownia, J. M.; Nelson, S.; Sikorski, M.; Sokaras, D.; Lemke, H. T.; Canton, S. E.; Wärnmark, K.; Persson, P.; Cordones, A. A.; Gaffney, K. J. Solvent control of charge transfer excited state relaxation pathways in [Fe(2,2'-bipyridine)(CN)4]2-. *Phys. Chem. Chem. Phys.* **2018**, *20*, 4238–4249.

(S28) Kunnus, K.; Li, L.; Titus, C. J.; Lee, S. J.; Reinhard, M. E.; Koroidov, S.; Kjær, K. S.; Hong, K.; Ledbetter, K.; Doriese, W. B.; O'Neil, G. C.; Swetz, D. S.; Ullom, J. N.; Li, D.; Irwin, K.; Nordlund, D.; Cordones, A. A.; Gaffney, K. J. Chemical control of competing electron transfer pathways in iron tetracyano-polypyridyl photosensitizers. *Chem. Sci.* **2020**, *11*, 4360–4373.

(S29) Kossmann, S.; Neese, F. Efficient Structure Optimization with Second-Order Many-Body Perturbation Theory: The RIJCOSX-MP2 Method. *J. Chem. Theory Comput.* **2010**, *6*, 2325–2338.

(S30) Pantazis, D. A.; Chen, X. Y.; Landis, C. R.; Neese, F. All-electron scalar relativistic basis sets for third-row transition metal atoms. *J. Chem. Theory Comput.* **2008**, *4*, 908–919.

(S31) Hirata, S.; Head-Gordon, M. Time-dependent density functional theory within the Tamm-Dancoff approximation. *Chem. Phys. Lett.* **1999**, *314*, 291–299.

(S32) Rackers, J.; Wang, Z.; Lu, C.; Laury, M. L.; Lagardere, L.; Schnieders, M. J.; Piquemal, J.-P.; Ren, P.; Ponder, J. W. Tinker - Software Tools for Molecular Design. 2018; `http://dasher.wustl.edu/tinker/`.

(S33) Granucci, G.; Persico, M.; Toniolo, A. Direct semiclassical simulation of photochemical processes with semiempirical wave functions. *J. Chem. Phys.* **2001**, *114*, 10608.

(S34) Mai, S.; Marquetand, P.; González, L. Nonadiabatic dynamics: The SHARC approach. *Wiley Interdiscip. Rev. Comput. Mol. Sci.* **2018**, *e1370*, 1–23.

(S35) Granucci, G.; Persico, M. Critical appraisal of the fewest switches algorithm for surface hopping. *J. Chem. Phys.* **2007**, *126*, 134114.

(S36) Mai, S.; Marquetand, P.; González, L. A general method to describe intersystem crossing dynamics in trajectory surface hopping. *Int. J. Quantum Chem.* **2015**, *115*, 1215–1231.

(S37) Plasser, F. TheoDORE: A package for theoretical density, orbital relaxation, and exciton analysis. 2017; `http://theodore-qc.sourceforge.net`.

(S38) Plasser, F.; Lischka, H. Analysis of Excitonic and Charge Transfer Interactions from Quantum Chemical Calculations. *J. Chem. Theory Comput.* **2012**, *8*, 2777–2789.

(S39) Mai, S.; Plasser, F.; Dorn, J.; Fumanal, M.; Daniel, C.; González, L. Quantitative wave function analysis for excited states of transition metal complexes. *Coord. Chem. Rev.* **2018**, *361*, 74–97.

(S40) Schmitz, M.; Tavan, P. Vibrational spectra from atomic fluctuations in dynamics simulations. I. Theory, limitations, and a sample application. *J. Chem. Phys.* **2004**, *121*, 12233.

(S41) Ihee, H.; Wulff, M.; Kim, J. Ultrafast X-ray scattering : structural dynamics from diatomic to protein molecules. *Int. Rev. Phys. Chem.* **2010**, *29*, 453–520.

(S42) Haldrup, K.; Christensen, M.; Meedom Nielsen, M. Analysis of time-resolved X-ray scattering data from solution-state systems. *Acta Crystallogr. Sect. A Found. Crystallogr.* **2010**, *66*, 261–269.

(S43) Debye, P. Zerstreuung von Röntgenstrahlen. *Ann. Phys.* **1915**, *351*, 809–823.

(S44) Dohn, A. O.; Biasin, E.; Haldrup, K.; Nielsen, M. M.; Henriksen, N. E.; Møller, K. B. On the calculation of x-ray scattering signals from pairwise radial distribution functions. *J. Phys. B At. Mol. Opt. Phys.* **2015**, *48*, 244010.

(S45) Dhabal, D.; Wikfeldt, K. T.; Skinner, L. B.; Chakravarty, C.; Kashyap, H. K. Probing the triplet correlation function in liquid water by experiments and molecular simulations. *Phys. Chem. Chem. Phys.* **2017**, *19*, 3265–3278.

(S46) Waser, J.; Schomaker, V. The fourier inversion of diffraction data. *Rev. Mod. Phys.* **1953**, *25*, 671–690.

(S47) Wikfeldt, K. T.; Huang, C.; Nilsson, A.; Pettersson, L. G. Enhanced small-angle scattering connected to the Widom line in simulations of supercooled water. *J. Chem. Phys.* **2011**, *134*, 214506–1 – 214506–16.

(S48) Mai, S.; González, L. Unconventional two-step spin relaxation dynamics of [Re(CO)3(im)(phen)]+ in aqueous solution. *Chem. Sci.* **2019**, *10*, 10405–10411.

(S49) Haldrup, K.; Christensen, M.; Nielsen, M. M. Analysis of time-resolved X-ray scattering data from solution-state systems. *Acta Cryst. A* **2010**, *66*, 261–269.

(S50) Toma, H. E.; Takasugi, M. S. Spectroscopic studies of preferential and asymmetric solvation in substituted cyanoiron(II) complexes. *J. Solution Chem.* **1983**, *12*, 547–561.
